# Site-selective bromination of sp^3^ C–H bonds†
†Electronic supplementary information (ESI) available. See DOI: 10.1039/c7sc04611a


**DOI:** 10.1039/c7sc04611a

**Published:** 2017-11-20

**Authors:** Shyam Sathyamoorthi, Shibdas Banerjee, J. Du Bois, Noah Z. Burns, Richard N. Zare

**Affiliations:** a Stanford University , Department of Chemistry , 333 Campus Drive , Stanford , CA 94305-4401 , USA . Email: zare@stanford.edu ; Email: jdubois@stanford.edu ; Email: nburns@stanford.edu; b Indian Institute of Science Education and Research Tirupati , Karakambadi Road , Tirupati-517507 , India

## Abstract

A method for converting sp^3^ C–H to C–Br bonds using an *N*-methyl sulfamate directing group is described. The reaction employs Rh_2_(oct)_4_ and a mixture of NaBr and NaOCl and is performed in aqueous solution open to air. For all sulfamates examined, oxidation occurs with high selectivity at the γ-carbon, affording a uniquely predictable method for C–H bond halogenation. Results from a series of mechanistic experiments suggest that substrate oxidation likely proceeds by a radical chain process. Initial formation of an *N*-halogenated sulfamate followed by Rh-mediated homolysis generates an N-centered radical, which serves as the active oxidant.

## Abstract 2

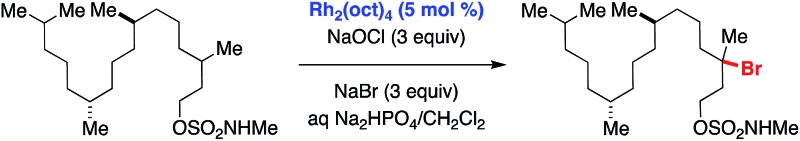
A method for converting sp^3^ C–H to C–Br bonds using an *N*-methyl sulfamate directing group is described. For all sulfamates examined, bromination occurs with high selectivity at the γ-carbon, affording a predictable method for C–H bond halogenation.

## Introduction

Oxidation of a specific sp^3^ C–H bond in a complex molecule remains an outstanding challenge in reaction methods development.^1^–^3^ While several protocols for the selective conversion of sp^3^ C–H centers to C–N and C–O bonds are now available,^4^–^16^ fewer methods for the synthesis of C–halogen bonds^17^–^36^ have been described despite the fact that molecules bearing halogen functional groups are prevalent in nature. In addition, as alkyl halides are versatile precursors for a variety of synthetic transformations, including cross-coupling, substitution, elimination, and the installation of boron-, silicon-, nitrogen-, and oxygen-based groups, methods for accessing these types of materials have value in synthesis.^37^–^41^ Here, we describe a reaction protocol for the site-selective bromination of sp^3^ C–H bonds using an *N*-methyl sulfamate directing group. This auxiliary is facile to install on 1° and 2° alcohol derivatives and can be removed through nucleophilic displacement. Mechanistic studies suggest that the reaction proceeds through an N-centered radical, reminiscent of the Hoffman–Löffler–Freytag amine synthesis.^42^–^55^ With all sulfamates tested, oxidation occurs preferentially at the γ-carbon, offering a predictable and precise method for oxidative C–H bond halogenation under mild reaction conditions.

## Results and discussion

Initial optimization of conditions for alkylbromide formation was performed with isopentyl methylsulfamate **1**, a simple, unfunctionalized substrate with a single tertiary C–H bond. A variety of transition metal salts were tested in conjunction with 3 equivalents each of NaBr and NaOCl, a reagent combination known to generate hypobromite *in situ* and used previously for the oxidative cyclization of sulfamate esters.^56^ While little to no reaction ensued in the presence of catalytic Mn^3+^, Co^2+^, Cu^2+^, and Ni^2+^ (Table 1, entries 1–4), switching to Rh_2_(oct)_4_ (Table 1, entry 5) afforded a marked increase in conversion to brominated product **2**. The choice of dirhodium complex had a clear influence on reaction performance, as catalysts bearing hydrophobic ligands such as octanoate or triphenylacetate (Table 1, entries 5–7) out-performed others tested (Table 1, entries 8, 9), likely owing to the greater solubility of these complexes in dichloromethane. In the complete absence of transition metal, conversion to product still occurred (Table 1, entry 10) in a process that appears to be promoted by ambient light (Table 1, entry 11).

A 1 : 1 biphasic mixture of CH_2_Cl_2_/saturated aqueous Na_2_HPO_4_ was found to be the optimal solvent combination (Table 1, entry 5). In neat CH_2_Cl_2_, conversion to product was significantly reduced, and in unbuffered water with CH_2_Cl_2_ a mixture of both brominated and chlorinated products was obtained (Table 1, entries 12 and 13). Using organic co-solvents other than CH_2_Cl_2_ was similarly deleterious to reaction performance (Table 1, entries 14 and 15).

**Table 1:** Evaluating reaction conditions for directed C–H bromination

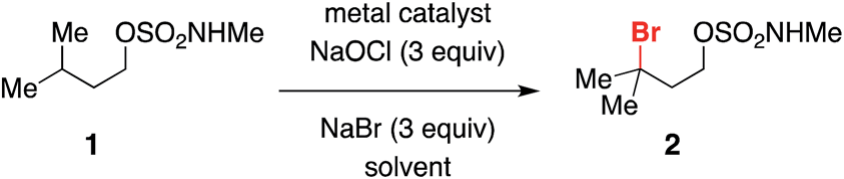			
Entry	Catalyst	Solvent^*a*^	[2]/[1]^*b*^
1	(*R*,*R*)-Mn-Jacobsen (5%)	CH_2_Cl_2_	RSM
2	Co(OAc)_2_·4H_2_O (5%)	CH_2_Cl_2_	1/8
3	CuBr_2_ (5%)	CH_2_Cl_2_	1/8
4	Ni(OAc)_2_·4H_2_O (5%)	CH_2_Cl_2_	RSM
**5**	**Rh**_**2**_ **(oct)**_**4**_ **(5%)**	**CH**_**2**_ **Cl**_**2**_	**4/1**
6	Rh_2_(O_2_C^*t*^Bu)_4_ (5%)	CH_2_Cl_2_	2/1
**7**	**Rh**_**2**_ **(O**_**2**_ **CCPh**_**3**_ **)**_**4**_ **(5%)**	**CH**_**2**_ **Cl**_**2**_	**4/1**
8	Rh_2_(OAc)_4_ (5%)	CH_2_Cl_2_	1/5
9	Na_4_Rh_2_(CO_3_)_4_ (5%)	CH_2_Cl_2_	1/7
10	None	CH_2_Cl_2_	1/4
11	None^*c*^	CH_2_Cl_2_	RSM
12	Rh_2_(oct)_4_ (5%)	CH_2_Cl_2_^*d*^	1/3
13	Rh_2_(oct)_4_ (5%)	CH_2_Cl_2_^*e*^	1/2^*f*^
14	Rh_2_(oct)_4_ (5%)	iPrOAc	1/2
15	Rh_2_(oct)_4_ (5%)	Benzene	1/2

^*a*^All reactions were performed in a biphasic solvent mixture with the indicated solvent and an equivalent volume of saturated aqueous Na_2_HPO_4_ unless otherwise noted.

^*b*^Product ratio determined by ^1^H NMR integration, see ESI for details.

^*c*^Reaction flask wrapped in foil.

^*d*^Reaction performed with no added co-solvent.

^*e*^Reaction conducted with an equivalent volume of deionized H_2_O.

^*f*^A small amount of the corresponding chloride product is also formed. RSM = recovered starting material.

A range of structurally diverse *N*-methyl sulfamates has been prepared by condensation of the corresponding 1° and 2° alcohols with ClSO_2_NHMe and subjected to the optimized halogenation protocol (Table 2). Oxidation of both tertiary and benzylic C–H bonds is possible in moderate to good yields, even with Rh_2_(oct)_4_ loadings as low as 0.1 mol% (entry 2). Different functional groups, including alkyl and benzyl esters, epoxides, trichloroethylsulfamate-protected aziridines, and silylated alcohols, are compatible with these conditions (Table 2, entries 3–6). For all substrates examined, C–H bond oxidation occurs nearly exclusively at the γ-carbon. This finding compares favorably with other directed C–H halogenation methods, which afford mixtures of constitutional isomers.^15^,^20^ The directed nature of this process is further highlighted in entries 8, 9, and 10. Each of these substrates furnishes the product of γ-C–H bond bromination despite possessing an activated benzylic C–H center. Positional selectivity is also noted in entries 11 and 12. Experiments with the latter substrate show that oxidation of an optically active 3° C–H bond gives racemic alkylbromide, a result consistent with the formation of a carbon-centered radical intermediate (*vide infra*). In addition, we have found that the absence of NaBr leads to generation of the corresponding chlorinated product (entry 12), albeit in reduced yield. All told, this new protocol offers an efficient, predictable, operationally simple method for C–H bond functionalization.

**Table 2:** Oxidative halogenation of *N*-methyl sulfamate derivatives

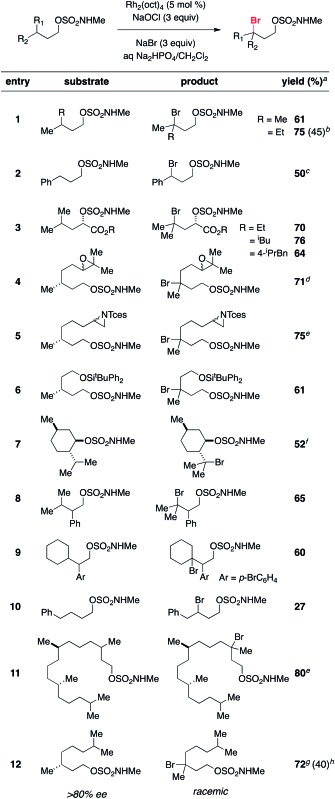			

^*a*^Isolated product yield unless otherwise indicated.

^*b*^Reaction performed with 0.1 mol% Rh_2_(oct)_4_.

^*c*^Yield estimated by ^1^H NMR integration using an internal standard.

^*d*^Product isolated as a 1 : 1 mixture of diastereomers.

^*e*^Product isolated as a mixture of diastereomers, ratio undetermined.

^*f*^Product yield estimated by ^1^H NMR integration using an internal standard. Chromatography on SiO_2_ facilitates bromide elimination, see Fig. S1 for details.

^*g*^Product isolated as a racemic mixture, see Fig. S2 for details.

^*h*^Yield of corresponding chloride product obtained from a reaction performed without NaBr.

**Scheme 1:**
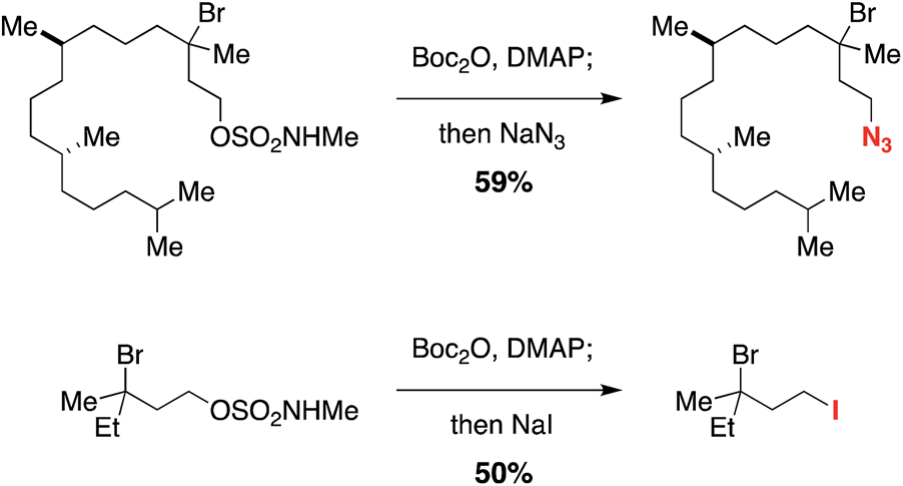
Representative sulfamate displacement reactions.

Displacement of the *N*-methyl alkoxysulfonyl auxiliary can be achieved in a single-flask, two-step protocol that involves initial *N*-carbamoylation with Boc_2_O followed by an S_N_2 reaction (Scheme 1). The *N*-acylated sulfamate undergoes smooth reaction with nucleophiles such as N_3_^–^ and I^–^ to give the corresponding alkylazide and alkyliodide products, respectively. This method for excising the sulfamate directing group should add to the overall utility of the C–H halogenation process.

**Scheme 2:**
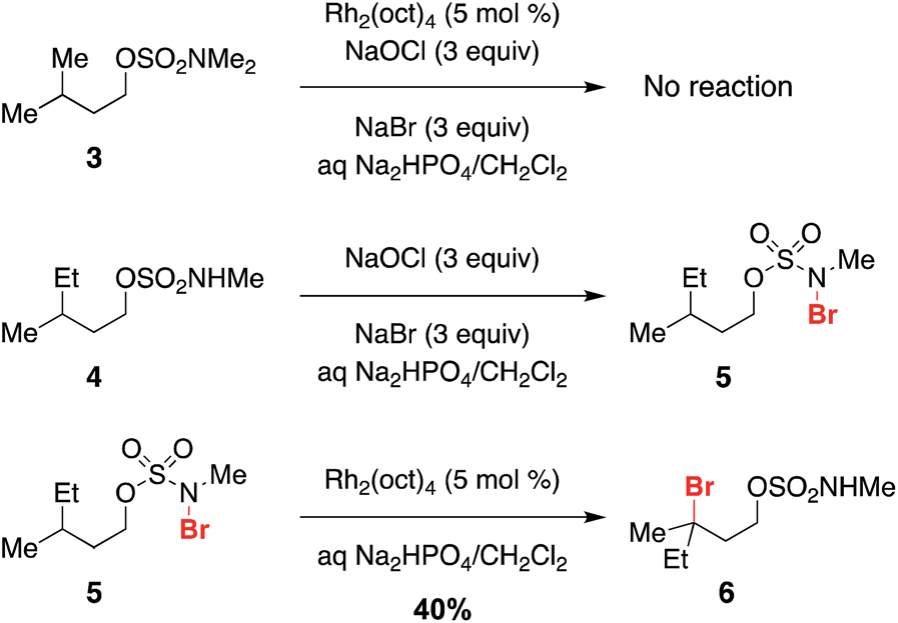
*N*-Bromo sulfamate **5** is a chemically competent intermediate.

Previous work exploring the use of NaBr/NaOCl for the synthesis of [1,2,3]-oxathiazinane-2,2-dioxide heterocycles^56^ suggested the formation of an *N*-halogenated species as a first step in the reaction pathway. In accord with this hypothesis, we have demonstrated that the *N*,*N*-dimethyl sulfamate **3** is not a competent substrate for oxidation. Additionally, we have prepared an *N*-brominated sulfamate **5** and have shown that this compound will react with 5 mol% Rh_2_(oct)_4_ to form alkylbromide **6** in 40% yield (Scheme 2 and Fig. S3†). Although the efficiency of this process is reduced from that of the catalytic protocol (entry 1, Table 2), these findings establish the *N*-brominated species as a chemically competent intermediate on the reaction pathway.

**Fig. 1:**
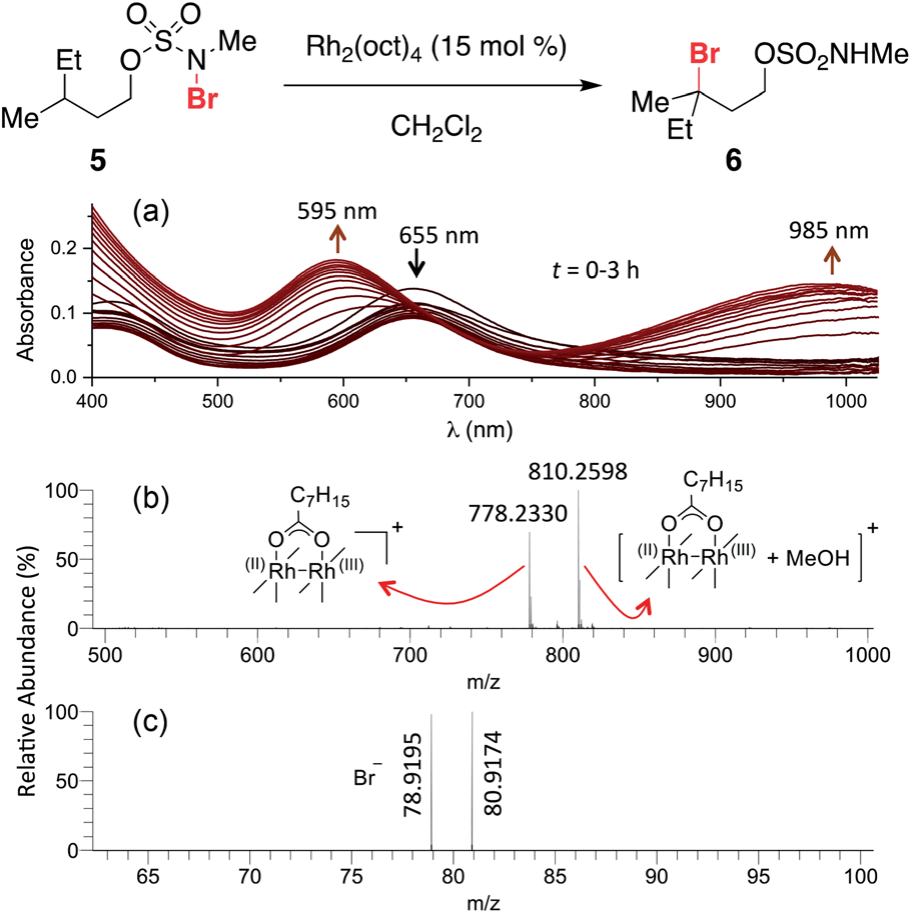
(a) Continuous UV/vis spectrophotometric monitoring of the reaction of **5** shows an absorption spectrum characteristic of the conversion of a dinuclear Rh^2+^/Rh^2+^ complex (*λ*_max_ = 418 nm and 655 nm) to a Rh^2+^/Rh^3+^ complex (*λ*_max_ = 595 nm and 985 nm). High-resolution ESI-MS detected ion signals of (b) Rh^2+^/Rh^3+^ complex in positive ion mode, and (c) Br^–^ in negative ion mode; see ESI† for experimental details.

The ability to access *N*-brominated sulfamate **5** has enabled a series of experiments to determine the role of Rh_2_(oct)_4_ in the oxidation reaction. UV/Visible spectroscopic monitoring of the reaction of **5** with Rh_2_(oct)_4_ in CH_2_Cl_2_ reveals a distinct change in the absorption spectrum, evidenced by the disappearance of the feature at *λ*_max_ = 418 nm, shifting of the *λ*_max_ at 655 to 595 nm, and the appearance of a new *λ*_max_ at 985 nm (Fig. 1a). The final absorption spectrum is indicative of a mixed-valent Rh^2+^/Rh^3+^ tetracarboxylate dimer,^57^,^58^ consistent with a mechanism involving one-electron reduction of the N–Br bond to generate an N-centered radical. Support for this conclusion has been obtained through electrospray ionization mass spectrometric (ESI-MS) analysis, which confirms the presence of both the Rh^2+^/Rh^3+^ complex and free Br^–^ (Fig. 1b–c) resulting from this reaction.

**Scheme 3:**
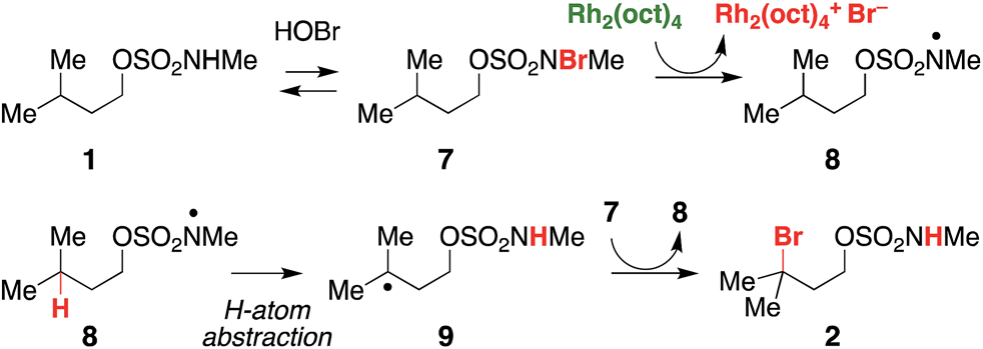
A proposed radical-chain transfer process for C–H bromination.

In a reaction mixture containing **5** and Rh_2_(oct)_4_, the red color ascribed to the mixed-valent dirhodium species persists for several hours. Under standard catalytic reaction conditions, however, the deep green color of intact Rh_2_(oct)_4_ bleaches to pale yellow within 30 min following NaOCl addition. A UV/vis spectrum of the reaction mixture at this time point shows a featureless spectrum, consistent with decomposition of the rhodium dimer (Fig. S4a†). Interestingly, at 30 min, product conversion is only ∼30%, with starting material accounting for the remainder of the mass balance (Fig. S4b†). After the full reaction time (15 h), the isolated product yield is 61%. Thus, the reaction appears to proceed beyond the lifetime of Rh_2_(oct)_4_, suggesting its role as an initiator rather than as a catalyst for oxidative halogenation (Scheme 3). Accordingly, these data have led us to favor a mechanism for C–H bromination through a chain transfer process involving N- and C-centered radical intermediates, as depicted in Scheme 3. We cannot, however, discount the possibility that the intermediate carbon radical could also react with [Rh_2_(oct)_4_Br] to give the brominated product.

**Fig. 2:**
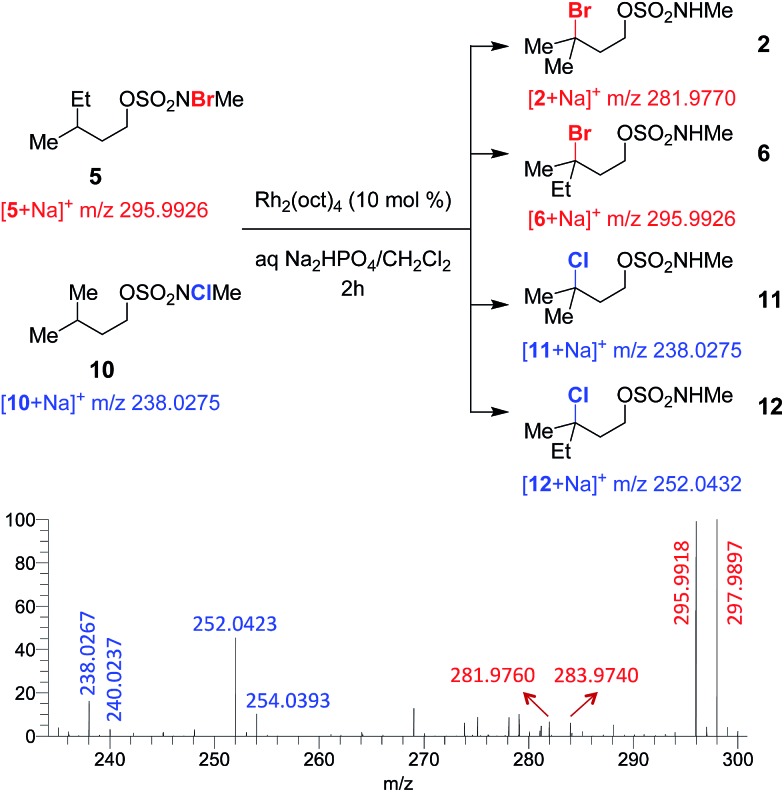
High-resolution ESI-MS analysis shows that cross-halogenated products form in a competition experiment between bromosulfamate **5** and chlorosulfamate **10**. Experimental *m*/*z* values (in the spectrum) agree well with the theoretical *m*/*z* values (underneath the chemical structures).

To test for a radical chain mechanism, a 1 : 1 mixture of brominated sulfamate **5** and chlorinated sulfamate **10** was stirred with catalytic Rh_2_(oct)_4_. ESI-MS analysis of the reaction mixture at 2 h revealed brominated products **2** and **6** and chlorinated products **11** and **12** (Fig. 2 and S5†). Such a product distribution lends strong support to our mechanistic scheme, as only an intermolecular collision between intermediates derived from **5** and **10** could lead to cross-halogenated products **2** and **12**.

**Scheme 4:**
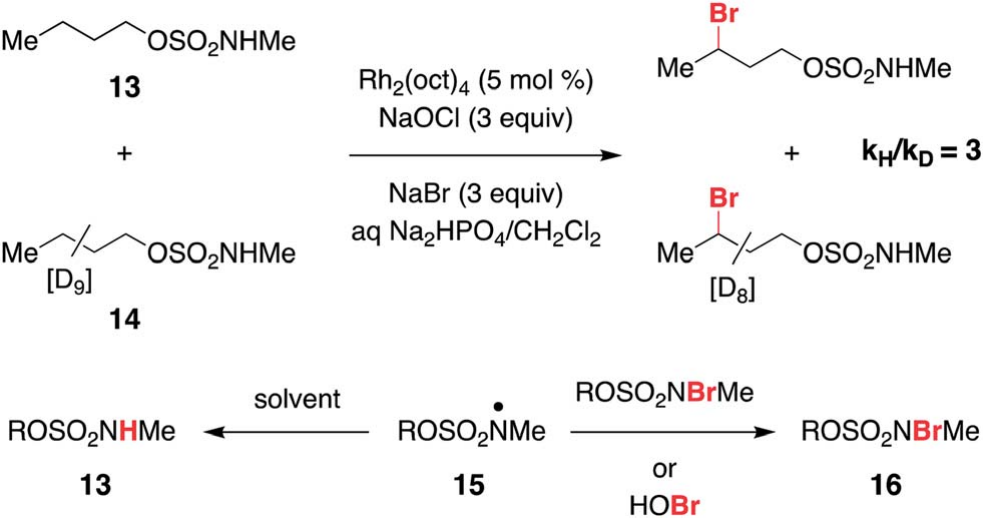
KIE study suggests reversibility of N-centered radical formation.

As a final piece of mechanistic insight, a kinetic isotope effect (KIE) of [*P*_H_]/[*P*_D_] = 3 (Scheme 4 and Fig. S6†) has been measured in a competition experiment between protio- and deutero-sulfamate substrates, **13** and **14**. This result suggests that N-centered radical formation is not a committed, irreversible step. Reactions of **15** with a second *N*-halogenated sulfamate (or HOBr) to form **16** or with solvent to regenerate **13** are possible pathways that apparently compete with intramolecular γ-C–H abstraction, thus giving rise to a non-unitary KIE value in the competition experiment.^59^

**Scheme 5:**
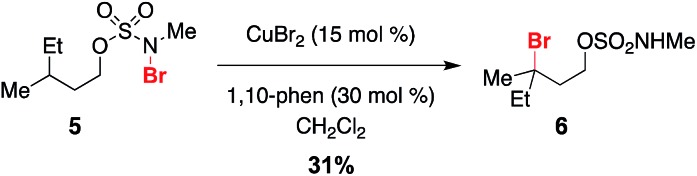
CuBr_2_/phenanthroline as an alternate metal complex for reaction initiation.

Given a radical chain mechanism for C–H halogenation, it is possible that metal complexes other than Rh_2_(oct)_4_ could serve as initiators. We have found that treatment of **5** with a combination of 15 mol% CuBr_2_ and 1,10-phenanthroline forms the tertiary bromide product **6** in 31% yield (Scheme 5 and Fig. S7†).^60^ While the efficiency of this reaction is lower than that with Rh_2_(oct)_4_ (Scheme 2), formation of **6** suggests that, at least in principle, new reaction manifolds utilizing first-row transition metals can be optimized for the oxidative halogenation of sp^3^ C–H bonds.

## Conclusions

A method for site-selective bromination of sp^3^ C–H bonds using *N*-methyl sulfamate substrates is presented. Following halogenation, the sulfamate directing group can be displaced with nucleophiles to generate value-added alkylbromide products. The scope and predictability of this oxidation reaction distinguish these findings. UV/visible spectroscopy, ESI-MS analysis, and substrate probe experiments implicate a radical chain mechanism for C–H halogenation, initiated by Rh_2_(oct)_4_. Further exploration of sulfamate directing groups in C–H functionalization catalysis is warranted and should lead to high-precision methods for modifying sp^3^ carbon centers.

## Conflicts of interest

There are no conflicts to declare.

## Supplementary Material

Supplementary information
