# Supplementary material for: Site-selective bromination of sp3 C–H bonds
Source: Chem Sci. 2017 Nov 20;9(1):100–4. doi: 10.1039/c7sc04611a (PMC5873043; doi:10.1039/c7sc04611a)
Supplement: Supplementary file 1 [file SC-009-C7SC04611A-s001.pdf]

Electronic Supplementary Material (ESI) for Chemical Science.
This journal is © The Royal Society of Chemistry 2017

Supplementary material

Site-Selective Bromination of sp^3 C–H Bonds

Shyam Sathyamoorthi[1], Shibdas Banerjee[1,2], J. Du Bois[1]*, Noah Z. Burns[1]*, Richard N. Zare[1]*

[1]Department of Chemistry, Stanford University, Stanford, CA 94305-5080

[2]Indian Institute of Science Education and Research Tirupati, Karakambadi Road, Tirupati-517507, India.

General. All reagents were obtained commercially unless otherwise noted. Reactions were performed using glassware that was oven-dried. Air- and moisture-sensitive liquids and solutions were transferred via syringe or stainless steel cannula. Organic solutions were concentrated under reduced pressure (~15 Torr) by rotary evaporation. Solvents were purified by passage under 12 psi through activated alumina columns. Chromatography was performed on Silicycle Silia-P Silica Gel (40-63 µm). Compounds purified by chromatography were typically applied to the adsorbent bed using the indicated solvent conditions with a minimum amount of added methylene chloride as needed for solubility. Thin layer chromatography was performed on either Whatman Partisil K6F Silica Gel 60 Å plates (250 µm) or EMD Chemicals Silica Gel (250 µm). Visualization of the developed chromatogram was accomplished by fluorescence quenching and/or by staining with butanolic ninhydrin, aqueous potassium permanganate, aqueous ceric ammonium molybdate (CAM), or ethanolic anisaldehyde.

Continuous UV/Vis reaction monitoring (Figure 2a) was performed on a Varian Cary 50 Scan spectrophotometer with fiber-optic leads to a custom-designed quartz immersion probe (Hellma) of 0.1 cm optical path length in a custom-designed sample cell (ChemGlass). Spectra were recorded in 1.5 min interval until a reaction time of 15 min. After 15 min, spectra were recorded in 15 min intervals over a reaction time of 3 h.

Nuclear magnetic resonance (NMR) spectra were acquired on either a Varian Inova-600 operating at 600 and 150 MHz, a Varian Inova-300 operating at 300 and 75 MHz, a Varian Mercury-400 operating at 400 and 100 MHz, or a Varian Inova-500 operating at 500 and 125 MHz, and are referenced internally according to residual solvent signals. Data for NMR are recorded as follows: chemical shift (δ, ppm), multiplicity (s, singlet; br s, broad singlet; d, doublet; t, triplet; q, quartet; quint, quintet; sext, sextet; m, multiplet), integration, coupling constant (Hz). Data are reported in terms of chemical shift (δ, ppm). Infrared spectra were recorded on either a Thermo-Nicolet IR100 spectrometer or a Thermo-Nicolet IR300 spectrometer as thin films using NaCl salt plates and are reported in frequency of absorption. High-resolution mass spectra were obtained from the Vincent Coates Foundation Mass Spectrometry Laboratory at Stanford University.

Electrospray ionization mass spectrometric (ESI-MS) studies were performed on a high-resolution mass spectrometer (Thermo Scientific LTQ Orbitrap XL Hybrid Ion Trap-Orbitrap mass spectrometer) using a homebuilt ESI source. Nitrogen (120 psi) was used as a sheath gas. Electrospray of the analyte solution was performed in either positive (+5 kV) or negative (-5 kV) ion mode. The heated capillary (MS inlet) temperature and voltage were maintained at 275 °C and 44 V respectively. Helium was used as the collision gas in the collision induced dissociation cell (CID cell; an ion trap). CID spectra (MS/MS) were acquired using an isolation width of 0.9 m/z unit with activation Q and activation time set to 0.25 and 30 ms respectively. All experiments were carried out under identical conditions, unless otherwise stated. The ion optics were tuned to get maximum ion count. Data acquisition was performed using XCalibur software (Thermo Fisher Scientific)

A. Preparation of methylsulfamoyl chloride

$MeHNSO_2Cl$. A 10 mL reaction flask was charged with methylsulfamic acid (300 mg, 2.70 mmol), PCl_5 (618 mg, 2.97 mmol, 1.1 equiv) and 5 mL of toluene. The mixture was stirred at 80 °C for 5 h. After cooling to room temperature, the reaction contents were filtered through a small plug of cotton. The cotton plug was rinsed with an additional 10 mL of CH_2Cl_2, and the combined filtrates were concentrated under reduced pressure to a light brown oil (304 mg, 87%). This material was used immediately and without further purification in the subsequent sulfamoylation reaction.

B. General procedure for preparation of methylsulfamate substrates

To a mixture of alcohol starting material (2.1 mmol) in 5 mL of CH_2Cl_2 was added methylsulfamoyl chloride (304 mg, 2.35 mmol, 1.1 equiv). To the resulting light yellow solution was added dropwise Et_3N (330 μL, 2.35 mmol, 1.1 equiv). After stirring for 2 h, the reaction was quenched by slow addition of 10 mL of 1.0 M aqueous HCl. The reaction contents were transferred to a 60 mL separatory funnel with 10 mL of CH_2Cl_2. The organic layer was collected and the aqueous fraction was extracted with 1 x 10 mL of CH_2Cl_2 and 1 x 10 mL of EtOAc. The combined organic fractions were dried over Na_2SO_4, filtered, and concentrated under reduced pressure. Pure material was obtained following chromatography on silica gel or by reversed phase HPLC (conditions given below).

C. General procedure for oxidative bromination

To a mixture of methylsulfamate starting material (0.125 mmol) in 1.2 mL of CH_2Cl_2 was added $Rh_2(oct)_4$ (5 mg, 6.0 μmol, 0.05 equiv) and NaBr (39 mg, 0.375 mmol, 3.0 equiv). To the resulting cloudy green mixture was added 1.2 mL of saturated aqueous Na_2HPO_4 followed by aqueous NaOCl (10-15% active chlorine, 0.230 mL, 0.375 mmol, 3.0 equiv). After stirring for 15 h, the reaction was quenched by the addition of 10 mL of saturated aqueous $Na_2S_2O_3$. The reaction contents were transferred to a 60 mL separatory funnel with 10 mL of CH_2Cl_2. The organic layer was collected and the aqueous fraction was extracted with 1 x 10 mL of CH_2Cl_2 and 1 X 10 mL of EtOAc. The combined organic fractions were dried over Na_2SO_4, filtered, and concentrated under reduced pressure. Pure material was obtained following chromatography on silica gel or by reversed phase HPLC (conditions given below).

D. General procedure for oxidative chlorination

To a mixture of methylsulfamate starting material (0.125 mmol) in 1.2 mL of CH_2Cl_2 was added $Rh_2(oct)_4$ (5 mg, 6.0 μmol, 0.05 equiv). To the resulting cloudy green mixture was added 1.2 mL of saturated aqueous Na_2HPO_4 followed by aqueous NaOCl (10-15% active chlorine, 0.230 mL, 0.375 mmol, 3 equiv). After stirring for 15 h, the reaction was quenched by the addition of 10 mL of saturated aqueous $Na_2S_2O_3$. The reaction contents were transferred to a 60 mL separatory funnel with 10 mL of CH_2Cl_2. The organic layer was collected and the aqueous fraction was extracted with 1 x 10 mL of CH_2Cl_2 and 1 x 10 mL of EtOAc. The combined organic layers were dried over Na_2SO_4, filtered, and concentrated under reduced pressure. Pure material was obtained following chromatography on silica gel or by reversed phase HPLC (conditions given below).

Characterization data for sulfamate substrates

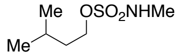

Table 2, Entry 1: Substrate synthesized according to general procedure B; purified by chromatography on silica gel (100:1 CH_2Cl_2/acetone); clear oil (90%): TLC R_f = 0.33 (11:3 hexanes/EtOAc); ^{1}H NMR ($CDCl_3$, 400 MHz) δ 4.33-4.27 (br m, 1H), 4.17 (t, 2H, J = 6.6 Hz), 2.81 (d, 3H, J = 4.0 Hz), 1.77 (sept, 1H, J = 8.0 Hz), 1.65-1.60 (m, 2H), 0.94 (d, 6H, J = 8.0 Hz) ppm; ^{13}C NMR ($CDCl_3$, 75 MHz) δ 69.6, 37.7, 29.9, 24.9, 22.5 ppm; IR ν 2960, 1470, 1176, 950 cm^{-1}; HRMS calcd for $C_6H_{15}NO_3SNa^+$ 204.0665 found 204.0661 (MNa^+).

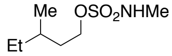

Table 2, Entry 1: Substrate synthesized according to general procedure B; purified by chromatography on silica gel (50:1 CH_2Cl_2/EtOAc); clear oil (87%): TLC R_f = 0.35 (11:3 hexanes/EtOAc); ^{1}H NMR ($CDCl_3$, 400 MHz) δ 4.54-4.49 (br m, 1H), 4.21-4.10 (m, 2H), 2.80 (d, 3H, J = 4.0 Hz), 1.82-1.72 (m, 1H), 1.54-1.47 (m, 2H), 1.44-1.31 (m, 1H), 1.27-1.13 (m, 1H), 0.91-0.84 (m, 6H) ppm; ^{13}C NMR ($CDCl_3$, 100 MHz) δ 69.4, 35.3, 30.9, 29.8, 29.2, 18.8, 11.2 ppm; IR ν 2962, 1344, 1174, 947 cm^{-1}; HRMS calcd for $C_7H_{17}NO_3SNa^+$ 218.0821 found 218.0818 (MNa^+).

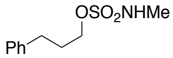

Table 2, Entry 2: Substrate synthesized according to general procedure B; purified by chromatography on silica gel (100:1 CH_2Cl_2/acetone); light yellow solid (59%): TLC R_f = 0.21 (11:3 hexanes/EtOAc); ^{1}H NMR ($CDCl_3$, 400 MHz) δ 7.36-7.26 (m, 5H), 4.35-4.30 (br m, 1H), 4.18 (t, 2H, J = 8.0 Hz), 2.84 (d, 3H, J = 8.0 Hz), 2.78 (t, 2H, J = 8.0 Hz), 2.14-2.05 (m, 2H) ppm; ^{13}C NMR ($CDCl_3$, 75 MHz) δ 140.8, 128.8, 128.7, 126.5, 70.2, 31.9, 30.7, 30.1 ppm; IR ν 3320, 2949, 1497, 1079 cm^{-1}; HRMS calcd for $C_{10}H_{16}NO_3S^+$ 230.0845 found 230.0848 (MH^+).

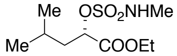

Table 2, Entry 3: Substrate synthesized according to general procedure B; purified by reversed-phase HPLC (Alltima C18, 10 μm, 22 x 250 mm, eluting with gradient flow over 65 min of 0:100 →100:0 MeCN/H_2O, retention time of 40-42 min at a flow rate of 12 mL/min); clear oil (45%): TLC R_f = 0.28 (20:3 hexanes/EtOAc); ^{1}H NMR ($CDCl_3$, 400 MHz) δ 4.90 (dd, 1H, J = 8.0, 4.0 Hz), 4.58-4.54 (br m, 1H), 4.24 (q, 2H, J = 8.0 Hz), 2.86 (d, 3H, J = 4.0 Hz), 1.87-1.80 (m, 2H), 1.68-1.61 (m, 1H), 1.31 (t, 3H, J = 8.0 Hz), 0.98 (d, 3H, J = 8.0 Hz), 0.97 (d, 3H, J = 8.0 Hz) ppm; ^{13}C NMR ($CDCl_3$, 125 MHz) δ 170.6, 77.2, 62.1, 40.9, 30.2, 24.2, 23.2, 21.5, 14.3 ppm; IR ν 3317, 2962, 1743, 1352, 1024 cm^{-1}; HRMS calcd for $C_9H_{20}NO_5S^+$ 254.1057 found 254.1060 (MH^+).

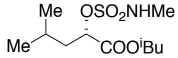

Table 2, Entry 3: Substrate synthesized according to general procedure B; purified by reversed-phase HPLC (Alltima C18, 10 μm, 22 x 250 mm, eluting with gradient flow over 65 min of 0:100 →100:0 MeCN/H_2O, retention time of 45-47 min at a flow rate of 12 mL/min); white solid (53%): TLC R_f = 0.22 (5:1 hexanes/EtOAc); ^{1}H NMR ($CDCl_3$, 600 MHz) δ 4.91 (dd, 1H, J = 12.0,

6.0 Hz), 4.63-4.60 (br m, 1H), 3.98-3.93 (m, 2H), 2.86 (d, 3H, J = 6.0 Hz), 2.00-1.93 (m, 1H), 1.88-1.81 (m, 2H), 1.67-1.62 (m, 1H), 0.99-0.94 (m, 12H) ppm; ^{13}C NMR ($CDCl_3$, 125 MHz) δ 170.6, 77.5, 72.0, 41.0. 30.3, 27.9, 24.5, 23.1, 21.6, 19.2 ppm; IR ν 3315, 2962, 1741, 1354, 1005 cm^{-1}; HRMS calcd for $C_{11}H_{24}NO_5S^+$ 282.1370 found 282.1367 (MH^+).

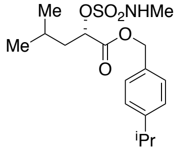

Table 2, Entry 3: Substrate synthesized according to general procedure B; purified by chromatography on silica gel (gradient elution: 20:1→1:1 hexanes/EtOAc); light yellow solid (39%): TLC R_f = 0.44 (10:3 hexanes/EtOAc); ^{1}H NMR ($CDCl_3$, 400 MHz) δ 7.29 (d, 2H, J = 8.0 Hz), 7.24 (d, 2H, J = 8.0 Hz), 5.17 (s, 2H), 4.94-4.91 (m, 1H), 4.77-4.76 (m, 1H), 2.92 (sept, 1H, J = 8.0 Hz), 2.73 (d, 3H, J = 4.0 Hz), 1.87-1.81 (m, 2H), 1.67-1.61 (m, 1H), 1.25 (d, 6H, J = 8.0 Hz), 0.96-0.93 (m, 6H) ppm; ^{13}C NMR ($CDCl_3$, 100 MHz) δ 170.1, 149.6, 132.3, 128.6, 126.7, 77.1, 67.5, 40.6, 33.9, 29.9, 24.2, 23.9, 22.9, 21.3 ppm; IR ν 3298, 2959, 1738, 1362, 1008 cm^{-1}; HRMS calcd for $C_{17}H_{28}NO_5S^+$ 358.1683 found 358.1688 (MH^+).

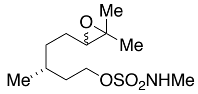

Table 2, Entry 4: Substrate synthesized according to general procedure B; a portion was purified by reversed-phase HPLC (Alltima C18, 10 μm, 22 x 250 mm, eluting with gradient flow over 65 min of 0:100 →100:0 $MeCN/H_2O$, retention time of 37-38 min at a flow rate of 12 mL/min); light yellow oil; TLC R_f = 0.27 (5:2 hexanes/EtOAc); ^{1}H NMR ($CDCl_3$, 600 MHz) δ 4.62-4.57 (br m, 1H), 4.21-4.14 (m, 2H), 2.79 (d, 3H, J = 6.0 Hz), 2.70-2.68 (m, 1H), 1.82-1.75 (m, 1H), 1.71-1.64 (m, 1H), 1.61-1.50 (m, 3H), 1.49-1.40 (m, 1H), 1.39-1.35 (m, 1H), 1.30 (s, 3H), 1.26 (s, 3H), 0.94 (d, 3H, J = 12.0 Hz) ppm; ^{13}C NMR ($CDCl_3$, 100 MHz) δ 69.0, 68.9, 64.6, 64.5, 58.6, 58.4, 35.6, 35.4, 33.4, 33.3, 29.8, 29.3, 29.2, 26.3, 26.0, 24.9, 19.2, 19.1, 18.7, 18.6 ppm; IR ν 2929, 1461, 1177 cm^{-1}; HRMS calcd for $C_{11}H_{24}NO_4S^+$ 266.1421 found 266.1425 (MH^+).

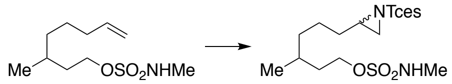

Table 2, Entry 5: To a solution of $Cl_3CCH_2SO_3NH_2$ (120 mg, 0.53 mmol, 1.2 equiv) in 2.0 mL of CH_2Cl_2 were added sequentially olefin (100 mg, 0.43 mmol), MgO (30 mg, 0.74 mmol, 1.7 equiv), and Rh_2(tfacam)$_4$ (10 mg, 15.0 μmol, 0.04 equiv). The resulting purple mixture was cooled to 0 °C and PhI(OAc)$_2$ (180 mg, 0.56 mmol, 1.3 equiv) was added. The suspension quickly turned orange after the addition of PhI(OAc)$_2$ and was allowed to warm slowly to room temperature over 2 h. After 6 h of stirring, during which time the solution color faded to pale yellow, the reaction was diluted with 10 mL of CH_2Cl_2 and filtered through Celite. The flask and filter cake were washed thoroughly with CH_2Cl_2 and the combined filtrates were concentrated under reduced pressure. The isolated material was purified by chromatography on silica gel (gradient elution: 4:1→1:1 hexanes/EtOAc) to afford the desired aziridine as a clear oil (82 mg, 42%); TLC R_f = 0.36 (10:3 hexanes/EtOAc); ^{1}H NMR ($CDCl_3$, 400 MHz) δ 4.83 (d, 1H, J = 12.0 Hz), 4.79 (d, 1H, J = 12.0 Hz), 4.53-4.46 (br m, 1H), 4.21-4.07 (m, 2H), 2.90-2.85 (m, 1H), 2.79 (d, 3H, J = 4.0 Hz),

2.73 (d, 1H, J = 4.0 Hz), 2.27 (d, 1H, J = 4.0 Hz), 1.81-1.72 (m, 1H), 1.62-1.35 (m, 7H), 1.28-1.18 (m, 1H), 0.93 (d, 3H, J = 8.0 Hz) ppm; ^{13}C NMR ($CDCl_3$, 100 MHz) δ 93.2, 79.7, 69.2, 42.4, 36.2, 36.1, 35.8, 35.7, 31.5, 30.1, 29.5, 29.4, 24.2, 24.1, 19.4, 19.3 ppm; IR 3000, 1363, 1179, 949 cm^{-1}; HRMS calcd for $C_{12}H_{23}Cl_3N_2O_6S_2Na^+$ 482.9955 found 482.9966 (MNa^+).

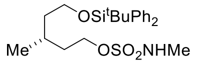

Table 2, Entry 6: Substrate synthesized according to general procedure B; a portion of material was purified by purified by reversed-phase HPLC (Alltima C18, 10 µm, 22 x 250 mm, eluting with gradient flow over 65 min of 0:100 →100:0 MeCN/H_2O, retention time of 60-61 min at a flow rate of 12 mL/min); clear oil; TLC R_f = 0.22 (3:1 hexanes/EtOAc); ^1H NMR ($CDCl_3$, 300 MHz) δ 7.71-7.68 (m, 4H), 7.49-7.39 (m, 6H), 4.39-4.38 (br m, 1H), 4.21-4.17 (m, 2H), 3.77-3.69 (m, 2H), 2.79 (d, 3H, J = 6.0 Hz), 1.91-1.74 (m, 2H), 1.70-1.38 (m, 3H), 0.91 (s, 9H), 0.92 (d, 3H, J = 6.0 Hz) ppm; ^{13}C NMR ($CDCl_3$, 125 MHz) δ 135.8, 134.1, 129.9, 127.9, 69.4, 61.9, 39.5, 36.0, 30.1, 27.1, 26.5, 19.6, 19.5 ppm; IR ν 2931, 1347, 1111, 951 cm^{-1}; HRMS calcd for $C_{23}H_{36}NO_4SSi^+$ 450.2129 found 450.2125 (MH^+).

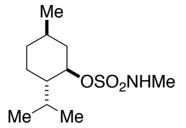

Table 2, Entry 7: Substrate synthesized according to general procedure B; purified by reversed-phase HPLC (Alltima C18, 10 µm, 22 x 250 mm, eluting with gradient flow over 65 min of 0:100 →100:0 MeCN/H_2O, retention time of 49-50 min at a flow rate of 12 mL/min); white solid (32%); TLC R_f = 0.5 (10:3 hexanes/EtOAc); ^1H NMR ($CDCl_3$, 500 MHz) δ 4.52-4.51 (m, 1H), 4.41-4.36 (m, 1H), 2.83 (d, 3H, J = 5.0 Hz), 2.35-2.32 (m, 1H), 2.19-2.12 (m, 1H), 1.74-1.68 (m, 2H), 1.52-1.40 (m, 2H), 1.26-1.18 (m, 1H), 1.10-1.03 (m, 1H), 0.96-0.84 (m, 10H) ppm; ^{13}C NMR ($CDCl_3$, 125 MHz) δ 83.9, 47.9, 41.7, 34.1, 31.9, 30.2, 25.8, 23.2, 22.2, 21.2, 15.8 ppm; IR ν 3313, 2817, 1456, 1175 cm^{-1}; HRMS calcd for $C_{11}H_{23}NO_3SNa^+$ 272.1291 found 272.1288 (MNa^+).

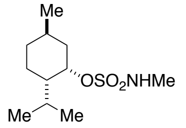

Substrate synthesized according to general procedure B; a portion of material was purified by reversed-phase HPLC (Alltima C18, 10 µm, 22 x 250 mm, eluting with gradient flow over 65 min of 0:100 →100:0 MeCN/H_2O, retention time of 44-46 min at a flow rate of 12 mL/min); clear oil; TLC R_f = 0.5 (5:1 hexanes/EtOAc); ^1H NMR ($CDCl_3$, 500 MHz) δ 5.00-4.98 (br m, 1H), 4.56-4.50 (br m, 1H), 2.82-2.81 (d, 3H, J = 5.0 Hz), 2.33-2.29 (m, 1H), 1.84-1.71 (m, 3H), 1.67-1.59 (m, 1H), 1.38-1.29 (m, 1H), 1.12-1.06 (m, 1H), 1.03-0.88 (m, 11H) ppm; ^{13}C NMR ($CDCl_3$, 125 MHz) δ 81.6, 48.1, 39.7, 34.8, 30.1, 28.9, 26.3, 24.5, 22.3, 21.1, 21.0 ppm; IR ν 3316, 2950, 1339, 1174 cm^{-1}; HRMS calcd for $C_{11}H_{23}NO_3SNa^+$ 272.1291 found 272.1295 (MNa^+).

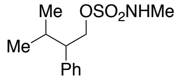

Table 2, Entry 8: Substrate synthesized according to general procedure B; purified by chromatography on silica gel (100:1 CH_2Cl_2/acetone); white solid (80%); TLC R_f = 0.29 (5:1 hexanes/EtOAc); ^1H NMR ($CDCl_3$, 300 MHz) δ 7.37-7.19 (m, 5H), 4.48-4.36 (m, 2H), 4.10-4.03 (br m, 1H), 2.78-2.71 (m, 1H), 2.50 (d, 3H, J = 6.0 Hz), 2.11-1.99 (m, 1H), 1.05 (d, 3H, J = 6.0 Hz), 0.80 (d, 3H, J = 6.0 Hz) ppm; ^{13}C NMR ($CDCl_3$, 75 MHz) δ 140.7, 128.8, 128.6, 127.1, 72.9, 52.1, 30.1, 29.7, 21.1, 20.7 ppm; IR ν 3318, 2962, 1454, 969 cm^{-1}; HRMS calcd for $C_{12}H_{20}NO_3S^+$ 258.1158 found 258.1157 (MH^+).

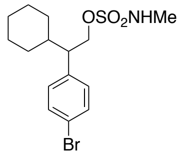

Table 2, Entry 9: Substrate synthesized according to general procedure B; purified by chromatography on silica gel (gradient elution: 10:1→5:1 hexanes/EtOAc); white solid (49%); TLC R_f = 0.38 (10:3 hexanes/EtOAc); ^1H NMR ($CDCl_3$, 600 MHz) δ 7.42 (d, 2H, J = 6.0 Hz), 7.04 (d, 2H, J = 6.0 Hz), 4.37 (dd, 1H, J = 6.0, 6.0 Hz), 4.33-4.28 (m, 2H), 2.76-2.71 (m, 1H), 2.54 (d, 3H, J = 6.0 Hz), 1.87-1.85 (m, 1H), 1.76-1.73 (m, 1H), 1.67-1.61 (m, 3H), 1.43-1.41 (m, 1H), 1.28-1.21 (m, 1H), 1.15-1.07 (m, 2H), 1.03-0.96 (m, 1H), 0.82-0.77 (m, 1H) ppm; ^{13}C NMR ($CDCl_3$, 100 MHz) δ 139.6, 131.4, 130.2, 120.6, 71.7, 50.4, 39.4, 31.0, 30.7, 29.6, 26.18, 26.15, 26.1 ppm; IR ν 2926, 1488, 1348, 969 cm^{-1}; HRMS calcd for $C_{15}H_{22}BrNO_3SNa^+$ 398.0396 found 398.0392 (MNa^+).

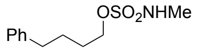

Table 2, Entry 10: Substrate synthesized according to general procedure B; purified by chromatography on silica gel (100:1 CH_2Cl_2/acetone); clear oil (23%); TLC R_f = 0.39 (10:3 hexanes/EtOAc); ^1H NMR ($CDCl_3$, 400 MHz) δ 7.35-7.16 (m, 5H), 4.29-4.27 (br m, 1H), 4.21-4.12 (m, 2H), 2.79 (d, 3H, J = 8.0 Hz), 2.68-2.64 (m, 2H), 1.82-1.68 (m, 4H) ppm; ^{13}C NMR ($CDCl_3$, 125 MHz) δ 141.9, 128.7, 126.2, 70.9, 35.5, 30.0, 28.6, 27.6 ppm; IR ν 3319, 2943, 1412, 1175, 1081 cm^{-1}; HRMS calcd for $C_{11}H_{17}NO_3SNa^+$ 266.0821 found 266.0817 (MNa^+).

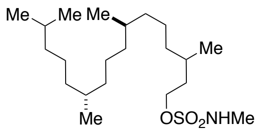

Table 2, Entry 11: Substrate synthesized according to general procedure B; a portion of material was purified by reversed-phase HPLC (Alltima C18, 10 μm, 22 x 250 mm, eluting with gradient flow over 65 min of 0:100 →100:0 $MeCN/H_2O$); clear oil; TLC R_f = 0.33 (20:3 hexanes/EtOAc); ^1H NMR ($CDCl_3$, 600 MHz) δ 4.26-4.22 (br m, 1H), 4.21-4.14 (m, 2H), 2.82 (d, 3H, J = 6.0 Hz), 1.80-1.02 (m, 24H), 0.92 (d, 3H, J = 6.0 Hz), 0.87-0.84 (m, 12H) ppm; ^{13}C NMR ($CDCl_3$, 100 MHz) δ 69.3, 39.3, 37.6, 37.4, 37.34, 37.26, 37.23, 37.21, 37.14, 37.12, 35.8, 35.7, 32.8, 29.7, 29.4, 27.9, 24.8, 24.4, 24.2, 22.7, 22.6, 19.7, 19.6, 19.3, 19.2 ppm; IR ν 3317, 2868, 1433, 1349, 1178 cm^{-1}; HRMS calcd for $C_{21}H_{45}NO_3SNa^+$ 414.3012 found 414.3019 (MNa^+).

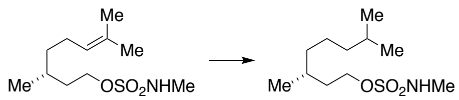

Table 2, Entry 12: A solution of olefin (255 mg, 1.01 mmol) in 10 mL of MeOH was charged with 5% Pd/C (212 mg, 10 mol%). The suspension was sparged with gentle stream of H_2 gas for 15 min. The flask was then fitted with a balloon of H_2 and the contents were stirred for 12 h. Following this time, the mixture was sparged with N_2 gas for 15 min, diluted with 30 mL of MeOH, and filtered through a pad of Celite. The flask and filter cake were rinsed with 30 mL of EtOAc, and the combined filtrates were concentrated under reduced pressure to a clear oil (228 mg, 90%). The product was determined by ^{1}H NMR to be sufficiently pure for use in the subsequent reaction; TLC R_f = 0.28 (5:1 hexanes/EtOAc); ^{1}H NMR ($CDCl_3$, 400 MHz) δ 4.27-4.13 (m, 3H), 2.82 (d, 3H, J = 4.0 Hz), 1.82-1.73 (m, 1H), 1.65-1.46 (m, 3H), 1.36-1.10 (m, 6H), 0.91 (d, 3H, J = 4.0 Hz), 0.87 (d, 6H, J = 4.0 Hz) ppm; ^{13}C NMR ($CDCl_3$, 100 MHz) δ 69.6, 39.4, 37.3, 36.0, 30.1, 29.6, 28.2, 24.8, 22.9, 22.8, 19.5 ppm; IR ν 3316, 2927, 1345, 1175, 1080 cm^{-1}; HRMS calcd for $C_{11}H_{25}NO_3SNa^+$ 274.1447 found 274.1445 (MNa^+).

Characterization data for halogenated products

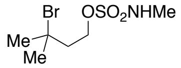

Table 2, Entry 1: Synthesized according to general procedure C; purified by chromatography on silica gel (20:3 hexanes/EtOAc); clear oil (20 mg, 61%); TLC R_f = 0.14 (5:1 hexanes/EtOAc); ^{1}H NMR ($CDCl_3$, 500 MHz) δ 4.49-4.45 (m, 1H), 4.42 (t, 2H, J = 6.9 Hz), 2.85 (d, 3H, J = 5.0 Hz), 2.28 (t, 2H, J = 6.90 Hz), 1.84 (s, 6H) ppm; ^{13}C NMR δ 69.0, 63.8, 45.5, 34.9, 30.1 ppm; IR ν 3318, 2962, 1414, 1341 cm^{-1}; HRMS calcd for $C_6H_{14}BrNO_3SNa^+$ 281.9770 found 281.9765 (MNa^+).

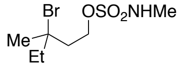

Table 2, Entry 1: Synthesized according to general procedure C; purified by chromatography on silica gel (20:3 hexanes/EtOAc); clear oil (26 mg, 75%); TLC R_f= 0.19 (5:1 hexanes/EtOAc); ^{1}H NMR ($CDCl_3$, 400 MHz) δ 4.42-4.38 (m, 3H), 2.83 (d, 3H, J = 4.0 Hz), 2.36-2.29 (m, 1H), 2.23-2.16 (m, 1H), 1.99-1.79 (m, 2H), 1.74 (s, 3H), 1.06 (t, 3H, J = 8.0 Hz) ppm; ^{13}C NMR ($CDCl_3$, 100 MHz) δ 70.0, 68.9, 43.3, 39.1, 31.3, 30.1, 10.4 ppm; IR ν 3316, 2921, 1995, 1618, 1175 cm^{-1}; HRMS calcd for $C_7H_{16}BrNO_3SNa^+$ 295.9926 found 295.9926 (MNa^+).

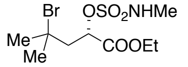

Table 2, Entry 3: Substrate synthesized according to general procedure C; purified by chromatography on silica gel (3:1 hexanes/EtOAc); white solid (29 mg, 70%); TLC R_f = 0.16 (20:3 hexanes/EtOAc); ^{1}H NMR ($CDCl_3$, 500 MHz) δ 5.23 (dd, 1H, J = 10.0, 5.0 Hz), 4.71-4.66 (m, 1H), 4.34-4.24 (m, 2H), 2.92 (d, 3H, J = 5.0 Hz), 2.54 (dd, 1H, J = 15.8, 2.4 Hz), 2.34 (dd, 1H, J = 15.0, 5.0 Hz), 1.90 (s, 3H), 1.89 (s, 3H), 1.35 (t, 3H, J = 5.0 Hz) ppm; ^{13}C NMR ($CDCl_3$, 125 MHz) δ 170.0, 77.9, 63.6, 62.6, 48.5, 35.8, 33.7, 30.5, 14.3 ppm; IR ν 3319, 2983, 1745, 1351, 900 cm^{-1}; HRMS calcd for $C_9H_{19}BrNO_5S^+$ 322.0162 found 322.0172 (MH^+).

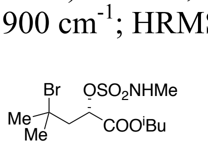

Table 2, Entry 3: Substrate synthesized according to general procedure C; purified by chromatography on silica gel (10:1 hexanes/EtOAc); white solid (33 mg, 76%); TLC R_f = 0.27 (5:1 hexanes/EtOAc); ^{1}H NMR ($CDCl_3$, 400 MHz) δ 5.22 (dd, 1H, J = 8.0, 2.4 Hz), 4.79-4.68 (br m, 1H), 4.03-3.93 (m, 2H), 2.87 (d, 3H, J = 4.0 Hz), 2.51 (dd, 1H, J = 15.2, 1.4 Hz), 2.31 (dd, 1H, J = 12.0, 8.0 Hz), 2.06-1.93 (m, 1H), 1.87 (s, 3H), 1.86 (s, 3H), 0.96 (d, 6H, J = 8.0 Hz) ppm; ^{13}C NMR ($CDCl_3$, 100 MHz) δ 169.8, 77.6, 72.2, 63.4, 48.4, 35.6, 33.3, 30.2, 27.7, 18.9 ppm; IR ν 3319, 2983, 1745, 1351, 900 cm^{-1}; HRMS calcd for $C_{11}H_{22}BrNO_5SNa^+$ 382.0294 found 382.0289 (MNa^+).

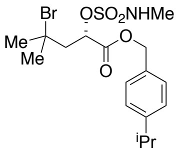

Table 2, Entry 3: Substrate synthesized according to general procedure C; purified by chromatography on silica gel (gradient elution: 10:1→4:1 hexanes/EtOAc); white solid (35 mg, 64%); TLC R_f = 0.41 (10:3 hexanes/EtOAc); ^{1}H NMR ($CDCl_3$, 500 MHz) δ 7.34 (d, 2H, J = 5.0 Hz), 7.28 (d, 2H, J = 5.0 Hz), 5.28-5.19 (m, 3H), 4.55-4.48 (br m, 1H), 2.93 (sept, 1H, J = 6.9 Hz), 2.75 (d, 3H, J = 5.0 Hz), 2.55 (dd, 1H, J = 15.0, 5.0 Hz), 2.34 (dd, 1H, J = 15.0, 5.0 Hz), 1.88 (s, 3H), 1.87 (s, 3H), 1.27 (d, 6H, J = 5.0 Hz) ppm; ^{13}C NMR ($CDCl_3$, 125 MHz) δ 169.8, 150.0, 132.2, 128.9, 127.1, 77.8, 68.2, 63.5, 48.4, 35.8, 34.2, 33.7, 30.3, 24.2 ppm; IR ν 3322, 2961, 1750, 1352, 1033 cm^{-1}; HRMS calcd for $C_{17}H_{26}BrNO_5SNa^+$ 458.0613 found 458.0617 (MNa^+).

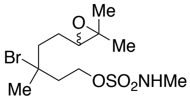

Table 2, Entry 4: Synthesized according to general procedure C; purified by chromatography on silica gel (3:1 hexanes/EtOAc); light yellow oil (31 mg, 71%); TLC R_f = 0.29 (10:3 pentane/EtOAc); ^{1}H NMR ($CDCl_3$, 500 MHz) δ 4.80-4.75 (m, 2H), 4.46-4.42 (m, 2H), 4.40-4.37 (m, 2H), 2.86 (d, 3H, J = 5.0 Hz), 2.85 (d, 3H, J = 5.0 Hz), 2.79-2.76 (m, 2H), 2.40-1.65 (m, 18 H), 1.35 (s, 6H), 1.33 (s, 6H) ppm; ^{13}C NMR ($CDCl_3$, 125 MHz) δ 68.5, 68.32, 68.29, 68.2, 64.1, 59.1, 43.8, 43.3, 42.8, 42.7, 32.2, 31.7, 30.2, 25.9, 25.8, 25.1, 19.02, 18.99 ppm; IR ν 2972, 1349, 1176, 974 cm^{-1}; HRMS (ES^+) calcd for $C_{11}H_{23}BrNO_4S^+$ 344.0526 found 344.0523 (MH^+).

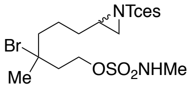

Table 2, Entry 5: Synthesized according to general procedure C; purified by chromatography on silica gel (gradient elution: 10:1→1:1 hexanes/EtOAc); clear oil (51 mg, 75%); TLC R_f = 0.3 (10:3 hexanes/EtOAc); ^{1}H NMR ($CDCl_3$, 400 MHz) δ 4.86-4.80 (m, 2H), 4.50-4.44 (m, 1H), 4.40-4.36 (m, 2H), 2.93-2.87 (m, 1H), 2.83 (d, 3H, J = 4.0 Hz), 2.76-2.74 (m, 1H), 2.37-2.21 (m, 3H), 2.01-1.84 (m, 2H), 1.80-1.68 (m, 6H), 1.55-1.45 (m, 1H) ppm; ^{13}C NMR ($CDCl_3$, 125 MHz) δ 93.2, 79.7, 68.6, 68.4, 45.2, 43.7, 43.6, 42.1, 42.0, 35.75, 35.72, 32.0, 31.9, 31.2, 30.1, 23.2, 23.2 ppm; IR ν 3321, 1418, 1362 cm^{-1}; HRMS (ES^+) calcd for $C_{12}H_{23}BrCl_3N_2O_6S_2^+$ 538.9241 found 538.9218 (MH^+).

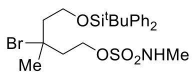

Table 2, Entry 6: Synthesized according to general procedure C; purified by reversed-phase HPLC (Alltima C18, 10 μm, 22 x 250 mm, eluting with gradient flow over 65 min of 0:100→100:0 MeCN/H_2O, retention time of 61 min at a flow rate of 12 mL/min); clear oil (40 mg, 61%); TLC R_f = 0.28 (3:1 hexanes/EtOAc); ^{1}H NMR ($CDCl_3$, 300 MHz) δ 7.75-7.64 (m, 4H), 7.52-7.40 (m, 6H), 4.39 (t, 2H, J = 6.0 Hz), 4.32-4.27 (m, 1H), 3.91 (t, 2H, J = 6.0 Hz), 2.82 (d, 3H, J = 6.0 Hz), 2.44-2.11 (m, 4H), 1.80 (s, 3H), 1.07 (s, 9H) ppm; ^{13}C NMR ($CDCl_3$, 75 MHz) δ 135.8, 133.6, 130.1, 127.9, 68.8, 67.2, 61.8, 48.1, 44.2, 32.5, 30.1, 27.1, 19.4 ppm; IR ν 2587, 1472, 1177, 938 cm^{-1}; HRMS calcd for $C_{23}H_{34}BrNO_4SSiNa^+$ 550.1053 found 550.1065 (MNa^+).

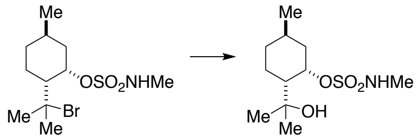

Following general procedure C, (1*S*,2*R*,5*R*)-2-(2-bromopropan-2-yl)-5-methylcyclohexyl methylsulfamate was obtained as an impure yellow oil. This material was dissolved in a 0.9 mL of a 1:1:1 mixture of H_2O/acetone/THF to which Ag_2CO_3 (52 mg, 0.189 mmol, ~1.5 equiv) was then added. After stirring the suspension for 1 h, the reaction mixture was filtered through a small plug of Celite. The flaks and filter cake were rinsed thoroughly with EtOAc and the combined filtrates were concentrated under reduced pressure to a yellow oil. Purification of this material by chromatography on silica gel (gradient elution: 10:1→5:1 hexanes/EtOAc) afforded the alcohol product as a clear oil; TLC R_f = 0.30 (1:1 hexanes/EtOAc); ^{1}H NMR ($CDCl_3$, 500 MHz) δ 5.23-5.21 (m, 1H), 4.73-4.66 (m, 1H), 2.86 (d, 3H, J = 5.0 Hz), 2.27-2.22 (m, 1H), 1.87-1.80 (m, 2H), 1.70-1.56 (m, 2H), 1.48-1.43 (m, 1H), 1.31-1.28 (m, 6H), 1.18-1.12 (m, 1H), 1.06-0.97 (m, 1H), 0.94 (d, 3H, J = 5.0 Hz) ppm; ^{13}C NMR ($CDCl_3$, 125 MHz) δ 80.6, 72.3, 51.2, 40.1, 34.8, 30.1, 29.5, 27.3, 26.6, 22.5, 22.2 ppm; IR ν 3312, 2949, 1457, 1337, 1174 cm^{-1}; HRMS calcd for $C_{11}H_{24}NO_4S^+$ 266.1421 found 266.1426 (MH^+).

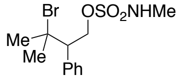

Table 2, Entry 8: Synthesized according to general procedure C; purified by chromatography on silica gel (3:1 hexanes/EtOAc); clear oil (27 mg, 65%); TLC R_f = 0.15 (5:1 hexanes/EtOAc); ^{1}H NMR ($CDCl_3$, 500 MHz) δ 7.43-7.34 (m, 5H), 4.87 (dd, 1H, J = 10.0, 5.0 Hz), 4.69-4.65 (m, 1H), 4.17-4.12 (m, 1H), 3.26 (dd, 1H, J = 10.0, 4.1 Hz), 2.63 (d, 3H, J = 5.0 Hz), 1.88 (s, 3H), 1.65 (s, 3H) ppm; ^{13}C NMR ($CDCl_3$, 125 MHz) δ 137.4, 129.6, 128.6, 128.2, 72.8, 66.9, 57.6, 35.3, 32.6, 29.8 ppm; IR ν 2750, 1345, 1102 cm^{-1}; HRMS calcd for $C_{12}H_{17}BrNO_3S^-$ 334.0118 found 334.0130 (M^-).

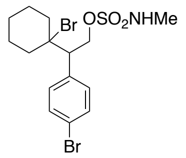

Table 2, Entry 9: Synthesized according to general procedure C; purified by reversed-phase HPLC (Alltima C18, 10 μm, 22 x 250 mm, eluting with gradient flow over 65 min of 0:100→100:0

MeCN/H_2O, retention time of 53-54 min at a flow rate of 12 ml/min); white foam (34 mg, 60%); TLC R_f = 0.31 (5:1 hexanes/EtOAc); ^{1}H NMR ($CDCl_3$, 600 MHz) δ 7.47 (d, 2H, J = 6.0 Hz), 7.30 (d, 2H, J = 6.0 Hz), 4.81 (dd, 1H, J = 12.0, 6.0 Hz), 4.61-4.59 (m, 1H), 4.24-4.19 (m, 1H), 3.22 (dd, 1H, J = 12.0, 6.0 Hz), 2.52 (d, 3H, J = 6.0 Hz), 2.35-2.29 (m, 1H), 1.87-1.78 (m, 1H), 1.70-1.48 (m, 6H), 1.41-1.34 (m, 1H), 1.20-1.08 (m, 1H); ^{13}C NMR ($CDCl_3$, 125 MHz) δ 136.4, 131.7, 131.6, 122.2, 75.5, 72.3, 57.0, 41.2, 39.5, 29.8, 25.1, 22.9, 22.7 ppm; IR ν 2933, 196, 1489 cm^{-1}; HRMS calcd for $C_{15}H_{20}Br_2NO_3S^-$ 453.9516 found 453.9536 (M^-).

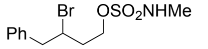

Table 2, Entry 10: Substrate synthesized according to general procedure C; purified by reversed-phase HPLC (Alltima C18, 10 μm, 22 x 250 mm, eluting with gradient flow over 65 min of 0:100 →100:0 MeCN/H_2O, retention time of 45 min at a flow rate of 12 ml/min); clear oil (11 mg, 27%); TLC R_f = 0.39 (10:3 hexanes/EtOAc); ^{1}H NMR ($CDCl_3$, 500 MHz) δ 7.42-7.24 (m, 5H), 4.40-4.34 (m, 4H), 3.28 (dd, 1H, J = 15.0, 5.0 Hz), 3.21 (dd, 1H, J = 15.0, 5.0 Hz), 2.81 (d, 3H, J = 5.0 Hz), 2.40-2.33 (m, 1H), 2.12-2.05 (m, 1H) ppm; ^{13}C NMR ($CDCl_3$, 125 MHz) δ 137.8, 129.5, 128.8, 127.4, 68.7, 52.3, 45.9, 37.2, 30.2 ppm; IR ν 3319, 1413, 148, 1175, 1081 cm^{-1}; HRMS calcd for $C_{11}H_{16}BrNO_3SNa^+$ 343.9926 found 343.9934 (MNa^+).

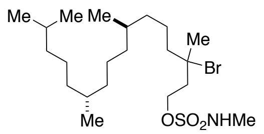

Table 2, Entry 11: Synthesized according to general procedure C; purified by chromatography on silica gel (10:1 pentane/EtOAc); clear oil (47 mg, 80%); TLC R_f = 0.2 (20:3 hexanes/EtOAc); ^{1}H NMR ($CDCl_3$, 300 MHz) δ 4.45-4.34 (m, 3H), 2.87 (d, 3H, J = 3.0 Hz), 2.41-2.21 (m, 2H), 2.00-0.78 (m, 36H) ppm; ^{13}C NMR ($CDCl_3$, 125 MHz) δ 69.5, 68.9, 46.7, 43.7, 39.6, 37.7, 37.6, 37.5, 37.1, 33.1, 32.9, 31.9, 30.1, 28.2, 25.1, 24.7, 23.4, 23.0, 22.9, 20.0, 19.9 ppm; IR ν 2926, 1462, 1350, 1178, 978 cm^{-1}; HRMS (ES^-) calcd for $C_{21}H_{43}BrNO_3S^-$ 468.2147 found 468.2145 (M^-).

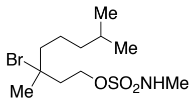

Table 2, Entry 12: Substrate synthesized according to general procedure C; purified by chromatography on silica gel (gradient elutionL 20:1→10:1 hexanes/EtOAc); light yellow oil (30 mg, 72%); TLC R_f = 0.35 (10:3 hexanes/EtOAc); ^{1}H NMR ($CDCl_3$, 400 MHz) δ 4.41-4.34 (m, 3H), 2.84 (d, 3H, J = 4.0 Hz), 2.37-2.29 (m, 1H), 2.24-2.17 (m, 1H), 1.90-1.71 (m, 5H), 1.61-1.43 (m, 3H), 1.25-1.16 (m, 2H), 0.89 (d, 6H, J = 8.0 Hz) ppm; ^{13}C NMR ($CDCl_3$, 125 MHz) δ 69.5, 68.9, 46.6, 43.7, 38.9, 31.9, 30.1, 28.1, 23.7, 22.8 ppm; IR ν 3319, 2955, 1350, 1177, 980 cm^{-1}; HRMS calcd for $C_{11}H_{23}BrNO_3S^-$ 328.0588 found 328.0589 (M^-).

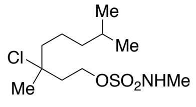

Table 2, Entry 12: Substrate synthesized according to general procedure D; purified by reversed-phase HPLC (Alltima C18, 10 μm, 22 x 250 mm, eluting with gradient flow over 65 min of 0:100 →100:0 MeCN/H_2O, retention time of 52 min at a flow rate of 12 ml/min); clear oil (14 mg,

40%); TLC R_f = 0.32 (5:1 hexanes/EtOAc); ^{1}H NMR ($CDCl_3$, 600 MHz) δ 4.38-4.36 (m, 2H), 4.33-4.30 (m, 1H), 2.83 (d, 3H, J = 6.0 Hz), 2.26-2.25 (m, 1H), 2.22-2.19 (m, 1H), 1.79-1.71 (m, 2H), 1.57-1.56 (m, 4H), 1.48-1.42 (m, 2H), 1.20-1.16 (m, 2H), 0.89 (d, 6H, J = 6.0 Hz) ppm; ^{13}C NMR ($CDCl_3$, 125 MHz) δ 72.1, 67.9, 45.2, 42.5, 39.1, 30.2, 30.1, 28.1, 22.8, 22.6 ppm; IR ν 2954, 1348, 1177, 979 cm^{-1}; HRMS calcd for $C_{11}H_{23}ClNO_3S^-$ 284.1093 found 284.1107 (M^-).

Figure S1. Conversion of (1*R*,2*R*,5*R*)-2-(2-bromopropan-2-yl)-5-methylcyclohexyl methylsulfamate to the *N*-methyl sulfamate of isopulegol induced by silica gel chromatography.

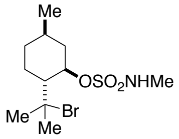

(1*R*,2*R*,5*R*)-2-(2-bromopropan-2-yl)-5-methylcyclohexyl methylsulfamate

(a) ^{1}H NMR of unpurified reaction mixture ($CDCl_3$, 600 MHz)

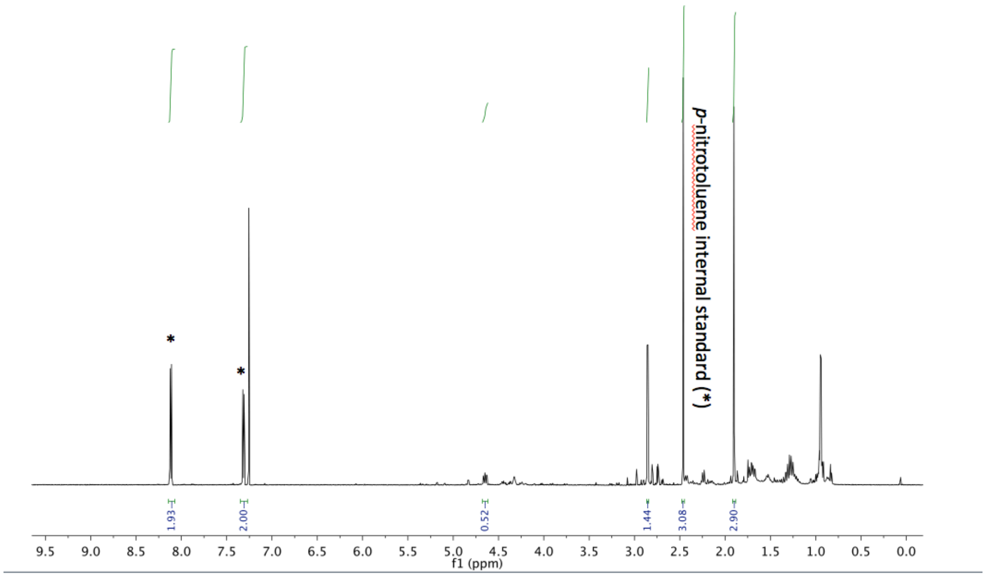

(b) ^{1}H NMR post silica gel chromatography ($CDCl_3$, 500 MHz)

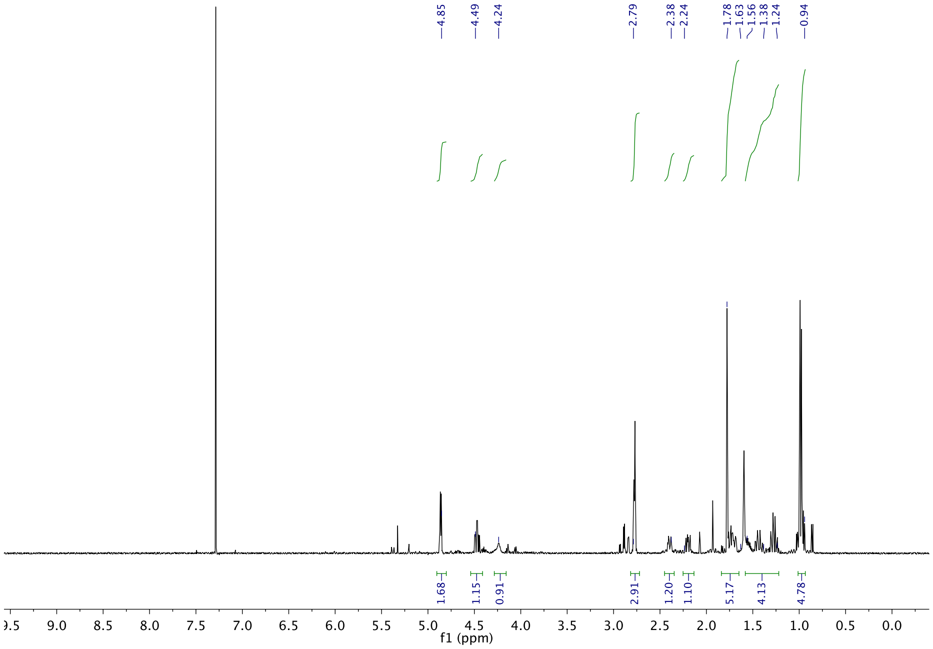

(c) ^{1}H NMR ($CDCl_3$, 500 MHz) (authentic sample)

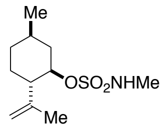

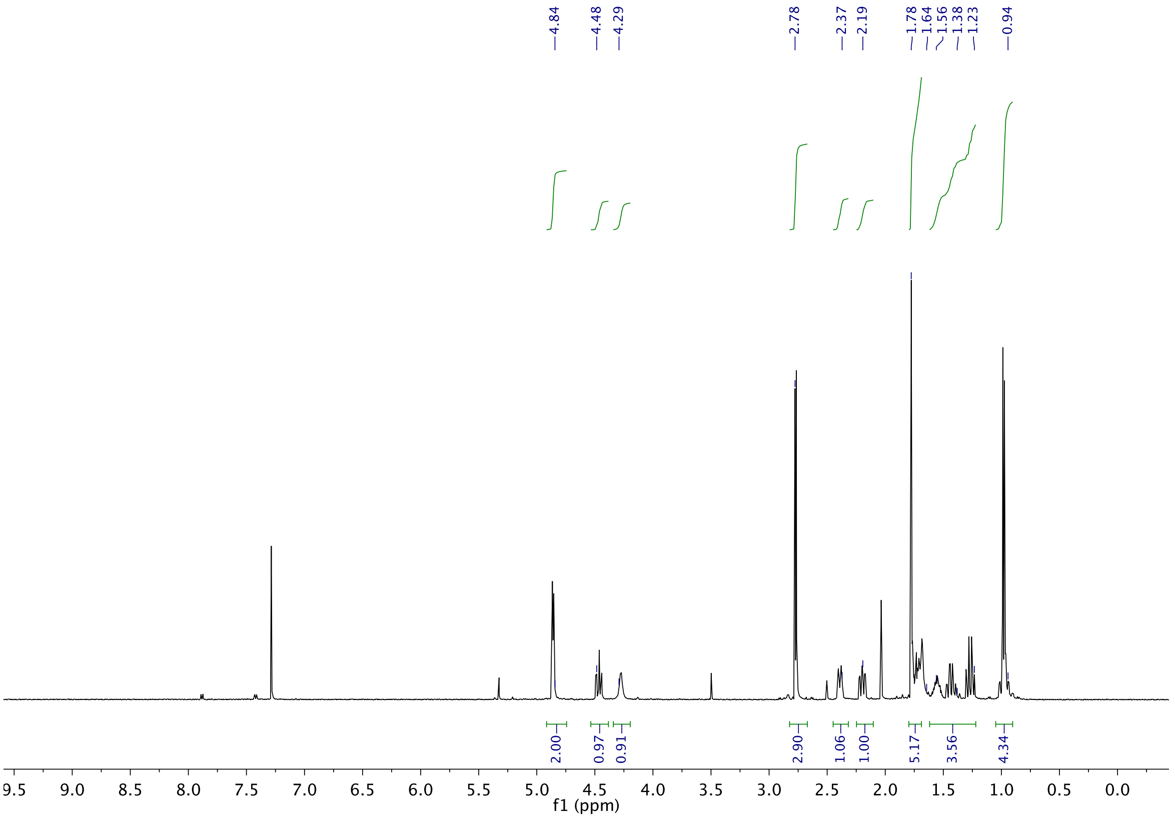

Figure S2. Evaluation of reaction stereospecificity (Table 2, entry 12)

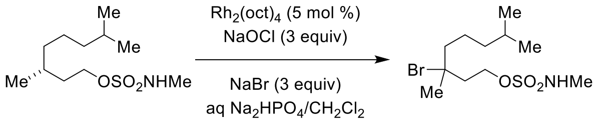

(a) HPLC trace of racemic product

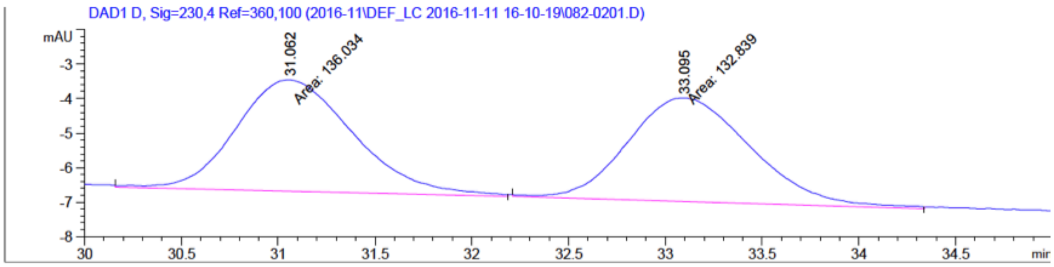

Peak #	RetTime [min]	Type	Width [min]	Area [mAU*s]	Height [mAU]	Area %
1	31.062	MM	0.7035	136.03418	3.22276	50.5942
2	33.095	MM	0.7372	132.83896	3.00341	49.4058
Totals :				268.87314	6.22617	

(b) HPLC trace of reaction product

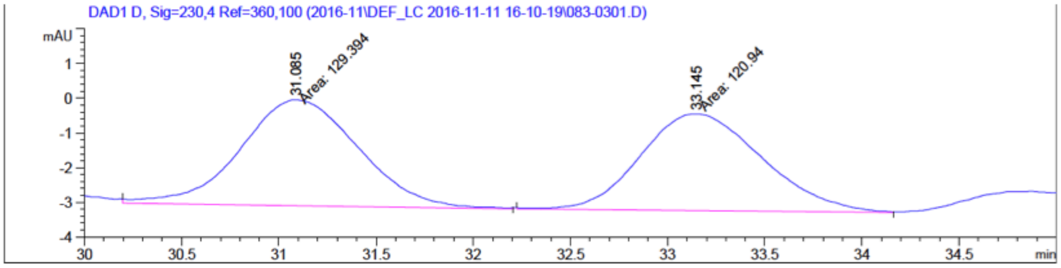

Peak #	RetTime [min]	Type	Width [min]	Area [mAU*s]	Height [mAU]	Area %
1	31.085	FM	0.7055	129.39417	3.05690	51.6886
2	33.145	MM	0.7253	120.94012	2.77924	48.3114
Totals :				250.33429	5.83614	

Scheme 1. Nucleophilic displacement of *N*-methyl sulfamate auxiliary.

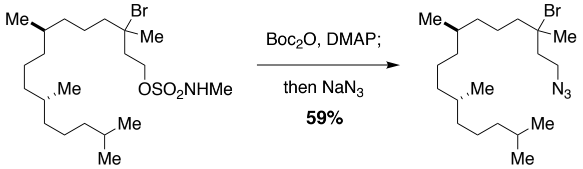

To a solution of (7*R*,11*R*)-3-bromo-3,7,11,15-tetramethylhexadecyl methylsulfamate (47 mg, 0.100 mmol, 1.0 equiv) in 1.0 mL of CH_2Cl_2 was added Boc_2O (30 mg, 0.14 mmol, 1.4 equiv) and DMAP (13 mg, 0.110 mmol, 1.10 equiv). After stirring for 1 h, the reaction mixture was concentrated under reduced pressure to a light yellow oil. The product was redissolved 1.0 mL of DMSO and to this solution was added NaN_3 (20 mg, 0.31 mmol, 3.1 equiv). After stirring this mixture for 2 h, the reaction contents were transferred to a 150 mL separatory funnel with 30 mL of Et_2O. The organic layer was washed with 100 mL of saturated aqueous NaCl, dried over Na_2SO_4, and concentrated under reduced pressure to a yellow oil. Purification of this material by chromatography on silica gel afforded the desired product as a clear oil (23 mg, 59%); TLC R_f = 0.5 (20:1 hexanes/EtOAc); ^{1}H NMR ($CDCl_3$, 600 MHz) δ 3.56-3.50 (m, 2H), 2.19-2.12 (m, 1H), 2.07-1.99 (m, 1H), 1.89-1.04 (m, 24H), 0.93-0.82 (m, 12H) ppm; ^{13}C NMR ($CDCl_3$, 500 MHz) δ 70.0, 49.2, 46.5, 43.8, 39.6, 37.7, 37.6, 37.5, 37.1, 33.1, 32.9, 31.8, 28.2, 25.1, 24.7, 23.4, 23.0, 22.9, 20.0, 19.9 ppm; IR ν 2926, 2096, 1379, 1261 cm^{-1}; HRMS calcd for $C_{20}H_{40}BrN_3K^+$ 440.2037 found 440.2050 (MK^+).

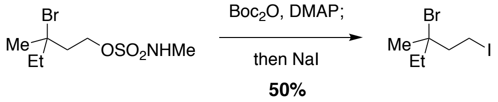

To a solution of 3-bromo-3-methylpentyl methylsulfamate (17 mg, 61 μmol) in 1.0 mL of CH_2Cl_2 was added Boc_2O (20 mg, 92 μmol, 1.50 equiv) and DMAP (9 mg, 74 μmol, 1.2 equiv). After stirring for 1 h, the reaction mixture was concentrated under reduced pressure to a light yellow residue. The product was redissolved in 1.0 mL of acetone and to this solution was added NaI (30 mg, 0.20 mmol, 3.3 equiv). After stirring this mixture for 5 h at 35 °C, the reaction was cooled and the contents were transferred to a 60 mL separatory funnel with 10 mL of CH_2Cl_2. The organic layer was washed with 15 mL of H_2O, dried over Na_2SO_4, and concentrated under reduced pressure to a yellow oil. Purification of this material by chromatography on silica gel (hexanes) afforded the desired product as a clear oil (9 mg, 52%); TLC R_f = 0.5 (hexanes); ^{1}H NMR ($CDCl_3$, 500 MHz) δ 3.40-3.30 (m, 2H), 2.52-2.46 (m, 1H), 2.37-2.32 (m, 1H), 1.95-1.90 (m, 1H), 1.86-1.78 (m, 1H), 1.71 (s, 3H), 1.06 (t, 3H, J = 7.4 Hz) ppm; ^{13}C NMR ($CDCl_3$, 500 MHz) δ 73.4, 49.9, 38.3, 30.6, 10.4, -0.1 ppm; IR ν 2922, 1558, 1380, 1156 cm^{-1}.

Figure S3. Stirring *N*-bromo sulfamate **5** with $Rh_2(oct)_4$ yields 40% of halogenated product.

Synthesis of *N*-bromo sulfamate **5**:

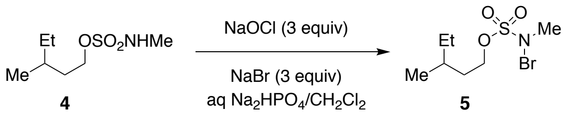

To a solution of 3-methylpentyl methylsulfamate (24 mg, 0.122 mmol) in 1.2 mL of saturated aqueous Na_2HPO_4 and 1.2 mL of CH_2Cl_2 was added NaBr (38 mg, 0.37 mmol, 3.0 equiv). The reaction flask was wrapped in aluminum foil, and aqueous NaOCl (10-15% active chlorine, 230 μL, 0.37 mmol, 3.0 equiv) was added. After stirring for 1 h, the reaction mixture was transferred to a 60 mL separatory funnel with 10 mL of CH_2Cl_2 and 10 mL of H_2O. The organic layer was collected, dried over Na_2SO_4, and concentrated under reduced pressure to a light yellow oil, which was used without further purification in subsequent experiments.

^{1}H NMR (500 MHz, $CDCl_3$) of unpurified **5**

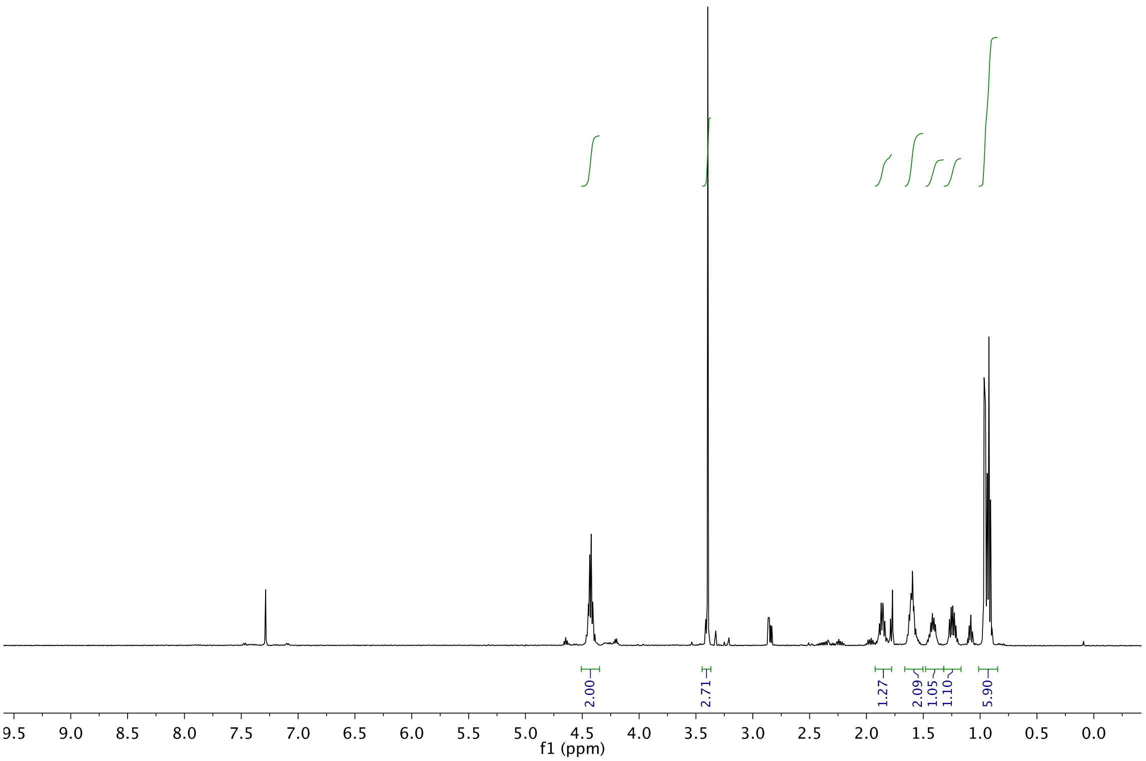

^1H NMR (600 MHz, $CDCl_3$) of the unpurified reaction mixture of **5** and $Rh_2(oct)_2$

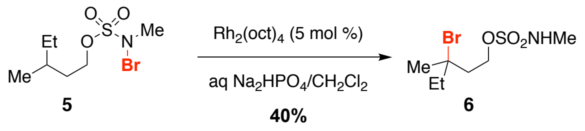

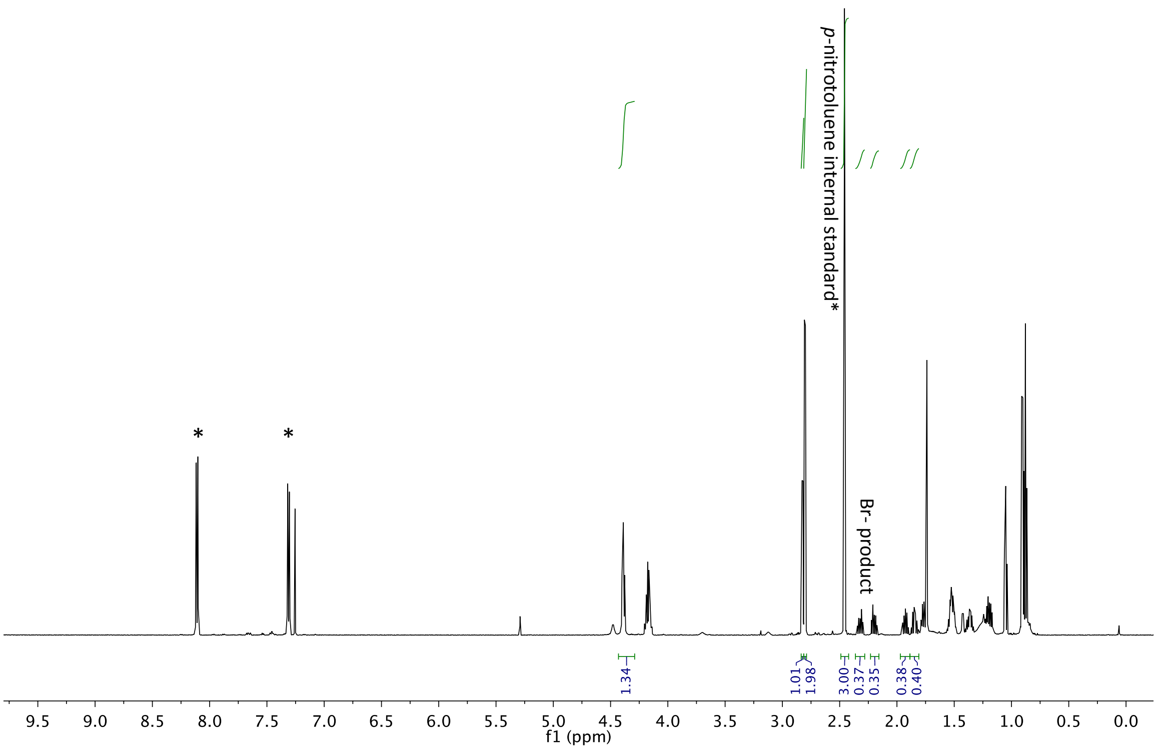

Figure S4. (a) UV/vis shows decomposition of $Rh_2(oct)_4$ within 30 min of NaOCl addition.

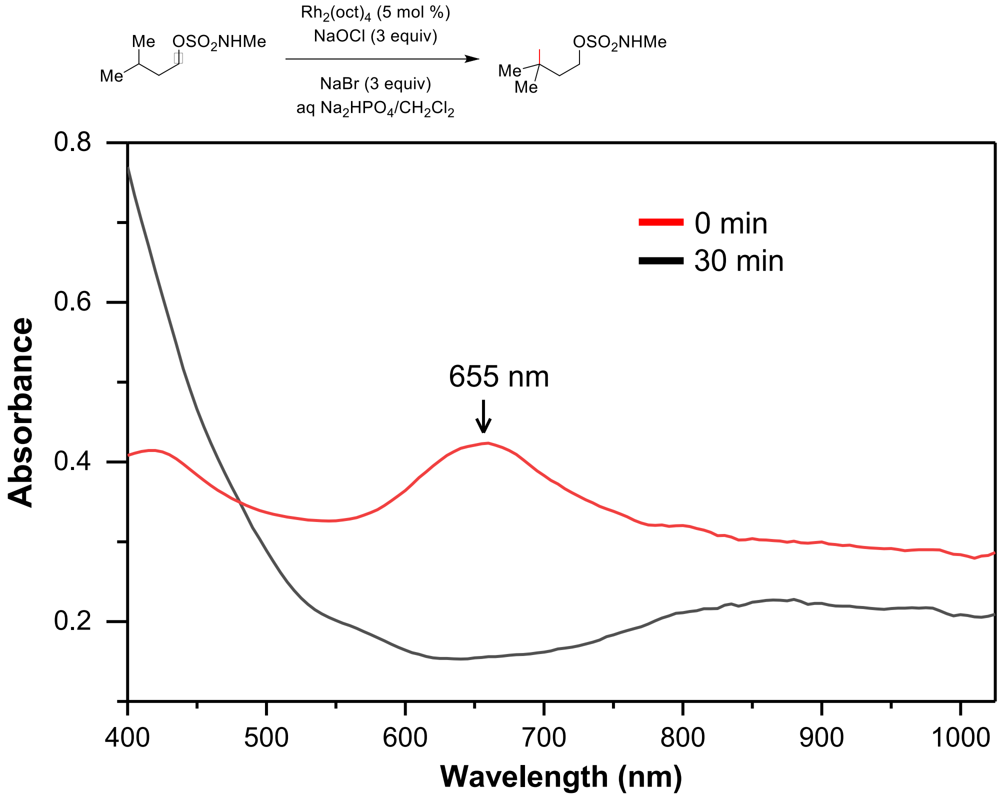

(b) ^{1}H NMR (600 MHz, $CDCl_3$) after 30 min of reaction shows ~30 % yield.

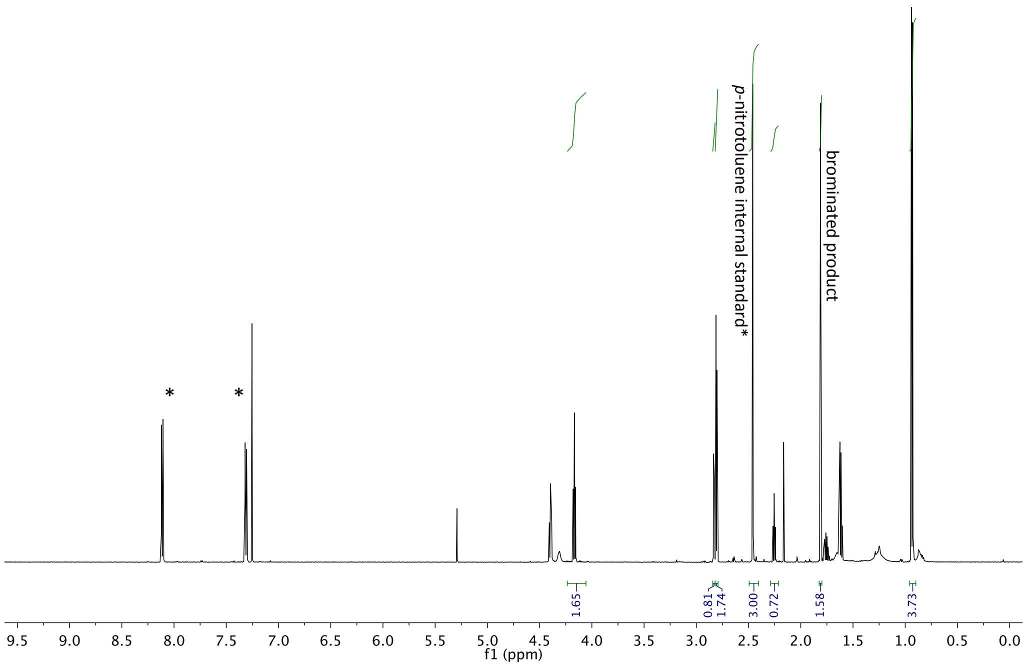

Figure S5. (a) Synthesis of *N*-chlorosulfamate **10**

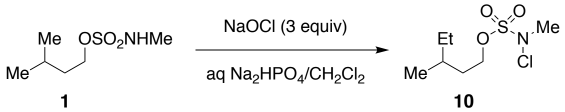

To a solution of **1** (24 mg, 0.122 mmol) in 1.2 mL of saturated aqueous Na_2HPO_4 and 1.2 mL of CH_2Cl_2 was added aqueous NaOCl (10-15% active chlorine, 230 µL, 0.37 mmol, 3.0 equiv). The reaction flask was wrapped in aluminum foil. After stirring for 1 h, the reaction mixture was transferred to a 60 mL separatory funnel with 10 mL of CH_2Cl_2 and 10 mL of H_2O. The organic layer was collected, dried over Na_2SO_4, and concentrated under reduced pressure to a light yellow oil which was used without further purification in subsequent experiments.

^{1}H NMR (500 MHz, $CDCl_3$) of unpurified **10**

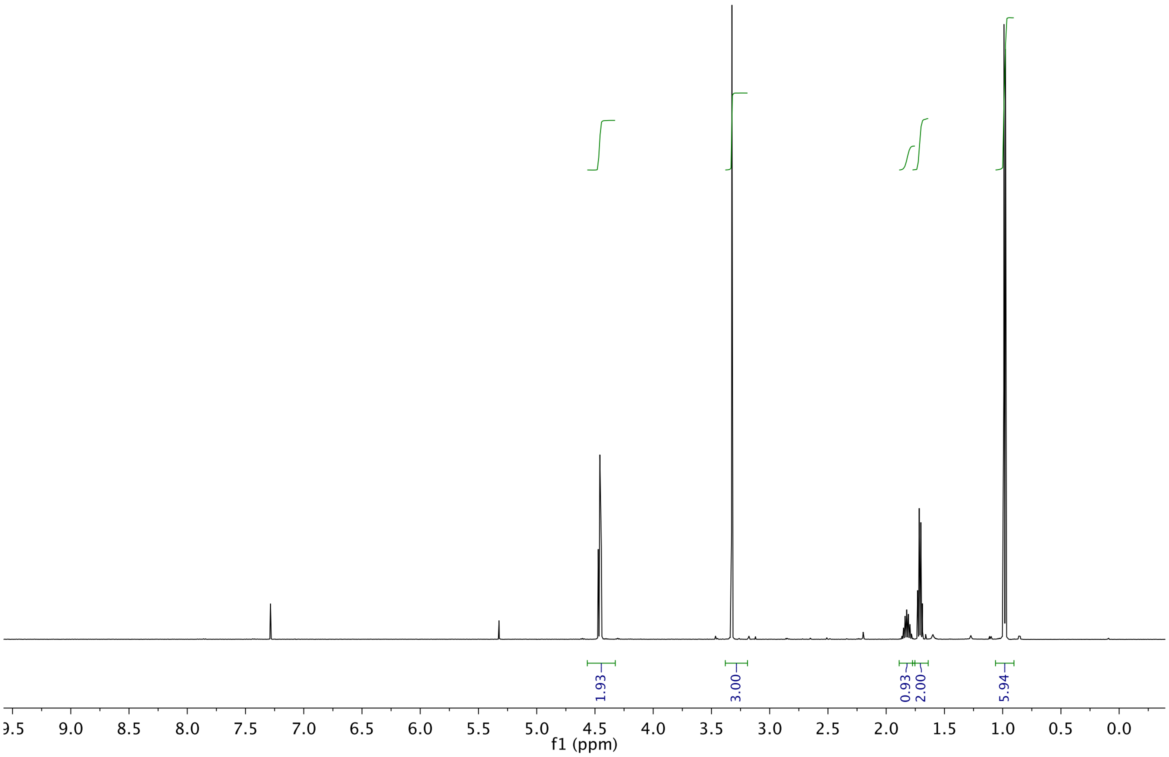

Figure S5. (b) Collision-induced dissociation (MS/MS) data of the halogenated species using ESI-MS in negative ion mode.

**Observed m/z for $[M-H]^-$**	**Chemical formula (M)**	**Theoretical m/z for $[M-H]^-$**	**Deviation (ppm)**	**Attribution[#]**	**Characteristic CID fragments* (m/z)**
257.9808	$C_6H_{14}BrNO_3S$	257.9805	1.16	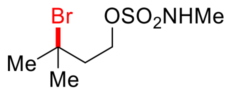	196.7767, 189.9479, 178.0537, 165.0222, 157.9219, 109.9916, 96.9601
228.0471	$C_7H_{17}ClNO_3S$	228.0467	1.75	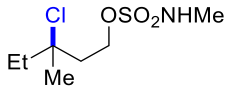	196.7834, 192.0329, 177.8944, 175.8974, 147.9041, 146.9047, 145.9083, 143.9012, 134.8986, 123.9708, 109.9916, 98.9559, 97.9595, 96.9601, 79.9574

[#]Species are attributed based on their isotopic distribution patterns in the MS1 spectra and CID studies.

*CID fragments matched with the fragments of the corresponding standards, which were synthesized separately as described in the experimental section.

Figure S6. KIE measurement.

The reaction was performed according to the general procedure for oxidative bromination (procedure C). The ratio of protiated to deuterated compounds was determined to be 3:1 based on HRMS ion counts.

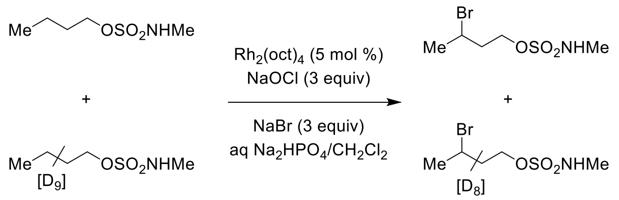

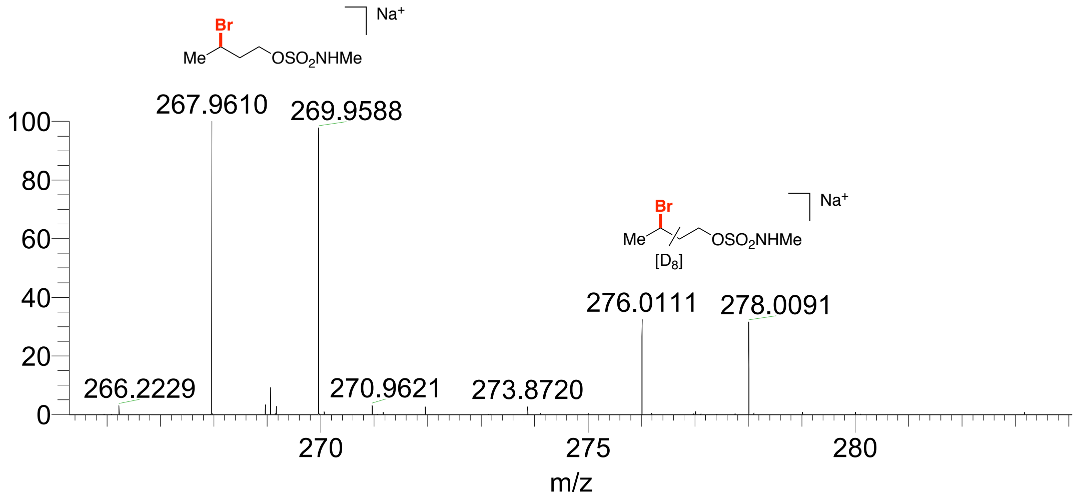

Figure S7. $CuBr_2$/1,10-phenanthroline initiates radical chain C–H bromination.

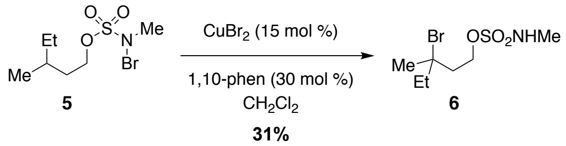

^{1}H NMR (500 MHz, $CDCl_3$) of the unpurified reaction mixture

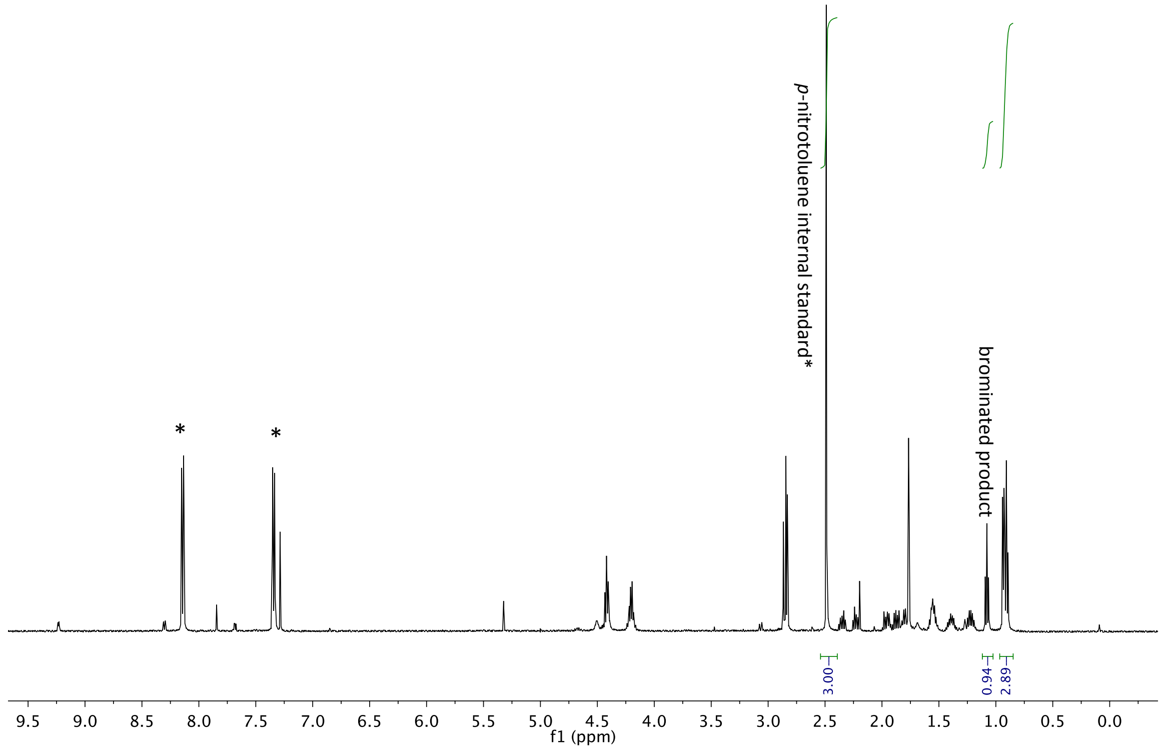

Figure S8. CuBr/1,10-phenanthroline initiates radical chain C–H bromination.

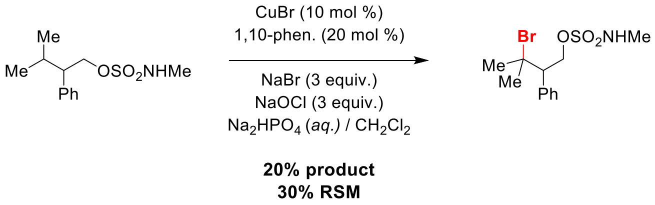

^{1}H NMR (400 MHz, $CDCl_3$) of the unpurified reaction mixture

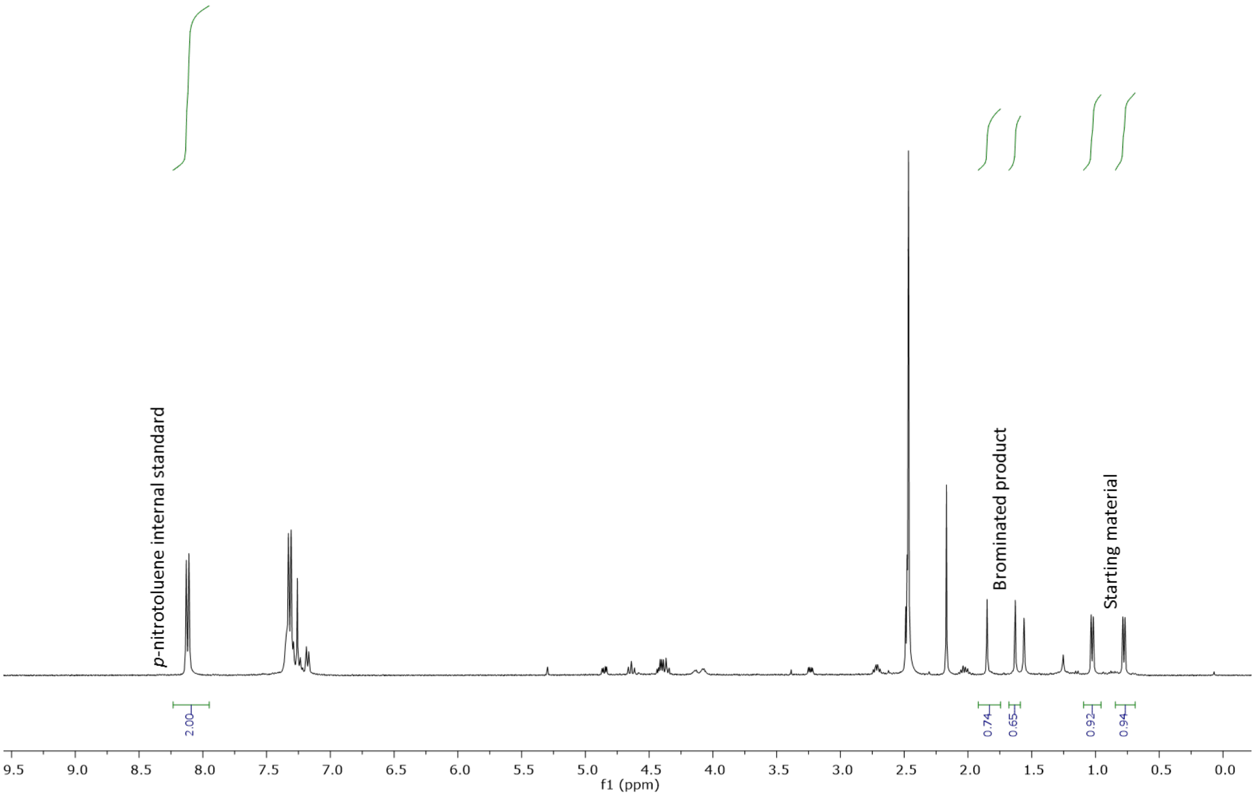

^{1}H and ^{13}C NMR Spectra

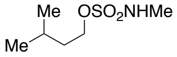

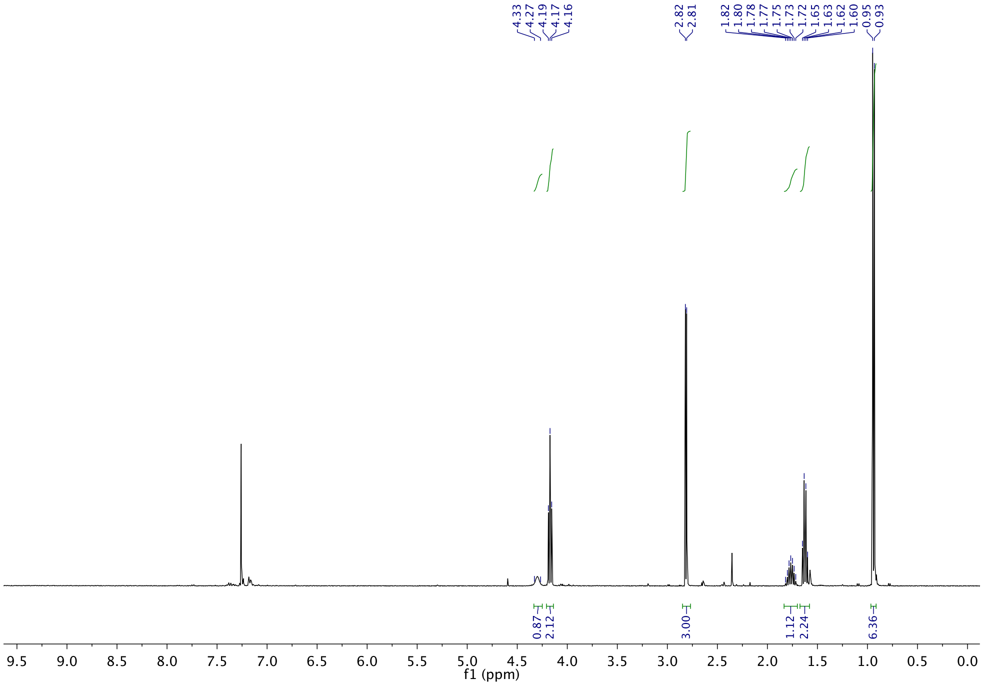

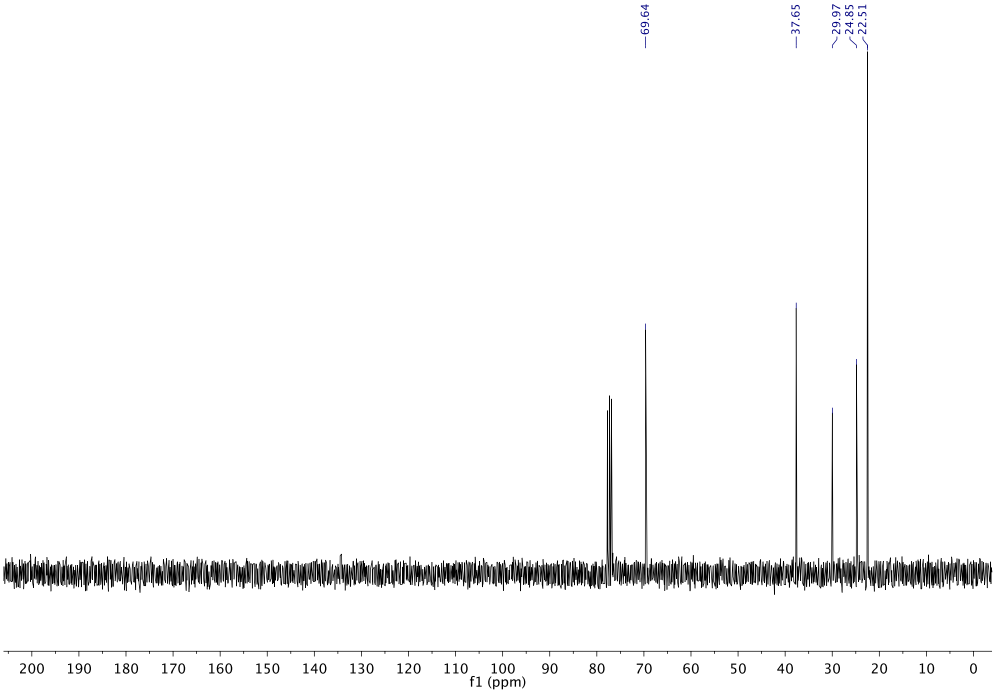

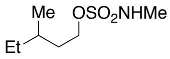
Me
OSO2NHMe
Et

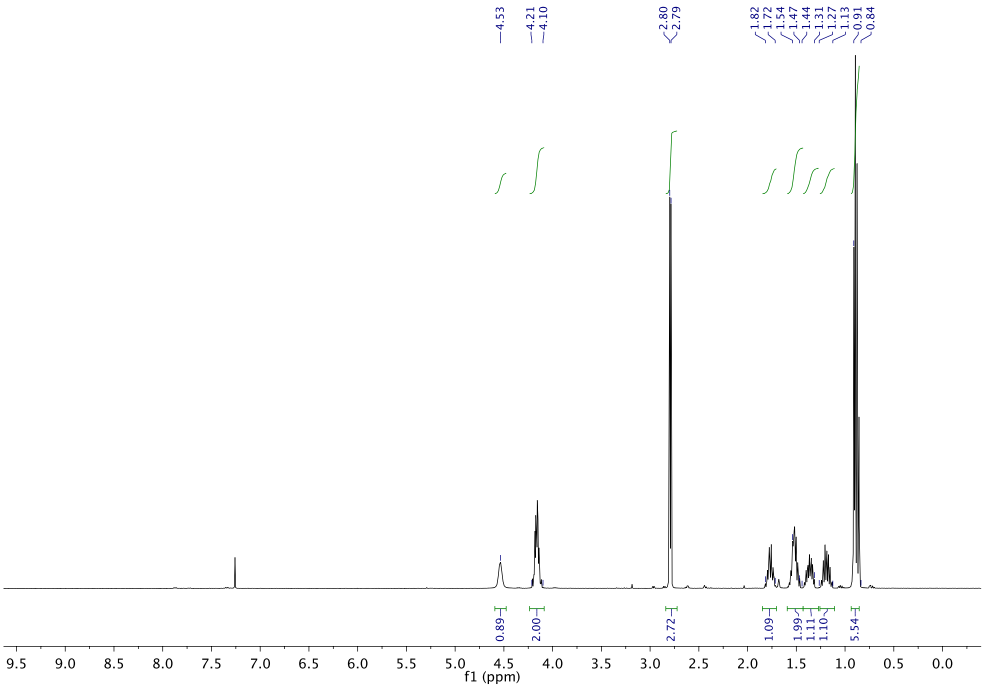
4.53
4.21
4.10
2.80
2.79
1.82
1.72
1.54
1.47
1.44
1.31
1.27
1.13
0.91
0.84
0.89
2.00
2.72
1.09
1.99
1.11
1.10
5.54
9.5 9.0 8.5 8.0 7.5 7.0 6.5 6.0 5.5 5.0 4.5 4.0 3.5 3.0 2.5 2.0 1.5 1.0 0.5 0.0
f1 (ppm)

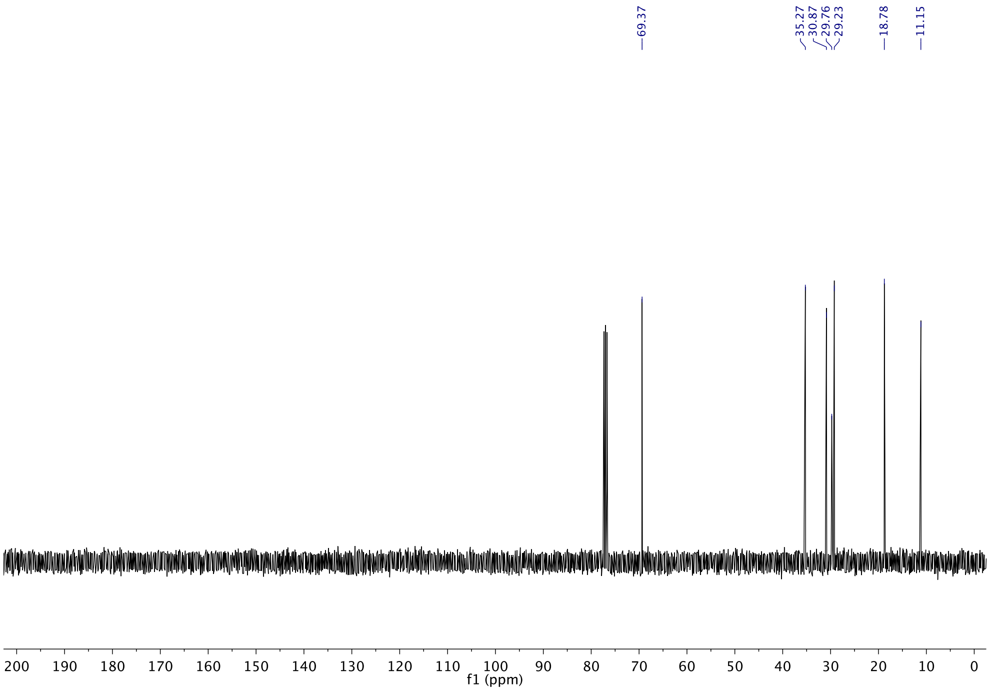
69.37
35.27
30.87
29.76
29.23
18.78
11.15
200 190 180 170 160 150 140 130 120 110 100 90 80 70 60 50 40 30 20 10 0
f1 (ppm)

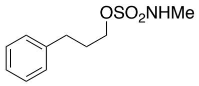
OSO2NHMe

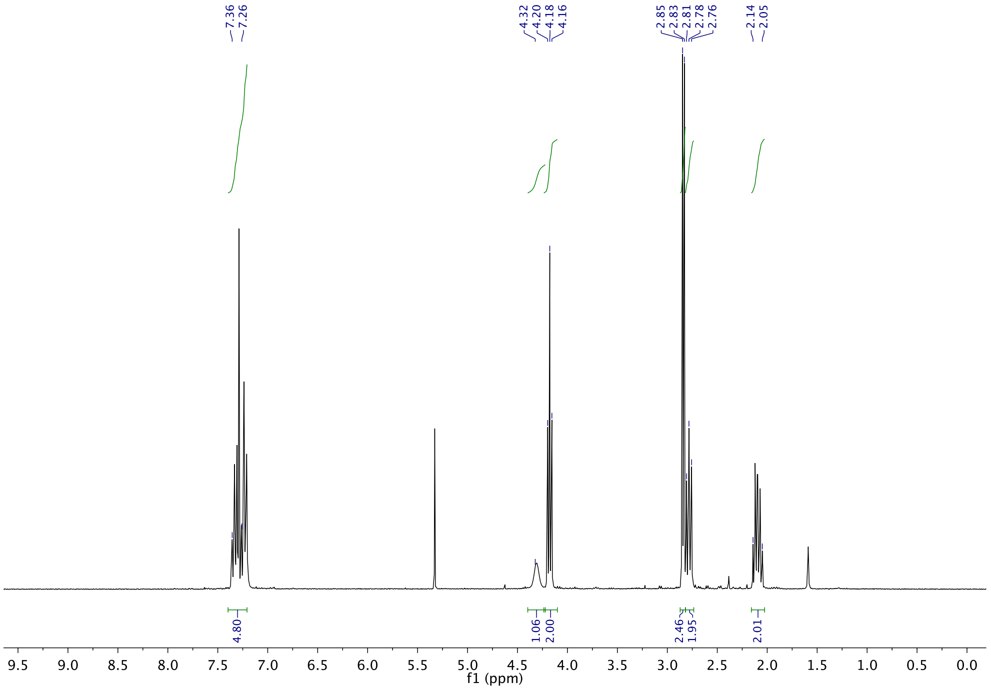
7.36
7.26
4.32
4.20
4.18
4.16
2.85
2.83
2.81
2.78
2.76
2.14
2.05
4.80
1.06
2.00
2.46
1.95
2.01
9.5 9.0 8.5 8.0 7.5 7.0 6.5 6.0 5.5 5.0 4.5 4.0 3.5 3.0 2.5 2.0 1.5 1.0 0.5 0.0
f1 (ppm)

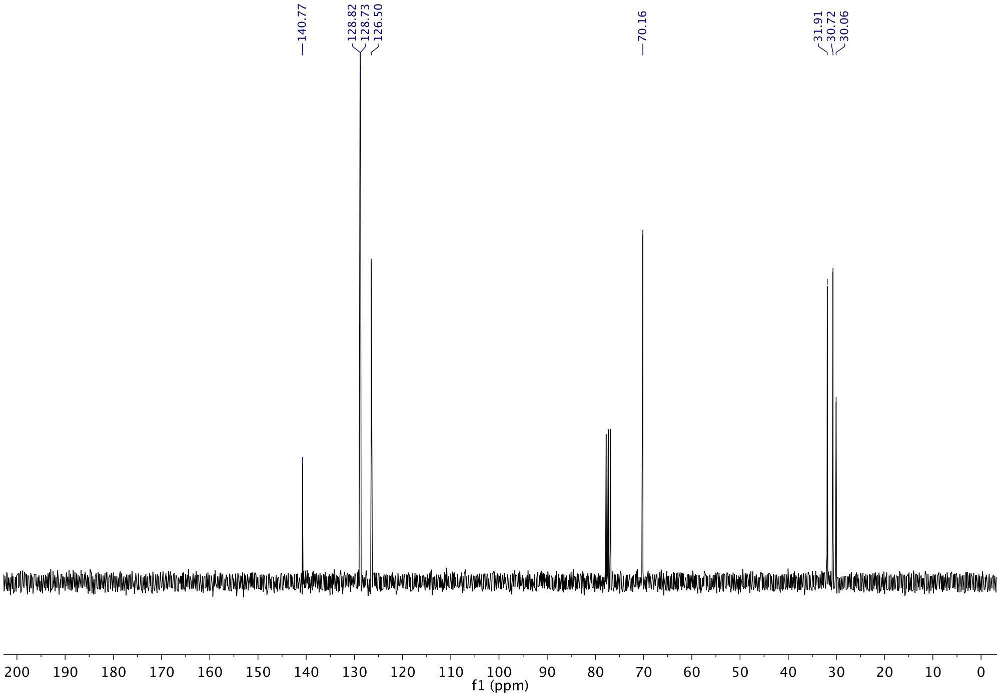
140.77
128.82
128.73
126.50
70.16
31.91
30.72
30.06
200 190 180 170 160 150 140 130 120 110 100 90 80 70 60 50 40 30 20 10 0
f1 (ppm)

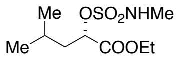
Me OSO2NHMe
Me COOEt

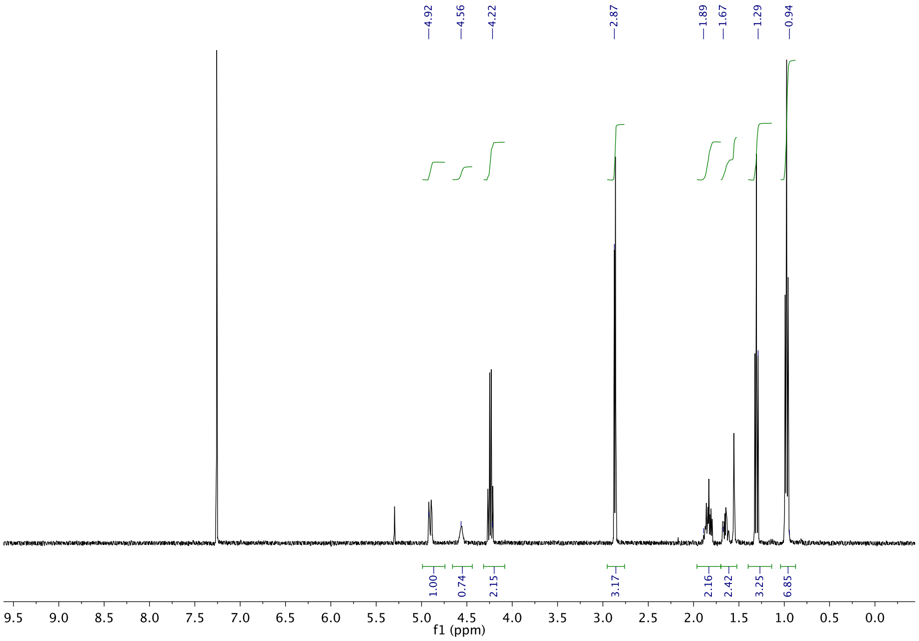
4.92 4.56 4.22 2.87 1.89 1.67 1.29 0.94
1.00 0.74 2.15 3.17 2.16 2.42 3.25 6.85
9.5 9.0 8.5 8.0 7.5 7.0 6.5 6.0 5.5 5.0 4.5 4.0 3.5 3.0 2.5 2.0 1.5 1.0 0.5 0.0
f1 (ppm)

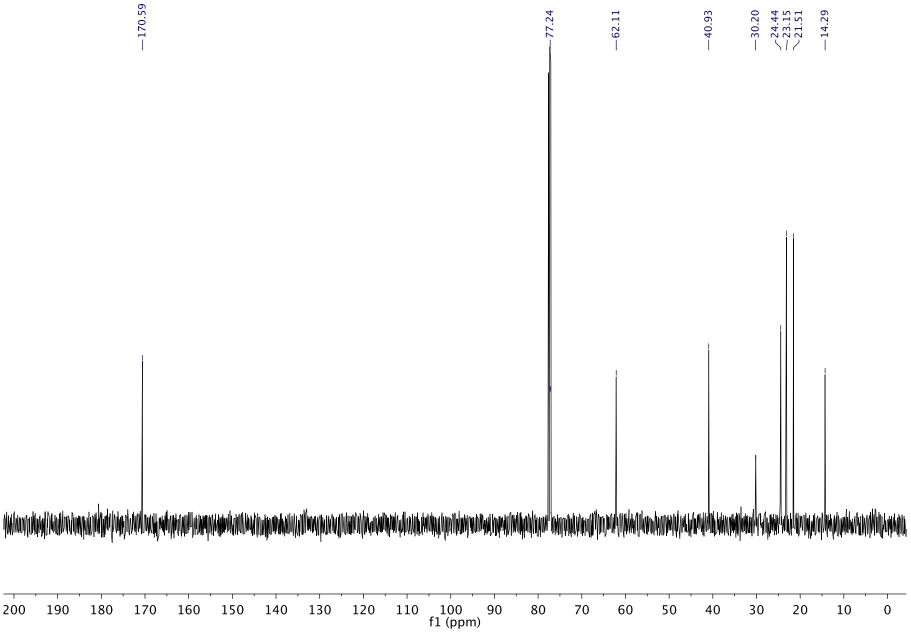
170.59 77.24 62.11 40.93 30.20 24.44 23.15 21.51 14.29
200 190 180 170 160 150 140 130 120 110 100 90 80 70 60 50 40 30 20 10 0
f1 (ppm)

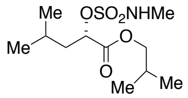
Me
OSO2NHMe
Me
O
O
Me
Me

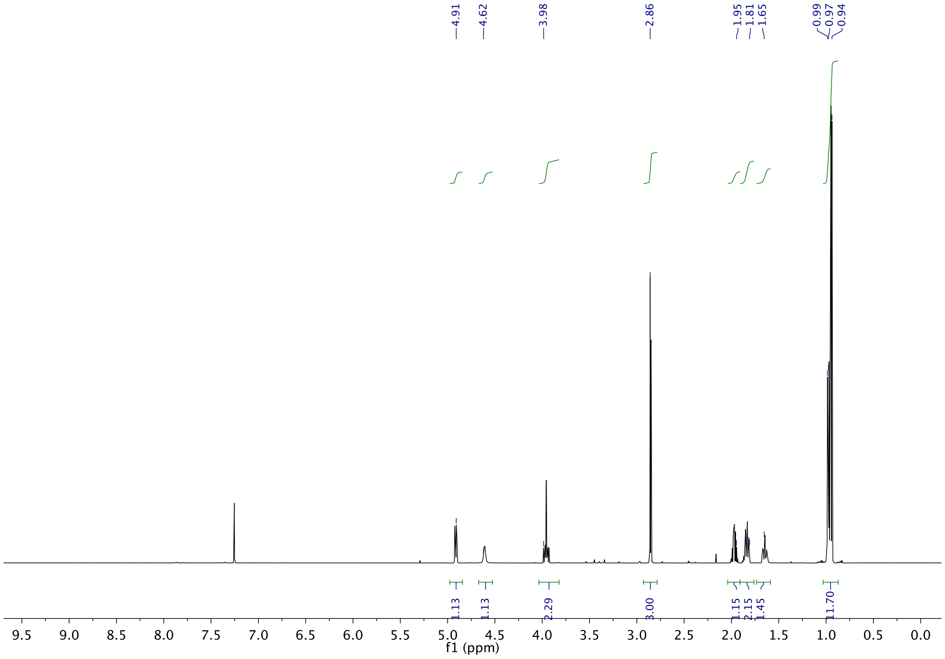
4.91
4.62
3.98
2.86
1.95
1.81
1.65
0.99
0.97
0.94
1.13
1.13
2.29
3.00
1.15
2.15
1.45
11.70
9.5 9.0 8.5 8.0 7.5 7.0 6.5 6.0 5.5 5.0 4.5 4.0 3.5 3.0 2.5 2.0 1.5 1.0 0.5 0.0
f1 (ppm)

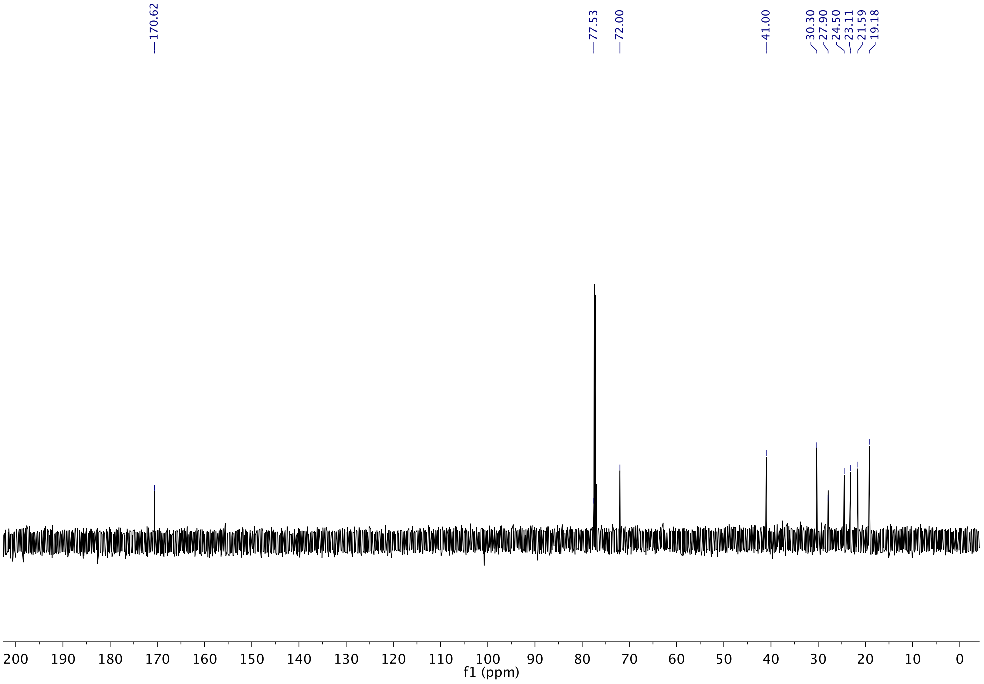
170.62
77.53
72.00
41.00
30.30
27.90
24.50
23.11
21.59
19.18
200 190 180 170 160 150 140 130 120 110 100 90 80 70 60 50 40 30 20 10 0
f1 (ppm)

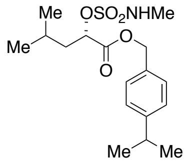
Me
OSO2NHMe
Me
O
O
Me
Me

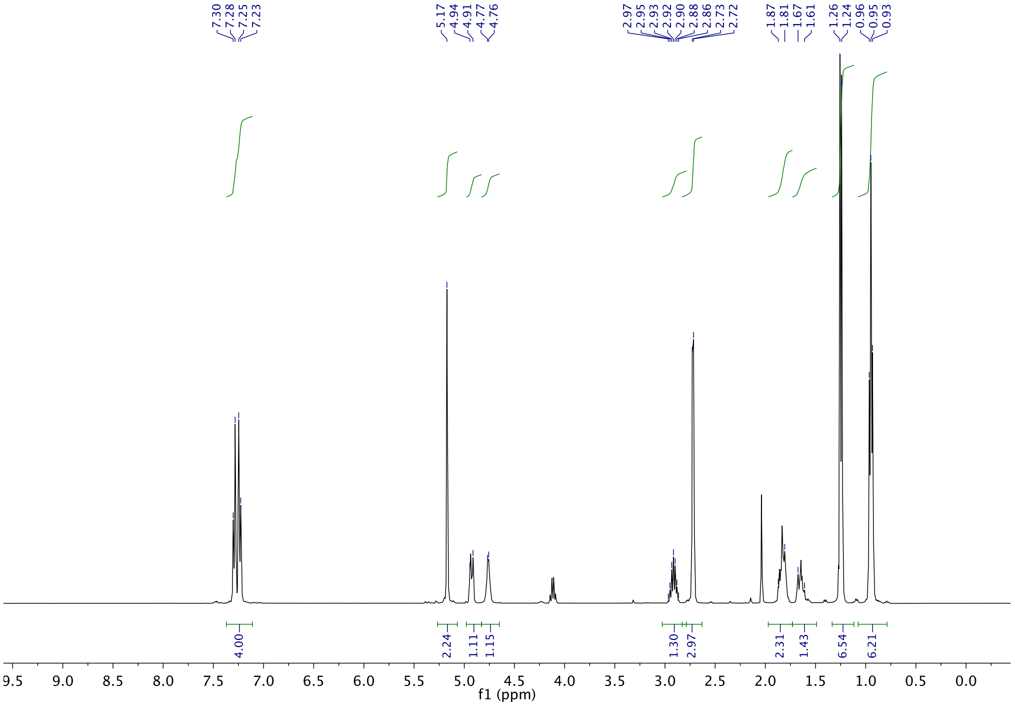
7.30
7.28
7.25
7.23
5.17
4.94
4.91
4.77
4.76
2.97
2.95
2.93
2.92
2.90
2.88
2.86
2.73
2.72
1.87
1.81
1.67
1.61
1.26
1.24
0.96
0.95
0.93
4.00
2.24
1.11
1.15
1.30
2.97
2.31
1.43
6.54
6.21
9.5 9.0 8.5 8.0 7.5 7.0 6.5 6.0 5.5 5.0 4.5 4.0 3.5 3.0 2.5 2.0 1.5 1.0 0.5 0.0
f1 (ppm)

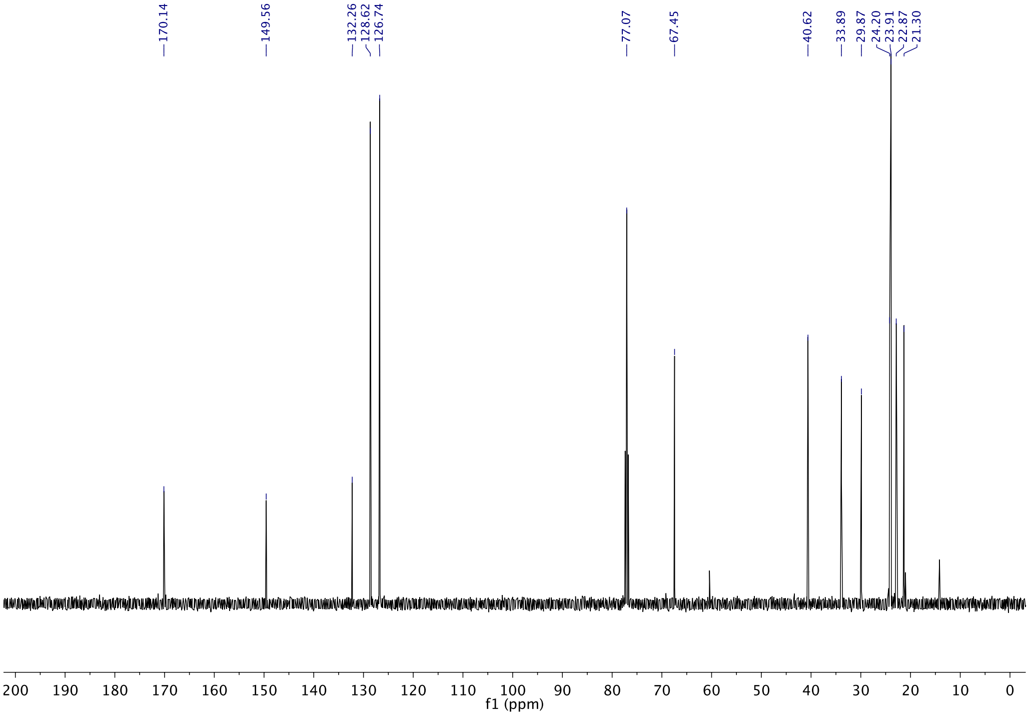
170.14
149.56
132.26
128.62
126.74
77.07
67.45
40.62
33.89
29.87
24.20
23.91
22.87
21.30
200 190 180 170 160 150 140 130 120 110 100 90 80 70 60 50 40 30 20 10 0
f1 (ppm)

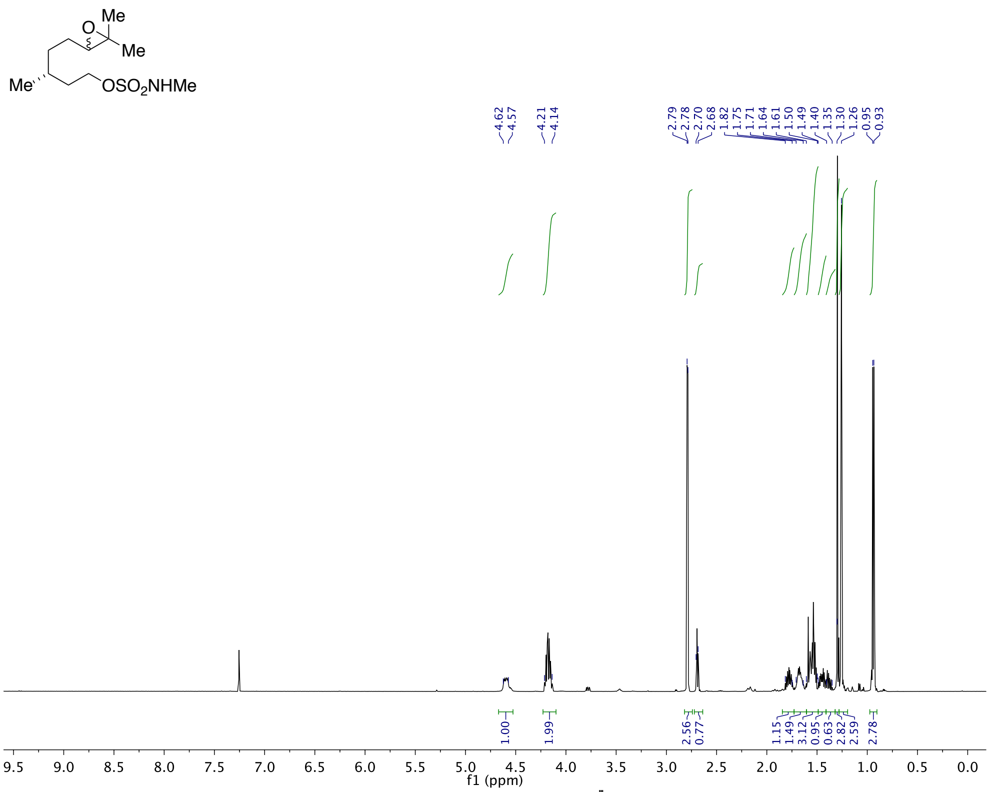
Me
O
Me
Me
OSO2NHMe
4.62
4.57
4.21
4.14
2.79
2.78
2.70
2.68
1.82
1.75
1.71
1.64
1.61
1.50
1.49
1.40
1.35
1.30
1.26
0.95
0.93
1.00
1.99
2.56
0.77
1.15
1.49
3.12
0.95
0.63
2.82
2.59
2.78
f1 (ppm)

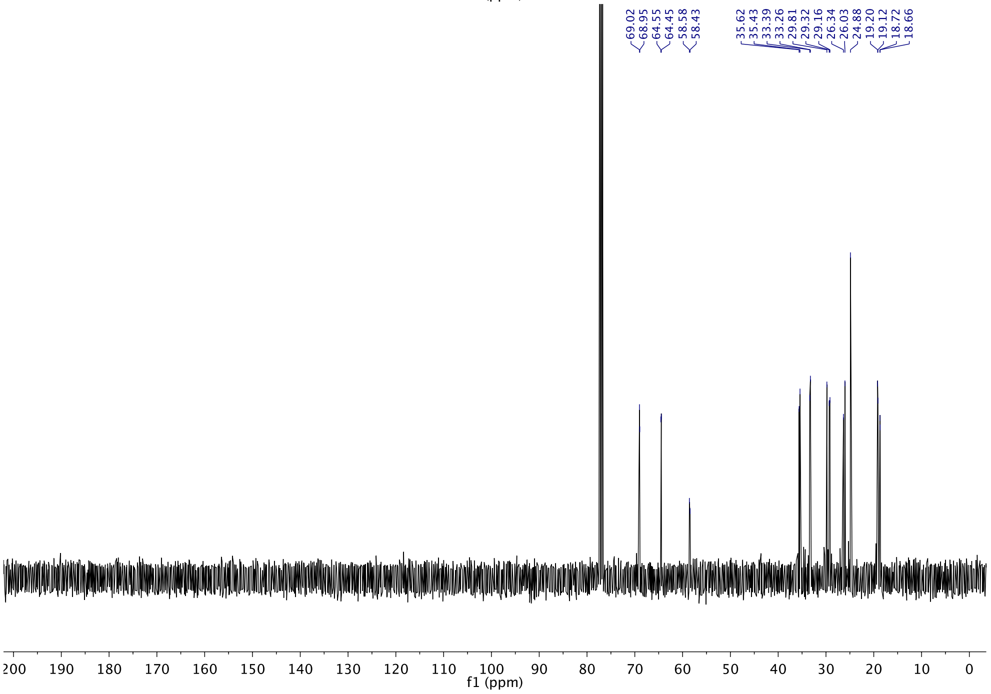
69.02
68.95
64.55
64.45
58.58
58.43
35.62
35.43
33.39
33.26
29.81
29.32
29.16
26.34
26.03
24.88
19.20
19.12
18.72
18.66
f1 (ppm)

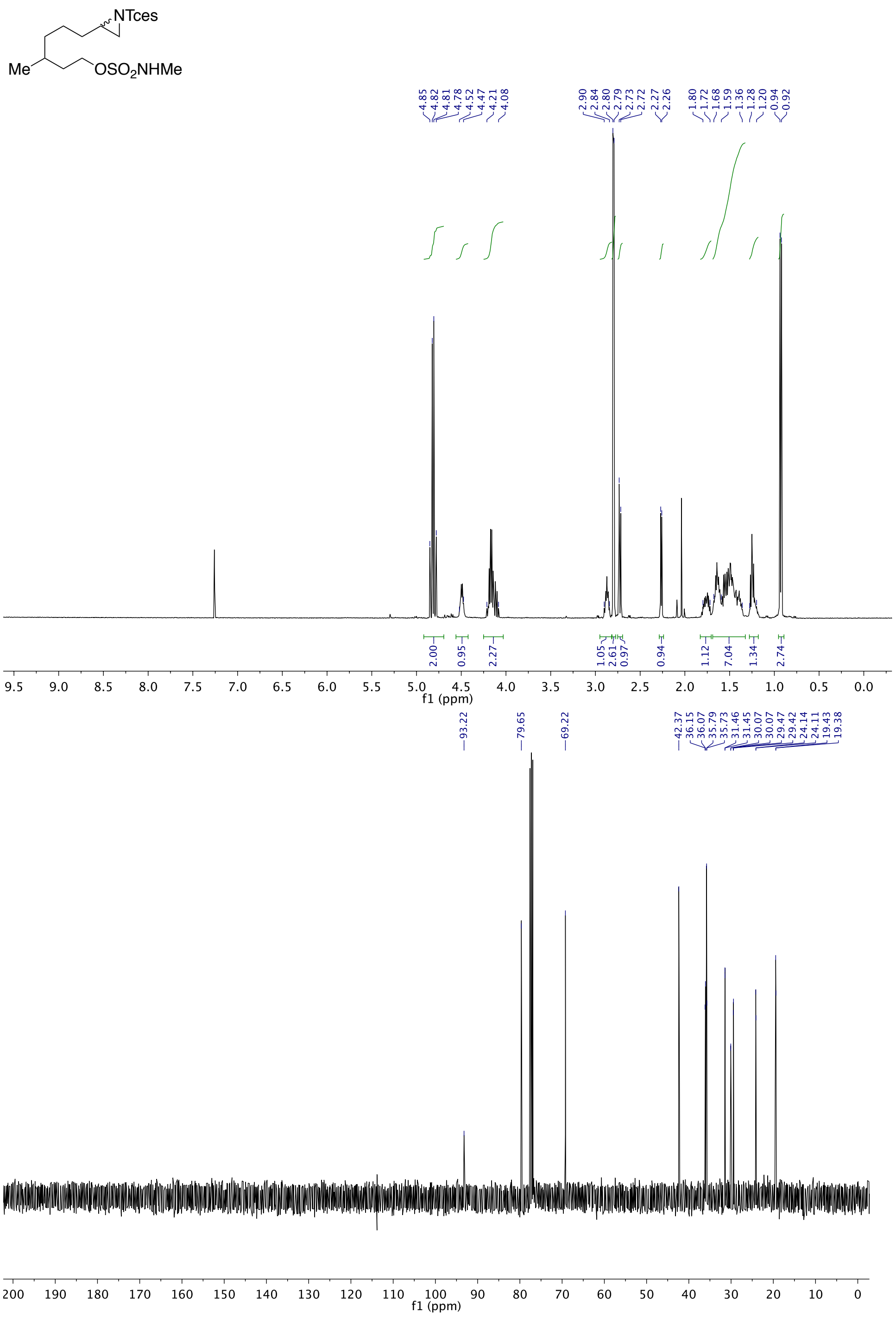
NTces
Me
OSO2NHMe
4.85
4.82
4.81
4.78
4.52
4.47
4.21
4.08
2.90
2.84
2.80
2.79
2.73
2.72
2.27
2.26
1.80
1.72
1.68
1.59
1.36
1.28
1.20
0.94
0.92
2.00
0.95
2.27
1.05
2.61
0.97
0.94
1.12
7.04
1.34
2.74
f1 (ppm)
93.22
79.65
69.22
42.37
36.15
36.07
35.79
35.73
31.46
31.45
30.07
29.47
29.42
24.14
24.11
19.43
19.38
f1 (ppm)

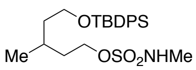
OTBDPS
Me
OSO2NHMe

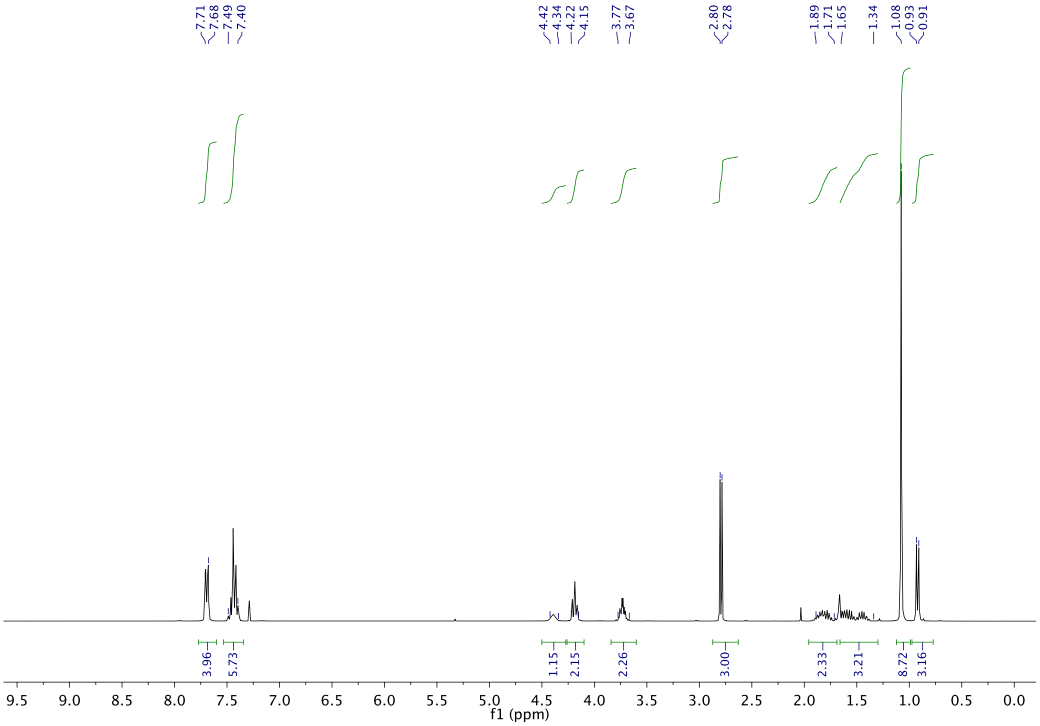
7.71
7.68
7.49
7.40
4.42
4.34
4.22
4.15
3.77
3.67
2.80
2.78
1.89
1.71
1.65
1.34
1.08
0.93
0.91
3.96
5.73
1.15
2.15
2.26
3.00
2.33
3.21
8.72
3.16
9.5 9.0 8.5 8.0 7.5 7.0 6.5 6.0 5.5 5.0 4.5 4.0 3.5 3.0 2.5 2.0 1.5 1.0 0.5 0.0
f1 (ppm)

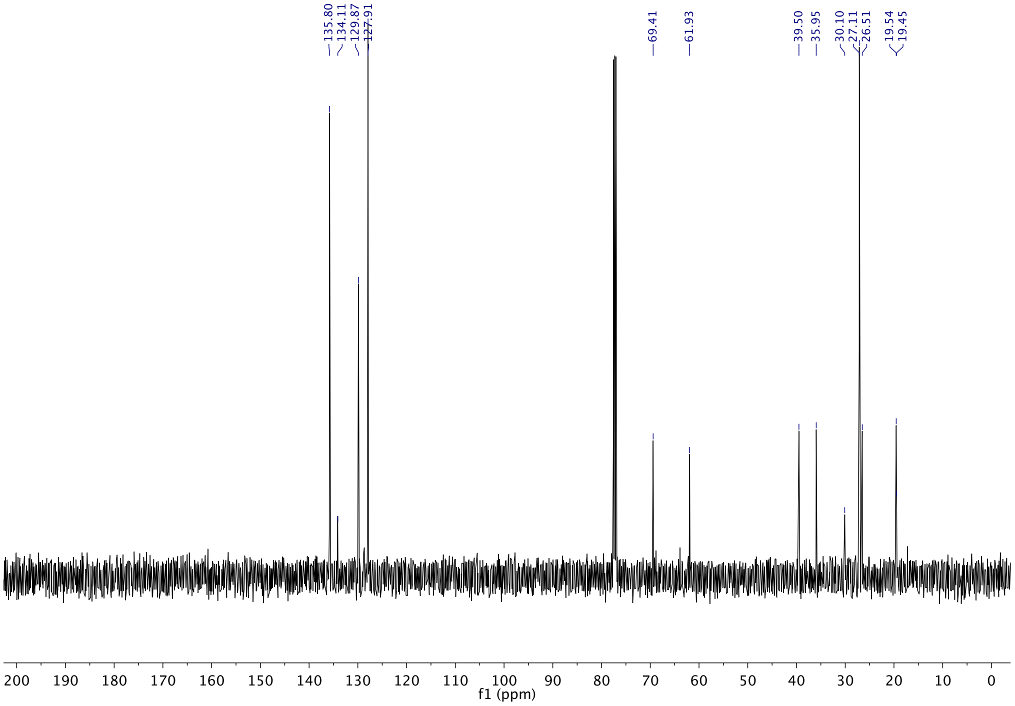
135.80
134.11
129.87
127.91
69.41
61.93
39.50
35.95
30.10
27.11
26.51
19.54
19.45
200 190 180 170 160 150 140 130 120 110 100 90 80 70 60 50 40 30 20 10 0
f1 (ppm)

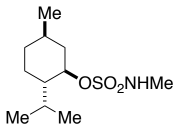
Me
OSO2NHMe
Me
Me

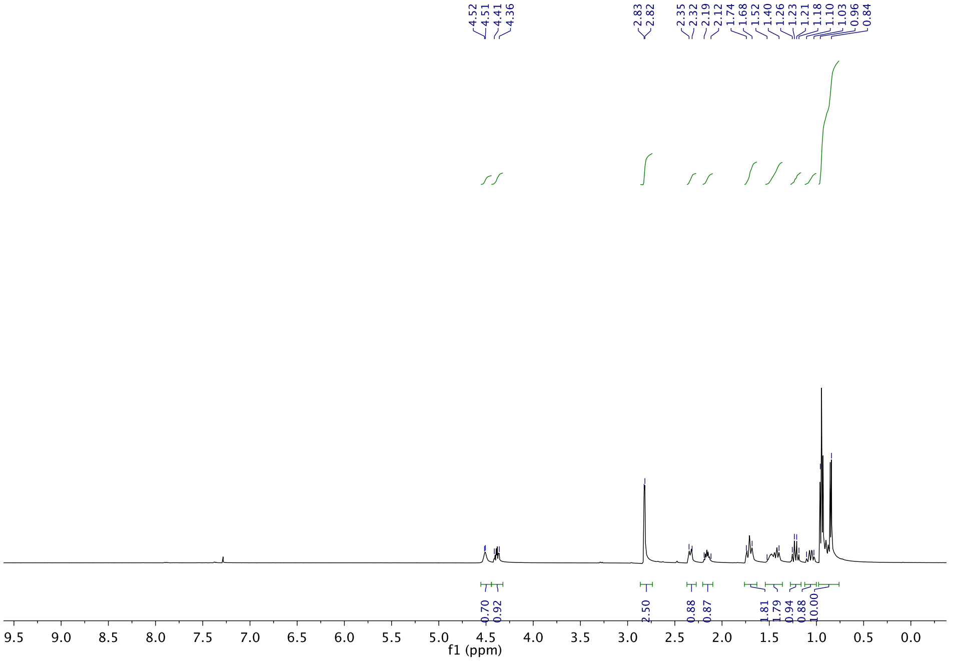
4.52
4.51
4.41
4.36
2.83
2.82
2.35
2.32
2.19
2.12
1.74
1.68
1.52
1.40
1.26
1.23
1.21
1.18
1.10
1.03
0.96
0.84
0.70
0.92
2.50
0.88
0.87
1.81
1.79
0.94
0.88
10.00
9.5 9.0 8.5 8.0 7.5 7.0 6.5 6.0 5.5 5.0 4.5 4.0 3.5 3.0 2.5 2.0 1.5 1.0 0.5 0.0
f1 (ppm)

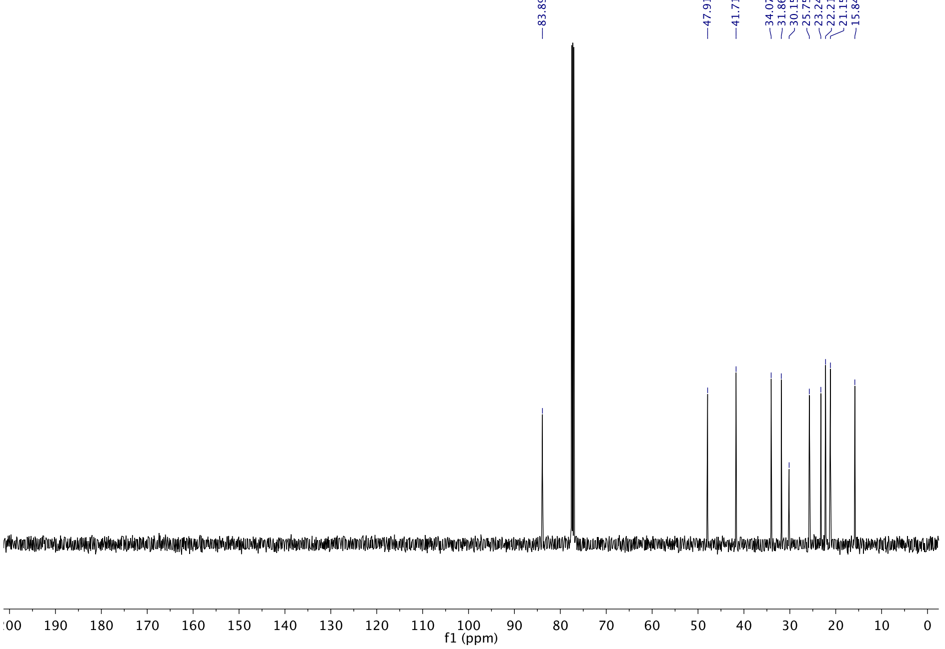
83.89
47.91
41.71
34.07
31.86
30.15
25.75
23.24
22.21
21.15
15.84
190 180 170 160 150 140 130 120 110 100 90 80 70 60 50 40 30 20 10 0
f1 (ppm)

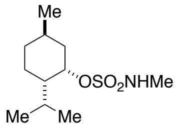

Me
OSO2NHMe
Me
Me

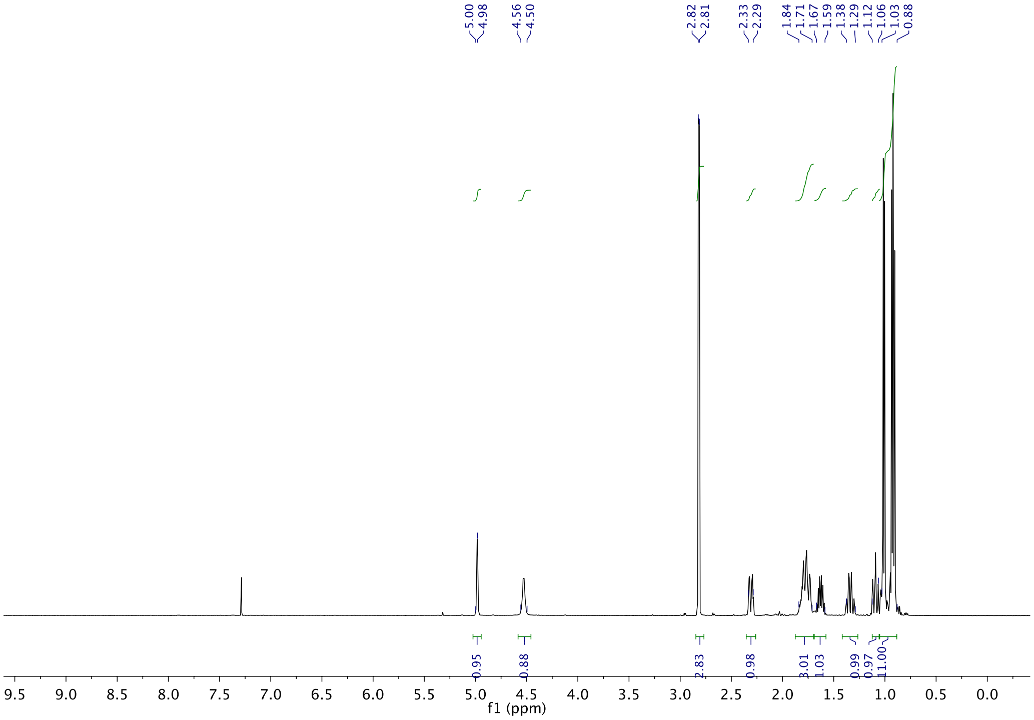

5.00
4.98
4.56
4.50
2.82
2.81
2.33
2.29
1.84
1.71
1.67
1.59
1.38
1.29
1.12
1.06
1.03
0.88
0.95
0.88
2.83
0.98
3.01
1.03
0.99
0.97
11.00
9.5 9.0 8.5 8.0 7.5 7.0 6.5 6.0 5.5 5.0 4.5 4.0 3.5 3.0 2.5 2.0 1.5 1.0 0.5 0.0
f1 (ppm)

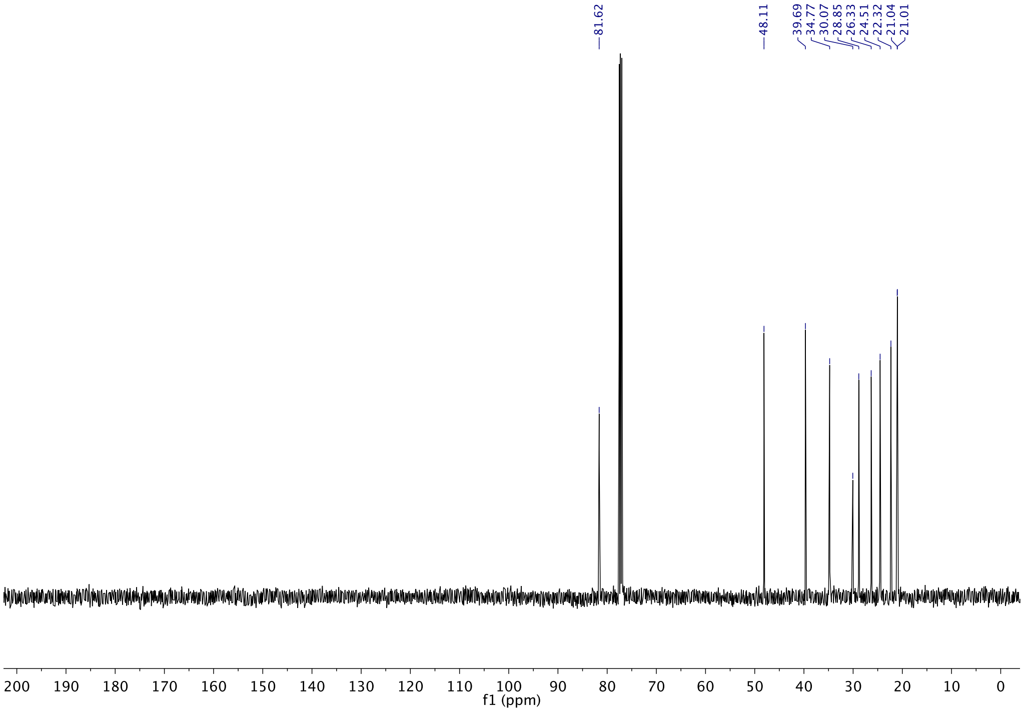

81.62
48.11
39.69
34.77
30.07
28.85
26.33
24.51
22.32
21.04
21.01
200 190 180 170 160 150 140 130 120 110 100 90 80 70 60 50 40 30 20 10 0
f1 (ppm)

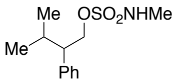
Me
OSO2NHMe
Me
Ph

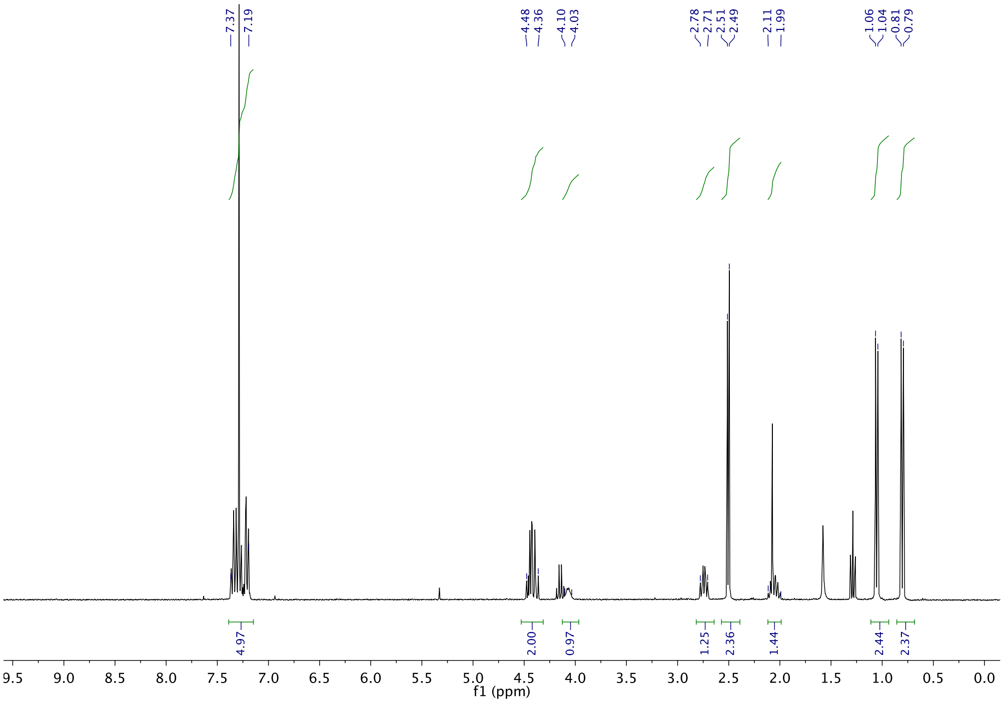
7.37
7.19
4.48
4.36
4.10
4.03
2.78
2.71
2.51
2.49
2.11
1.99
1.06
1.04
0.81
0.79
4.97
2.00
0.97
1.25
2.36
1.44
2.44
2.37
f1 (ppm)

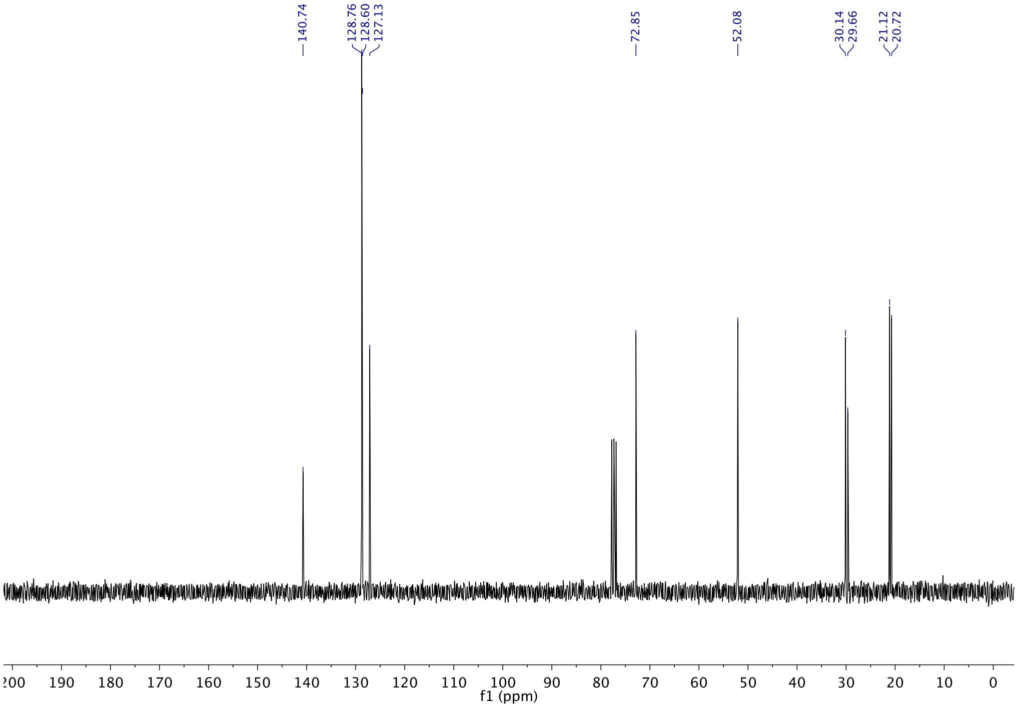
140.74
128.76
128.60
127.13
72.85
52.08
30.14
29.66
21.12
20.72
f1 (ppm)

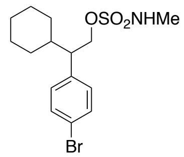
OSO2NHMe
Br

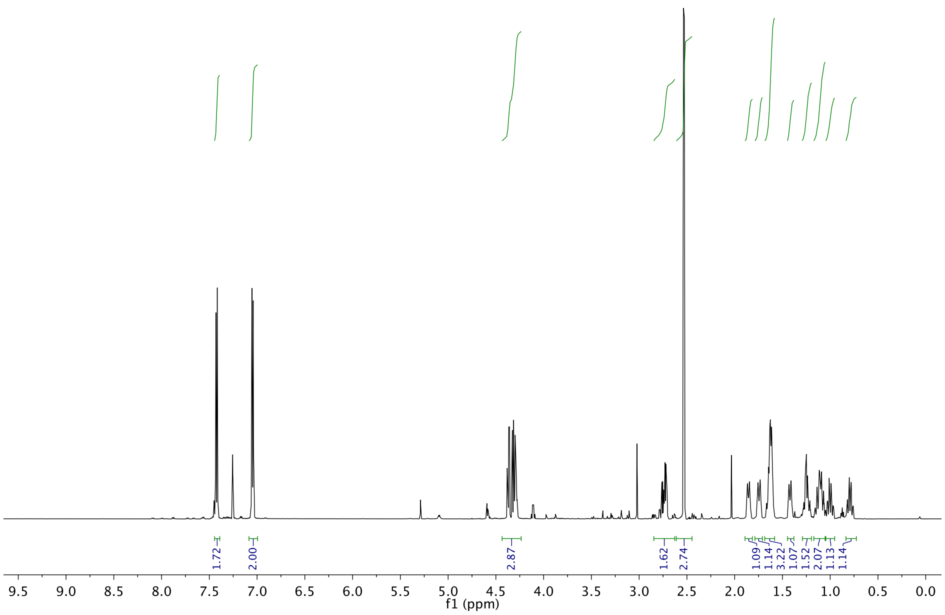
1.72
2.00
2.87
1.62
2.74
1.09
1.14
3.22
1.07
1.52
2.07
1.13
1.14
9.5 9.0 8.5 8.0 7.5 7.0 6.5 6.0 5.5 5.0 4.5 4.0 3.5 3.0 2.5 2.0 1.5 1.0 0.5 0.0
f1 (ppm)

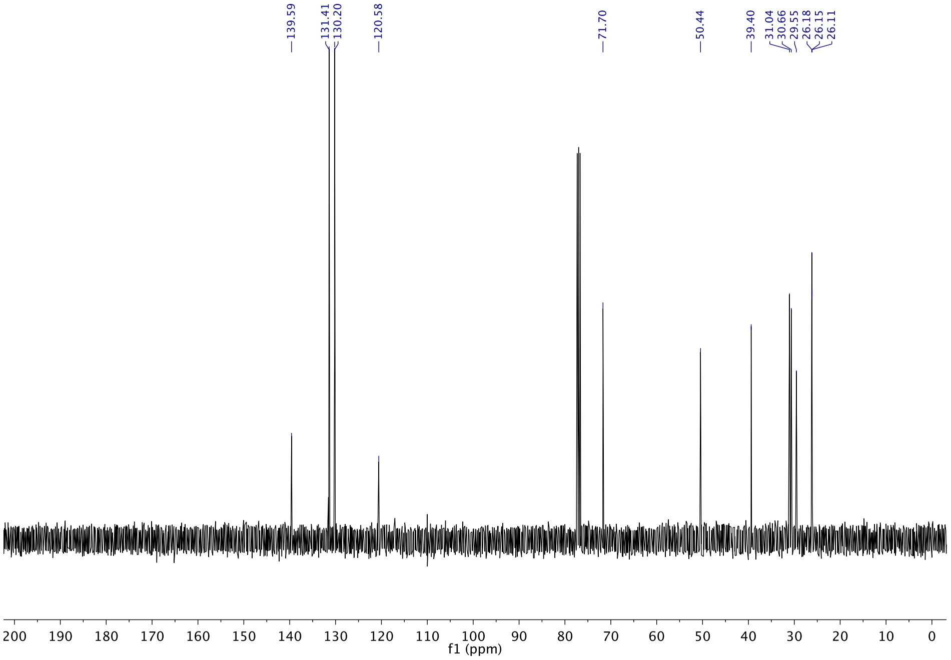
139.59
131.41
130.20
120.58
71.70
50.44
39.40
31.04
30.66
29.55
26.18
26.15
26.11
200 190 180 170 160 150 140 130 120 110 100 90 80 70 60 50 40 30 20 10 0
f1 (ppm)

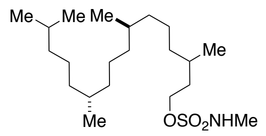
Me Me Me
Me
Me
OSO2NHMe

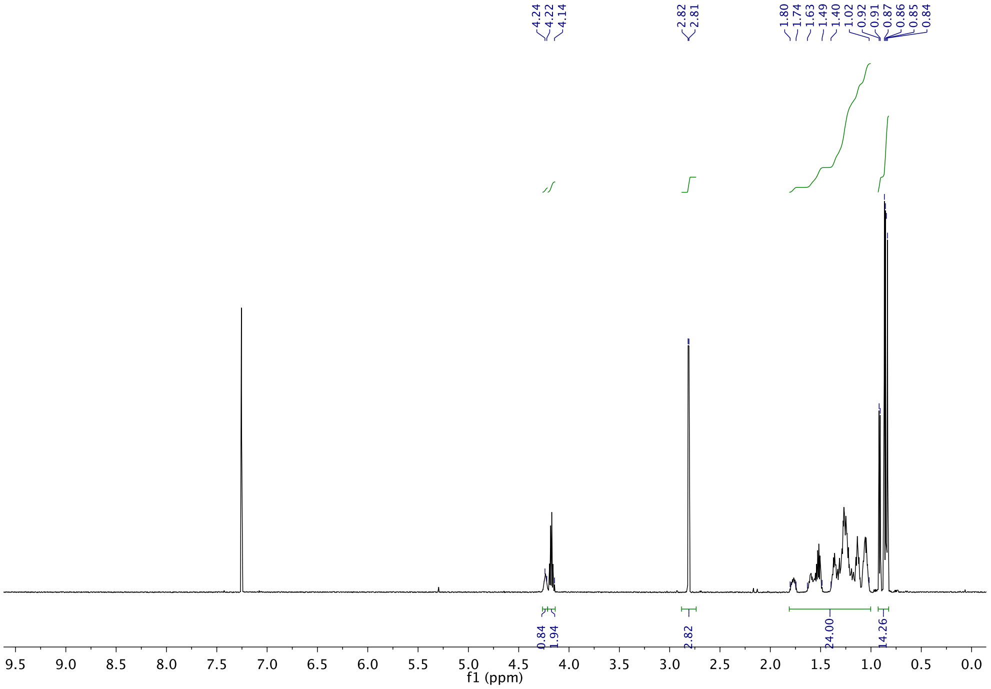
4.24
4.22
4.14
2.82
2.81
1.80
1.74
1.63
1.49
1.40
1.02
0.92
0.91
0.87
0.86
0.85
0.84
0.84
1.94
2.82
24.00
14.26
f1 (ppm)

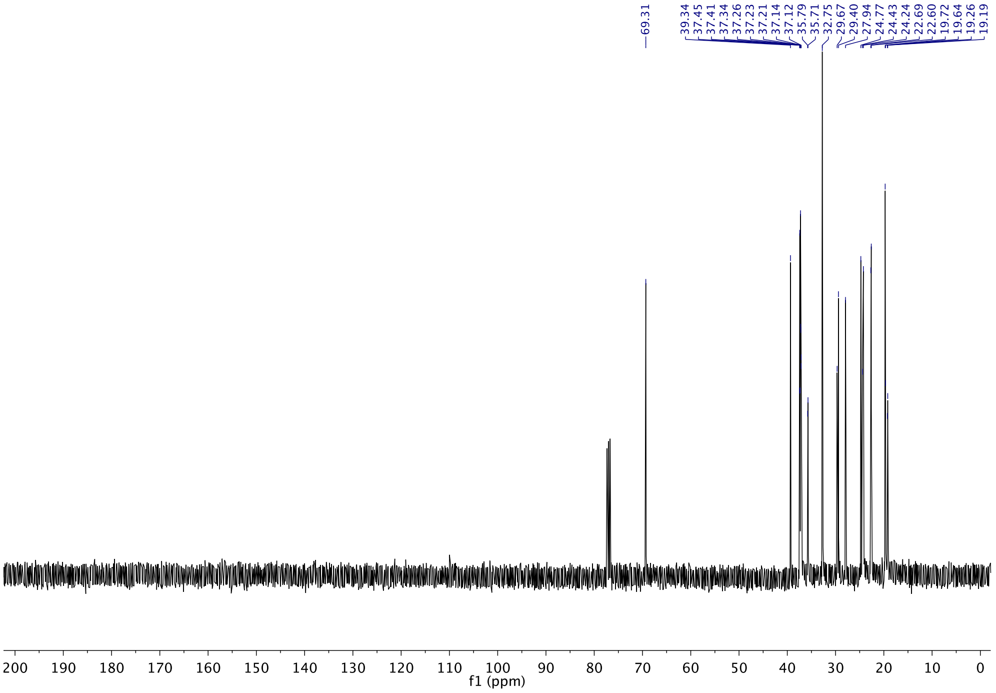
69.31
39.34
37.45
37.41
37.34
37.26
37.23
37.21
37.14
37.12
35.79
35.71
32.75
29.67
29.40
27.94
24.77
24.43
24.24
22.69
22.60
19.72
19.64
19.26
19.19
f1 (ppm)

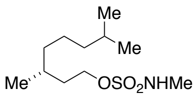
Me
Me
Me
OSO2NHMe

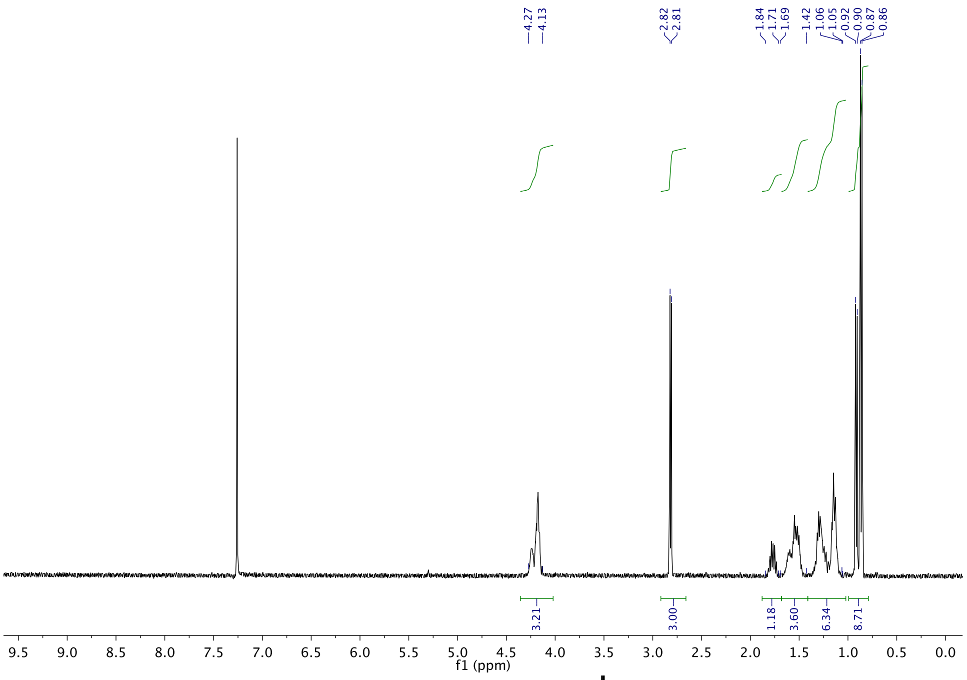
4.27
4.13
2.82
2.81
1.84
1.71
1.69
1.42
1.06
1.05
0.92
0.90
0.87
0.86
3.21
3.00
1.18
3.60
6.34
8.71
9.5
9.0
8.5
8.0
7.5
7.0
6.5
6.0
5.5
5.0
4.5
4.0
3.5
3.0
2.5
2.0
1.5
1.0
0.5
0.0
f1 (ppm)

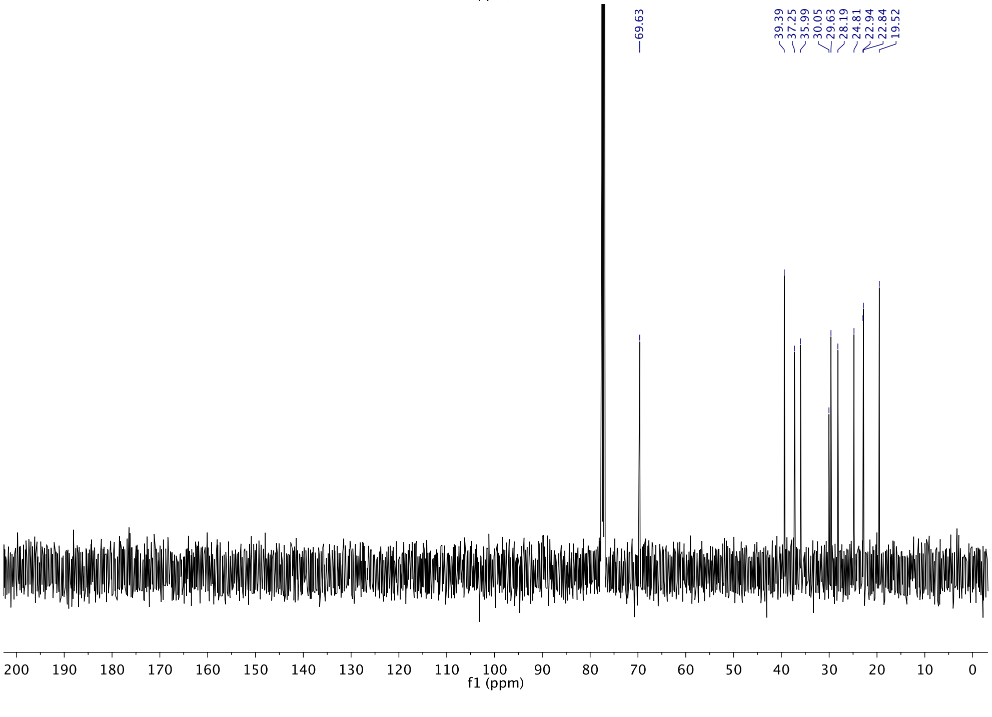
69.63
39.39
37.25
35.99
30.05
29.63
28.19
24.81
22.94
22.84
19.52
200
190
180
170
160
150
140
130
120
110
100
90
80
70
60
50
40
30
20
10
0
f1 (ppm)

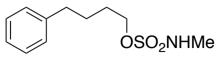
OSO2NHMe

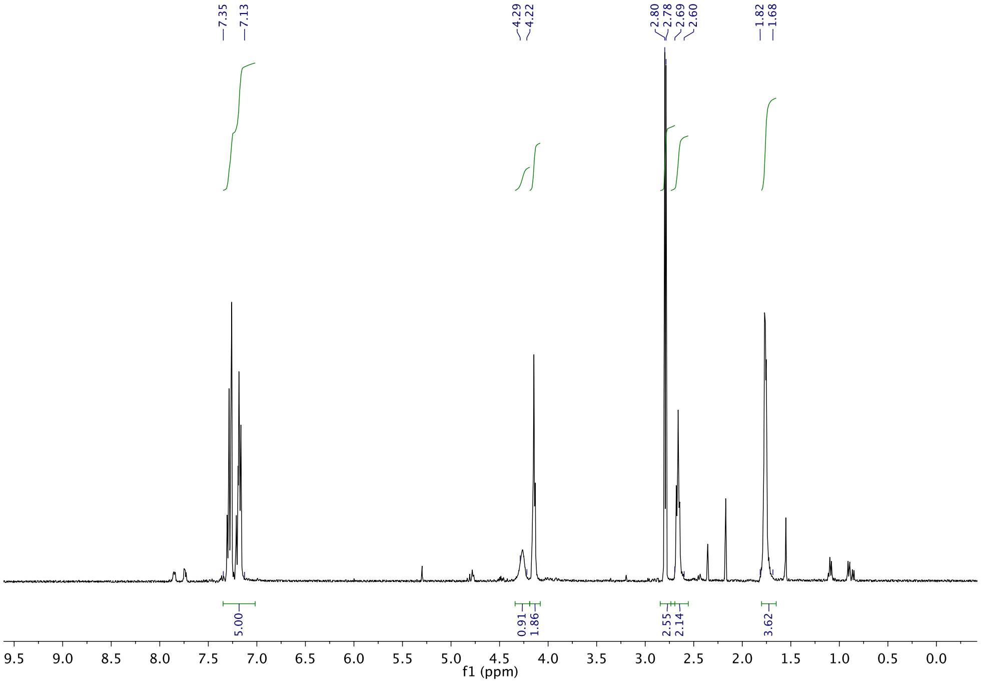
7.35
7.13
4.29
4.22
2.80
2.78
2.69
2.60
1.82
1.68
5.00
0.91
1.86
2.55
2.14
3.62
9.5 9.0 8.5 8.0 7.5 7.0 6.5 6.0 5.5 5.0 4.5 4.0 3.5 3.0 2.5 2.0 1.5 1.0 0.5 0.0
f1 (ppm)

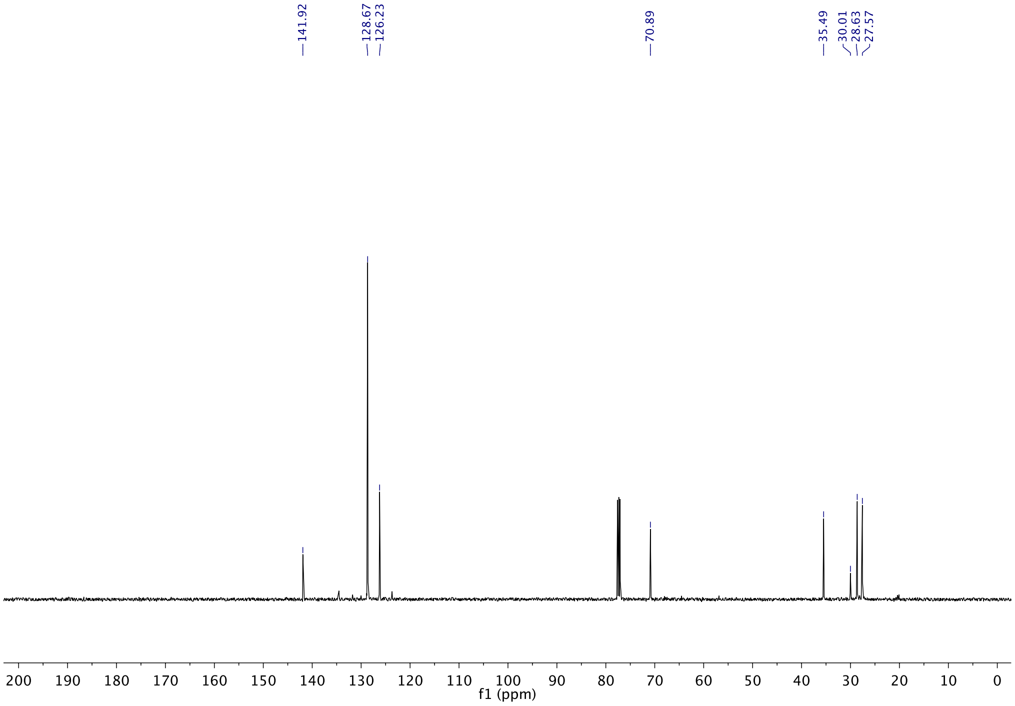
141.92
128.67
126.23
70.89
35.49
30.01
28.63
27.57
200 190 180 170 160 150 140 130 120 110 100 90 80 70 60 50 40 30 20 10 0
f1 (ppm)

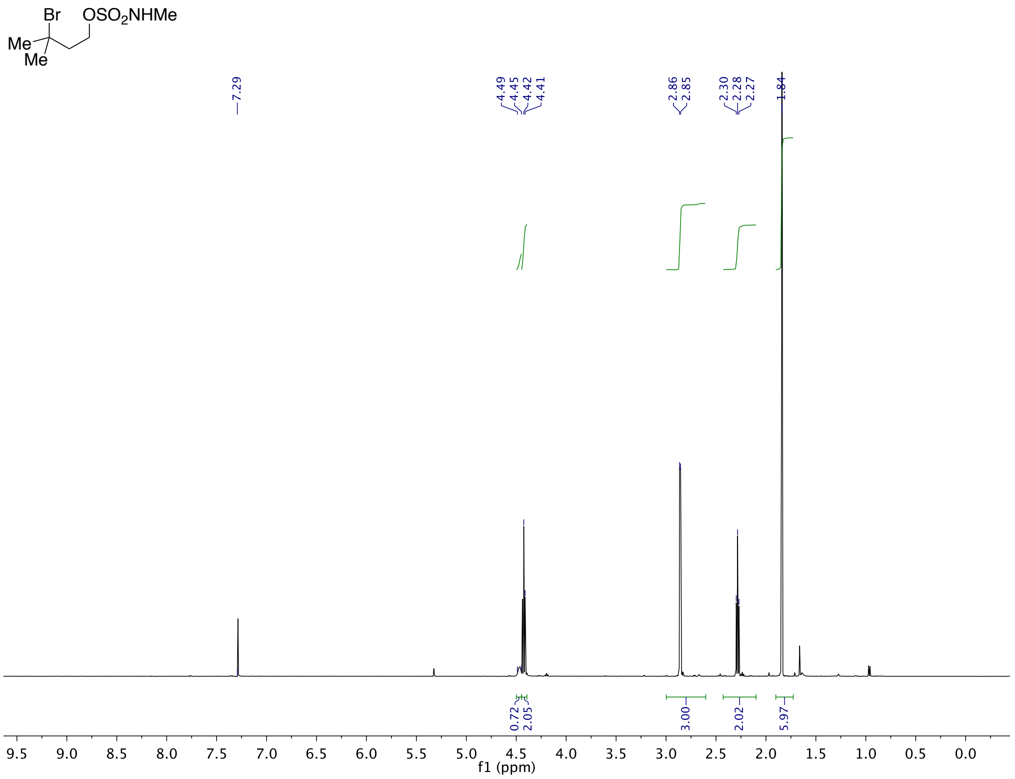
Br
OSO2NHMe
Me
Me
7.29
4.49
4.45
4.42
4.41
2.86
2.85
2.30
2.28
2.27
1.84
0.72
2.05
3.00
2.02
5.97
f1 (ppm)

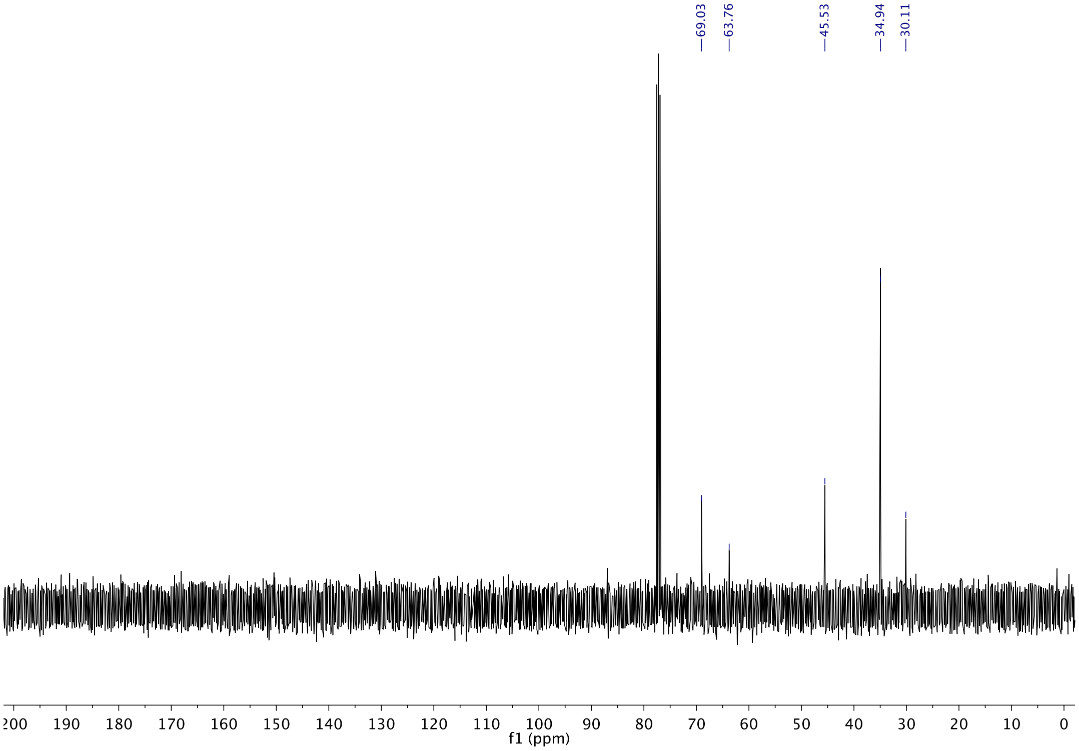
69.03
63.76
45.53
34.94
30.11
f1 (ppm)

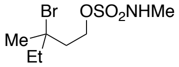
Br
OSO2NHMe
Me
Et

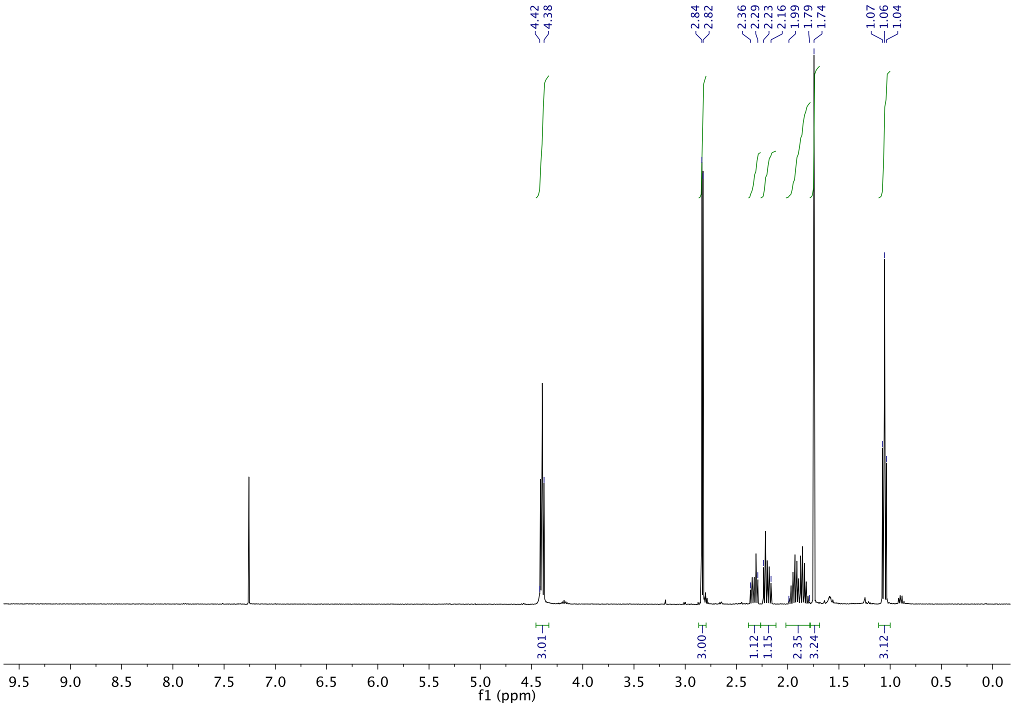
4.42
4.38
2.84
2.82
2.36
2.29
2.23
2.16
1.99
1.79
1.74
1.07
1.06
1.04
3.01
3.00
1.12
1.15
2.35
3.24
3.12
9.5 9.0 8.5 8.0 7.5 7.0 6.5 6.0 5.5 5.0 4.5 4.0 3.5 3.0 2.5 2.0 1.5 1.0 0.5 0.0
f1 (ppm)

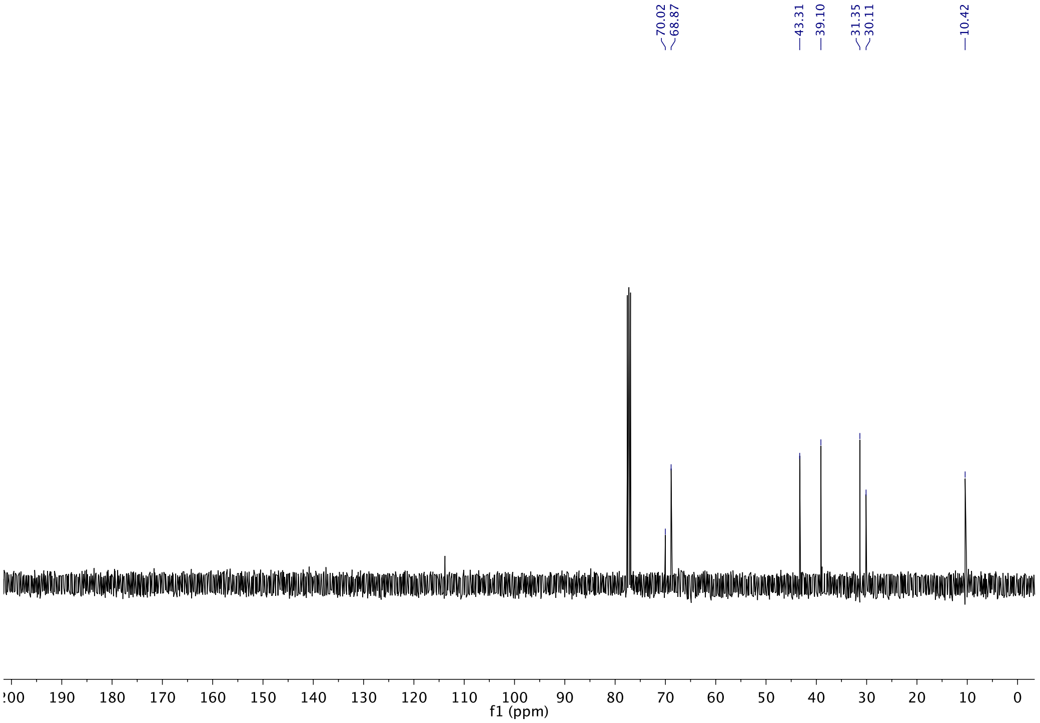
70.02
68.87
43.31
39.10
31.35
30.11
10.42
200 190 180 170 160 150 140 130 120 110 100 90 80 70 60 50 40 30 20 10 0
f1 (ppm)

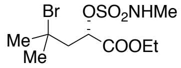
Br
OSO2NHMe
Me
Me
COOEt

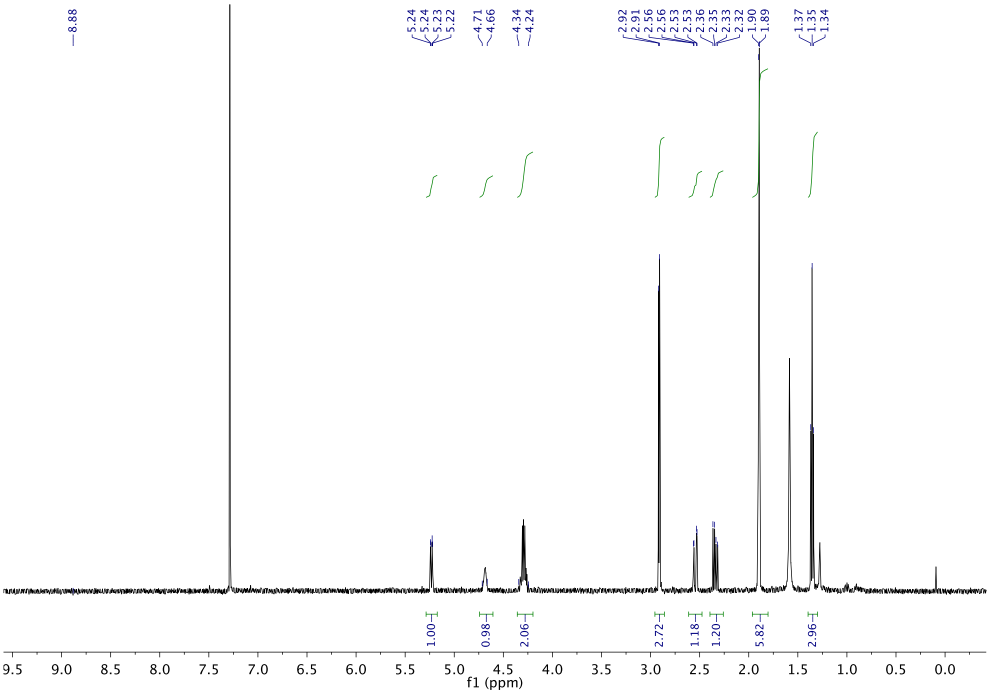
8.88
5.24
5.24
5.23
5.22
4.71
4.66
4.34
4.24
2.92
2.91
2.56
2.56
2.53
2.53
2.36
2.35
2.33
2.32
1.90
1.89
1.37
1.35
1.34
1.00
0.98
2.06
2.72
1.18
1.20
5.82
2.96
9.5 9.0 8.5 8.0 7.5 7.0 6.5 6.0 5.5 5.0 4.5 4.0 3.5 3.0 2.5 2.0 1.5 1.0 0.5 0.0
f1 (ppm)

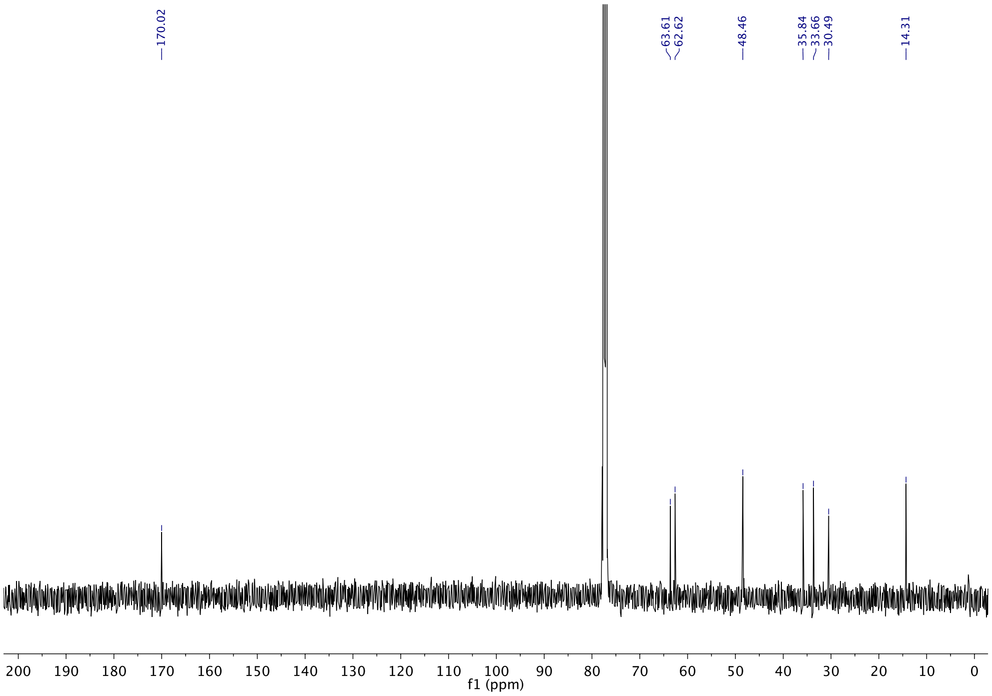
170.02
63.61
62.62
48.46
35.84
33.66
30.49
14.31
200 190 180 170 160 150 140 130 120 110 100 90 80 70 60 50 40 30 20 10 0
f1 (ppm)

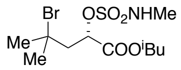

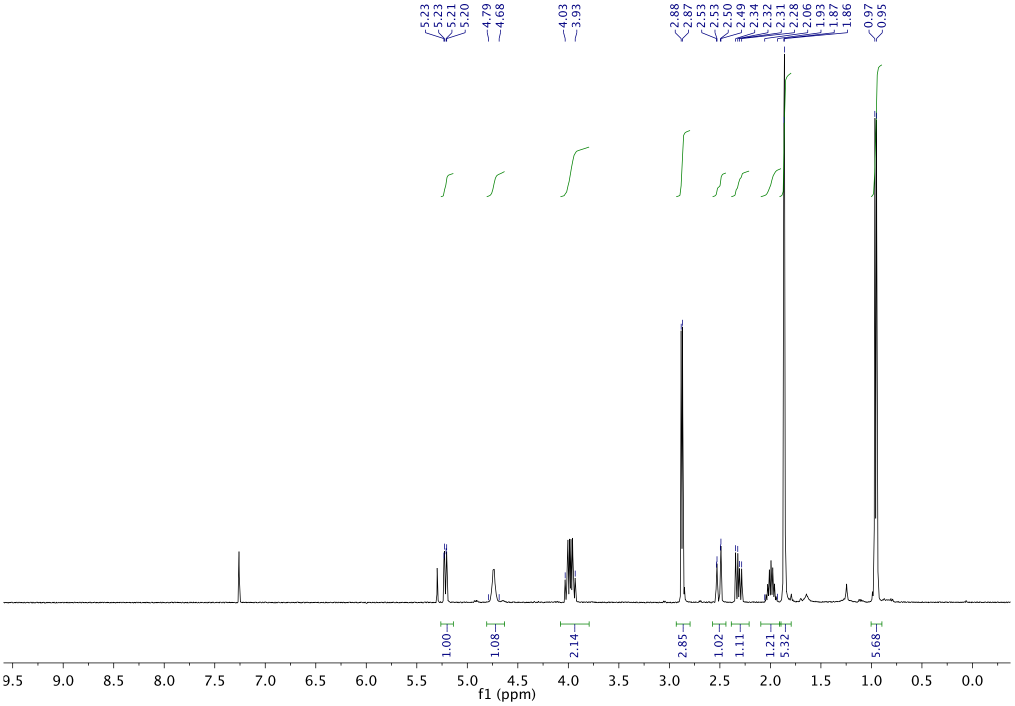
5.23
5.23
5.21
5.20
4.79
4.68
4.03
3.93
2.88
2.87
2.53
2.53
2.50
2.49
2.34
2.32
2.31
2.28
2.06
1.93
1.87
1.86
0.97
0.95
1.00
1.08
2.14
2.85
1.02
1.11
1.21
5.32
5.68
f1 (ppm)

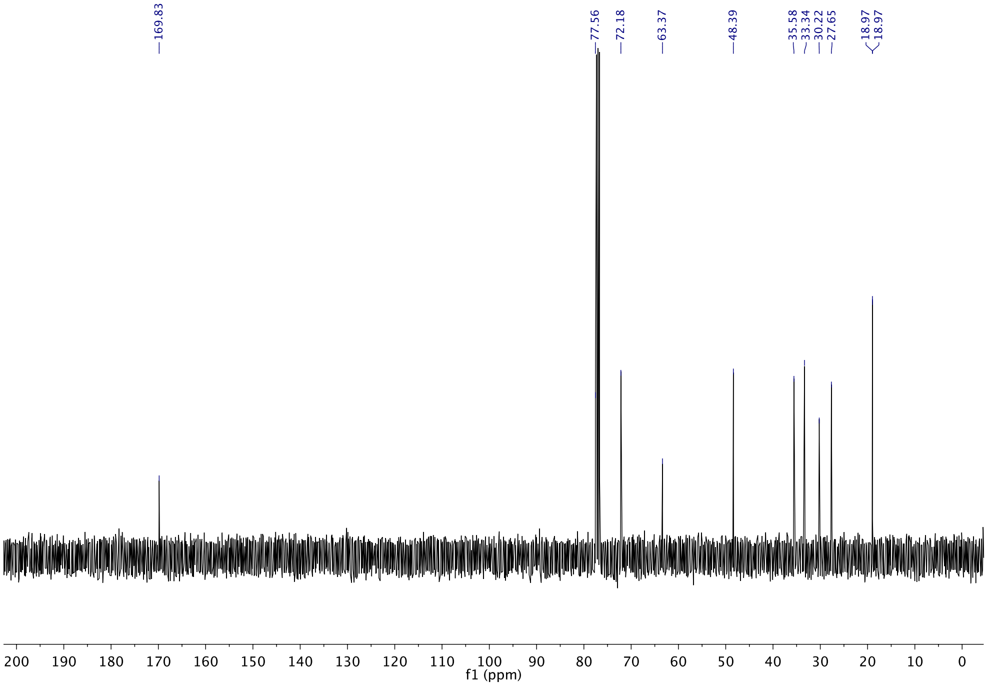
169.83
77.56
72.18
63.37
48.39
35.58
33.34
30.22
27.65
18.97
18.97
f1 (ppm)

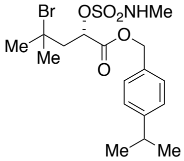
Br
OSO2NHMe
Me
Me
O
O
Me
Me

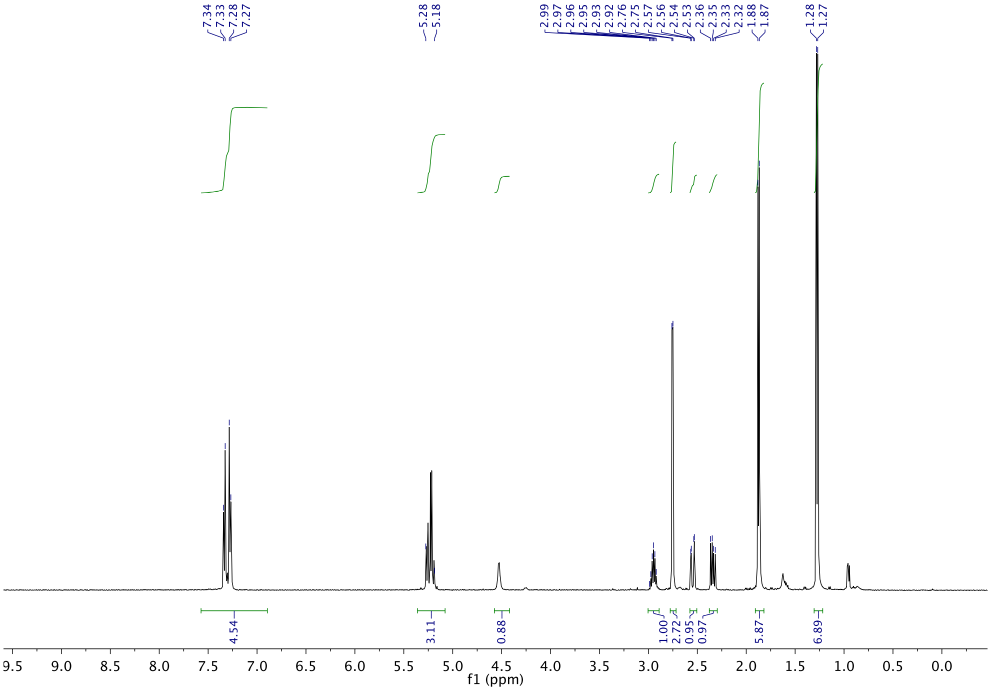
7.34
7.33
7.28
7.27
5.28
5.18
2.99
2.97
2.96
2.95
2.93
2.92
2.76
2.75
2.57
2.56
2.54
2.53
2.36
2.35
2.33
2.32
1.88
1.87
1.28
1.27
4.54
3.11
0.88
1.00
2.72
0.95
0.97
5.87
6.89
9.5 9.0 8.5 8.0 7.5 7.0 6.5 6.0 5.5 5.0 4.5 4.0 3.5 3.0 2.5 2.0 1.5 1.0 0.5 0.0
f1 (ppm)

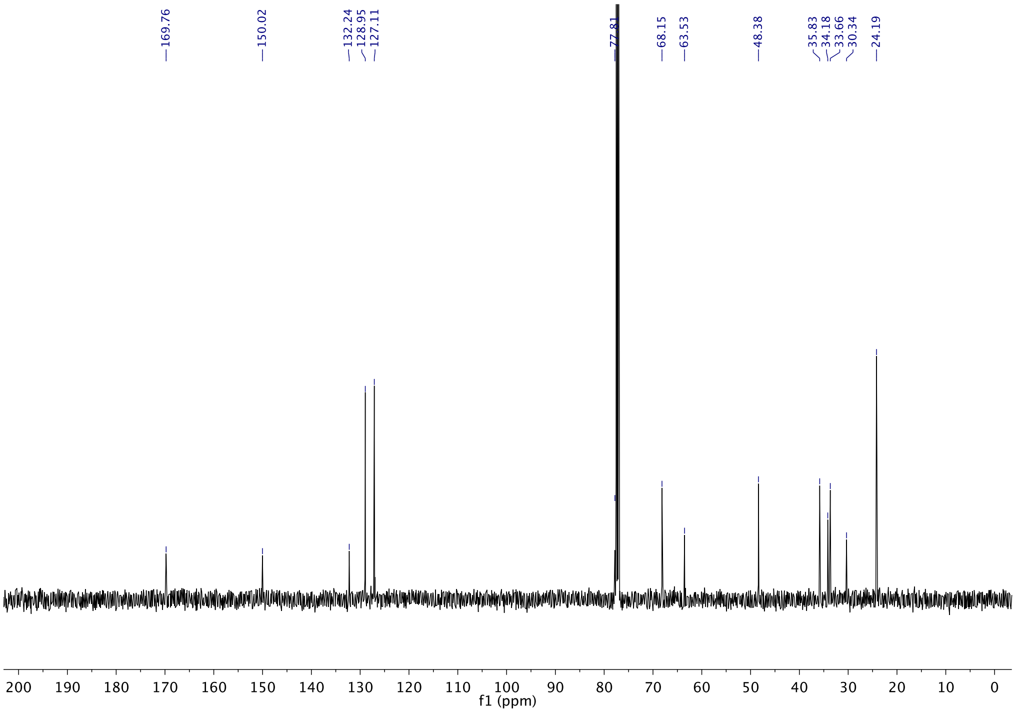
169.76
150.02
132.24
128.95
127.11
77.81
68.15
63.53
48.38
35.83
34.18
33.66
30.34
24.19
200 190 180 170 160 150 140 130 120 110 100 90 80 70 60 50 40 30 20 10 0
f1 (ppm)

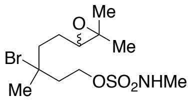
Me
O
Me
Br
Me
OSO2NHMe

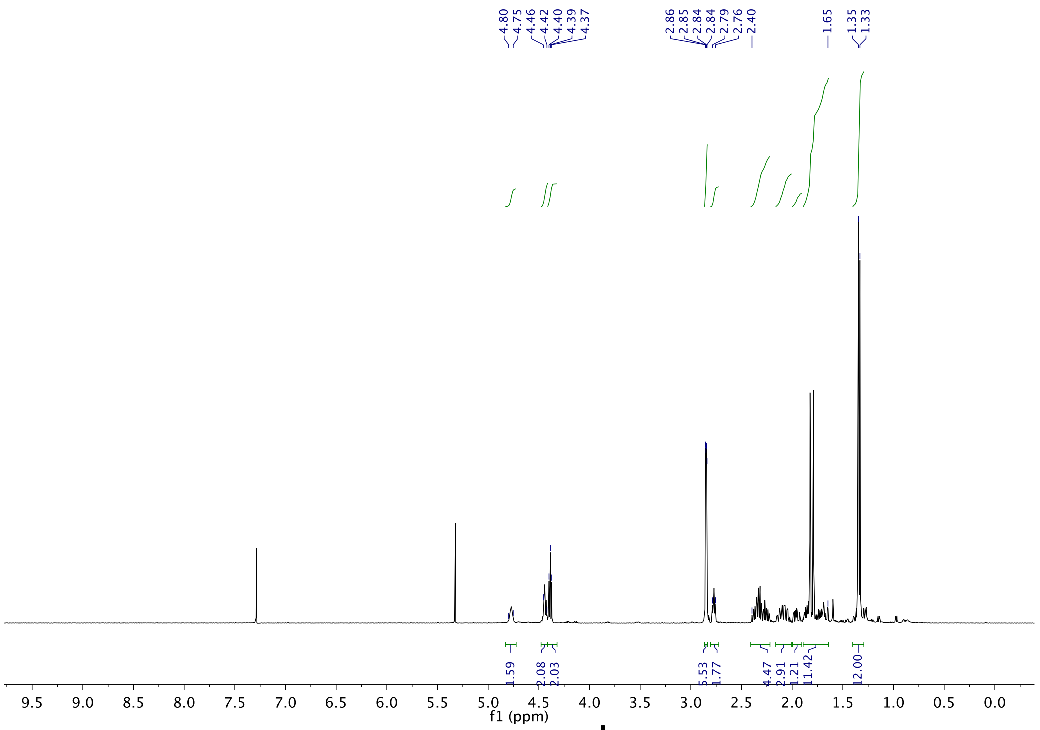

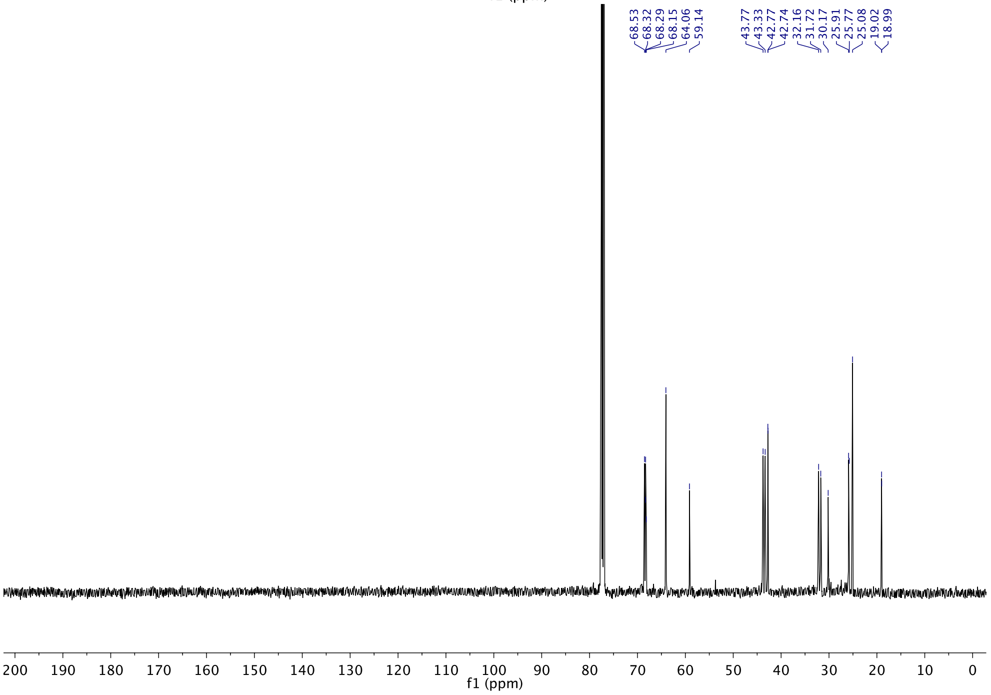

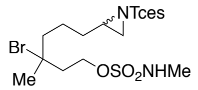
NTces
Br
Me
OSO2NHMe

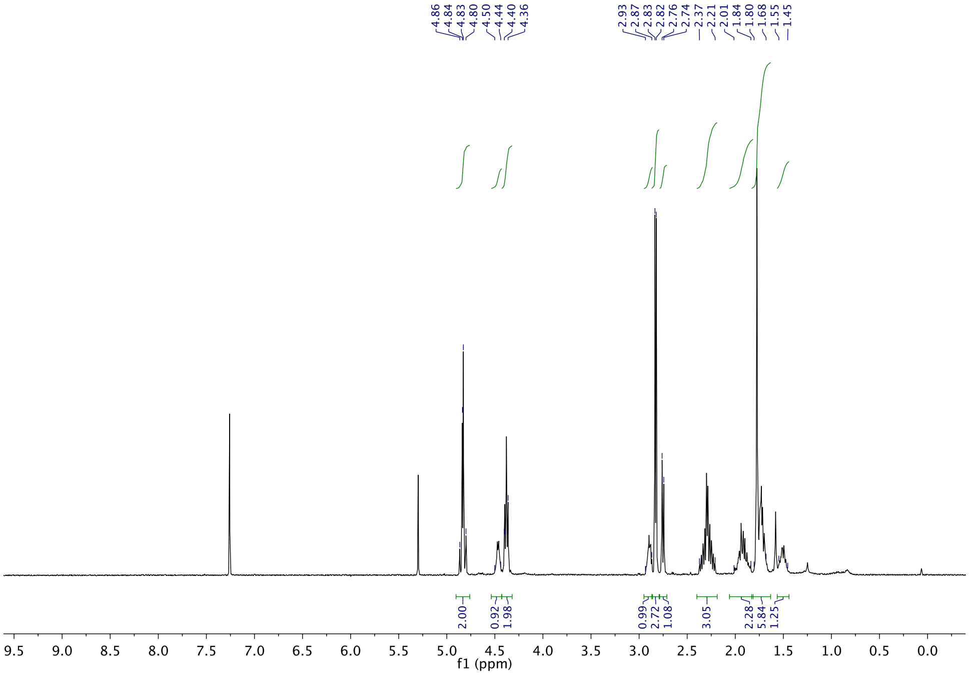
4.86
4.84
4.83
4.80
4.50
4.44
4.40
4.36
2.93
2.87
2.83
2.82
2.76
2.74
2.37
2.21
2.01
1.84
1.80
1.68
1.55
1.45
2.00
0.92
1.98
0.99
2.72
1.08
3.05
2.28
5.84
1.25
9.5 9.0 8.5 8.0 7.5 7.0 6.5 6.0 5.5 5.0 4.5 4.0 3.5 3.0 2.5 2.0 1.5 1.0 0.5 0.0
f1 (ppm)

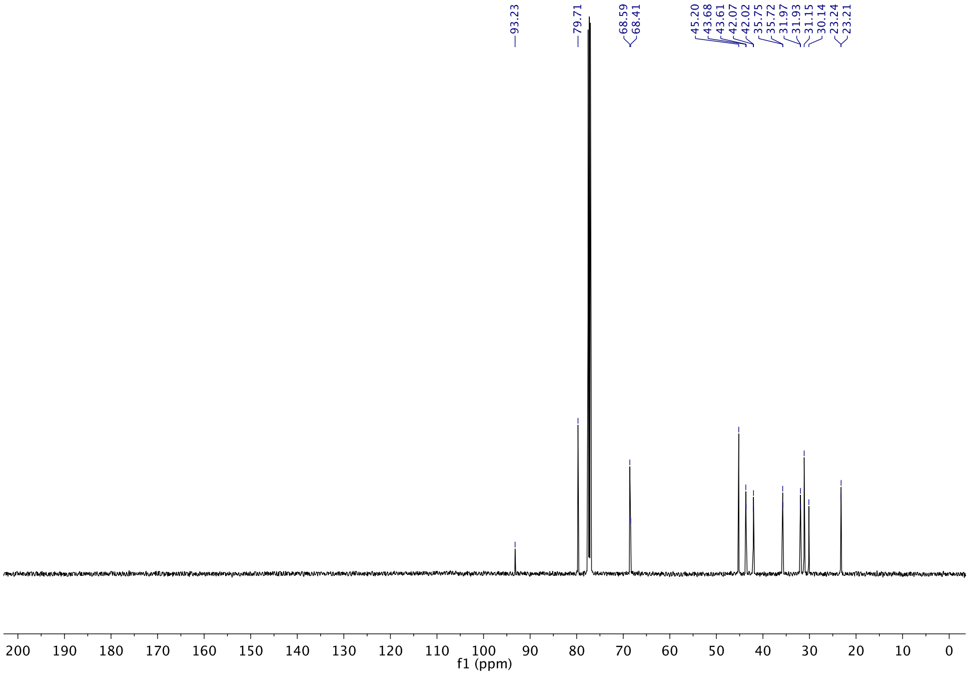
93.23
79.71
68.59
68.41
45.20
43.68
43.61
42.07
42.02
35.75
35.72
31.97
31.93
31.15
30.14
23.24
23.21
200 190 180 170 160 150 140 130 120 110 100 90 80 70 60 50 40 30 20 10 0
f1 (ppm)

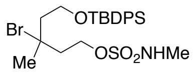
Br
OTBDPS
Me
OSO2NHMe

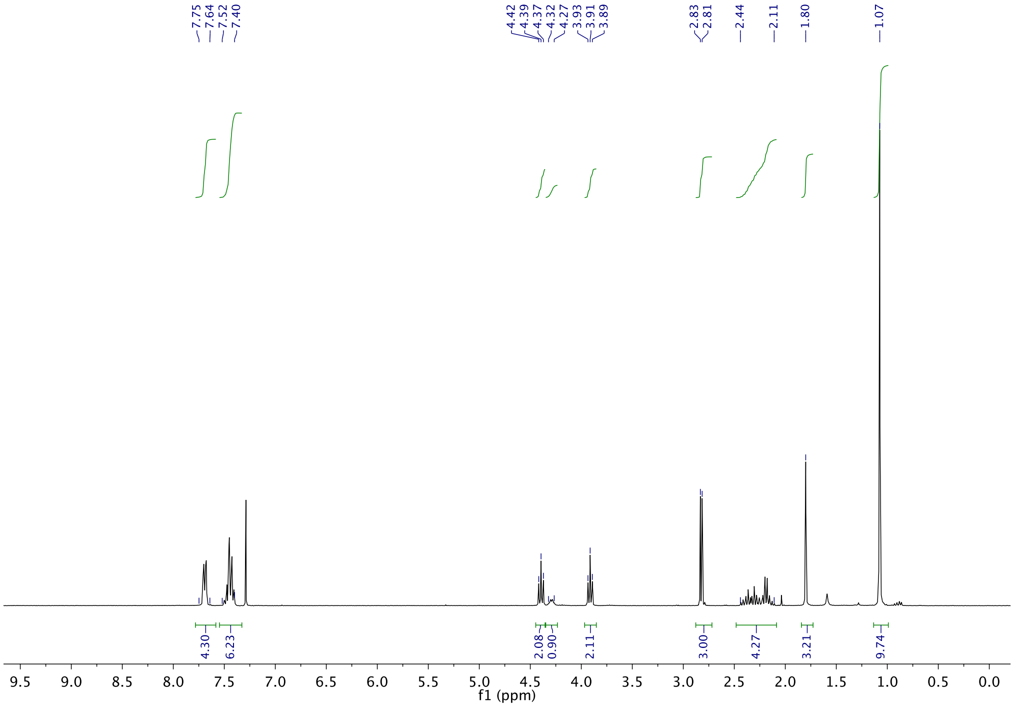

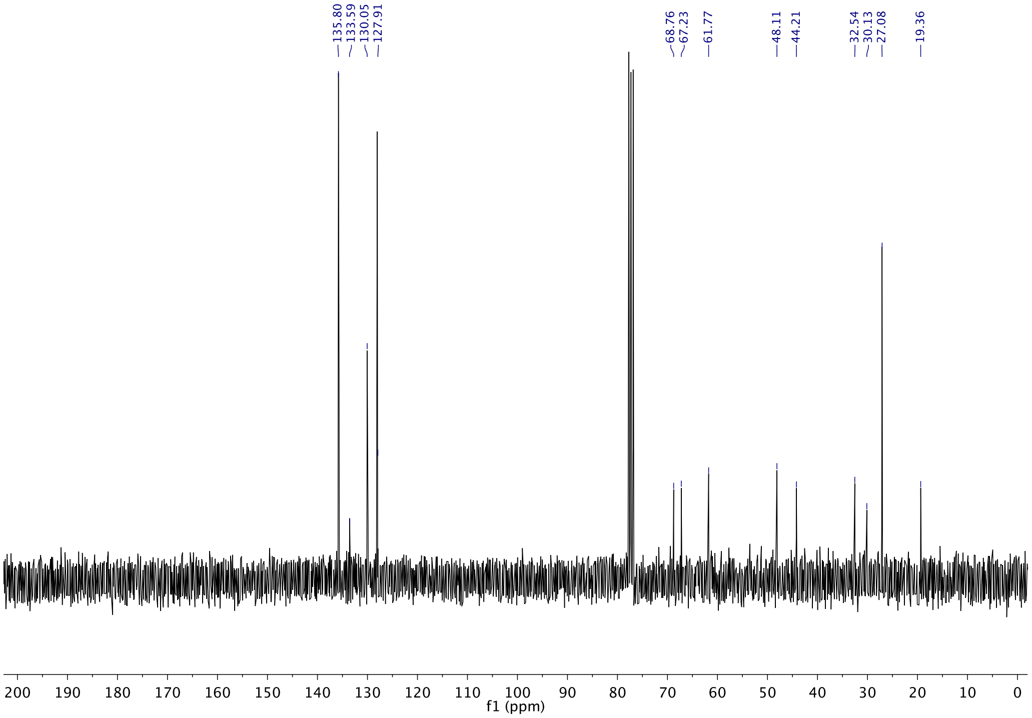

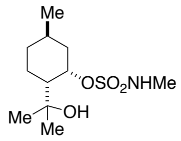
Me
OSO2NHMe
Me
OH
Me

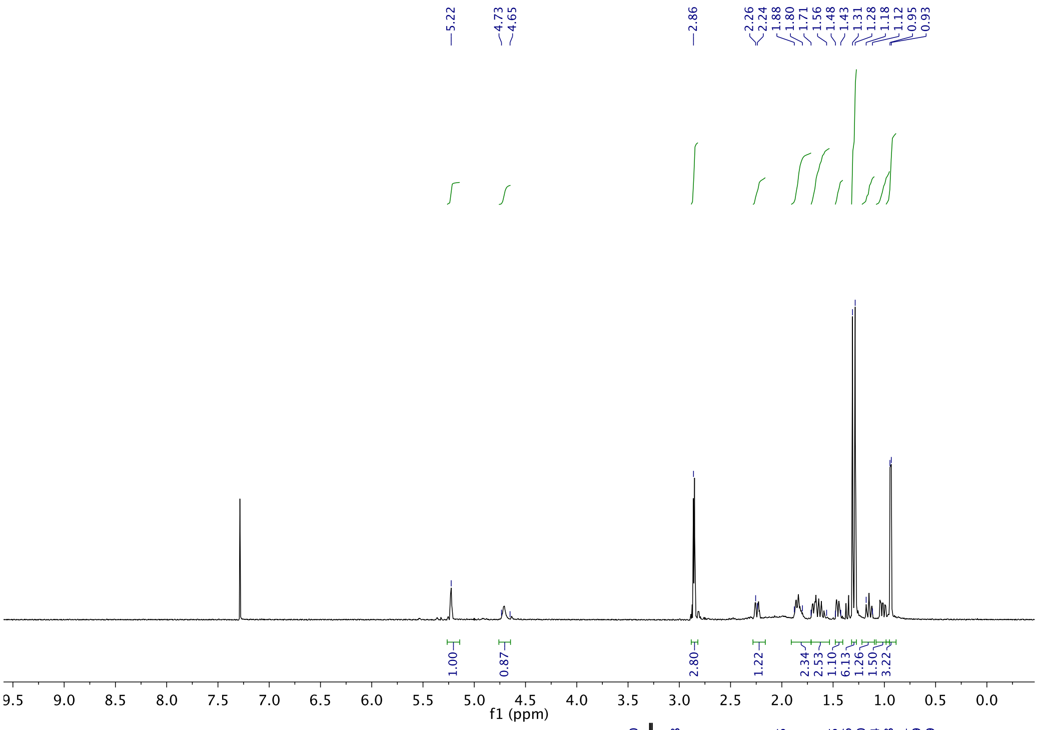
5.22
4.73
4.65
2.86
2.26
2.24
1.88
1.80
1.71
1.56
1.48
1.43
1.31
1.28
1.18
1.12
0.95
0.93
1.00
0.87
2.80
1.22
2.34
2.53
1.10
6.13
1.26
1.50
3.22
9.5 9.0 8.5 8.0 7.5 7.0 6.5 6.0 5.5 5.0 4.5 4.0 3.5 3.0 2.5 2.0 1.5 1.0 0.5 0.0
f1 (ppm)

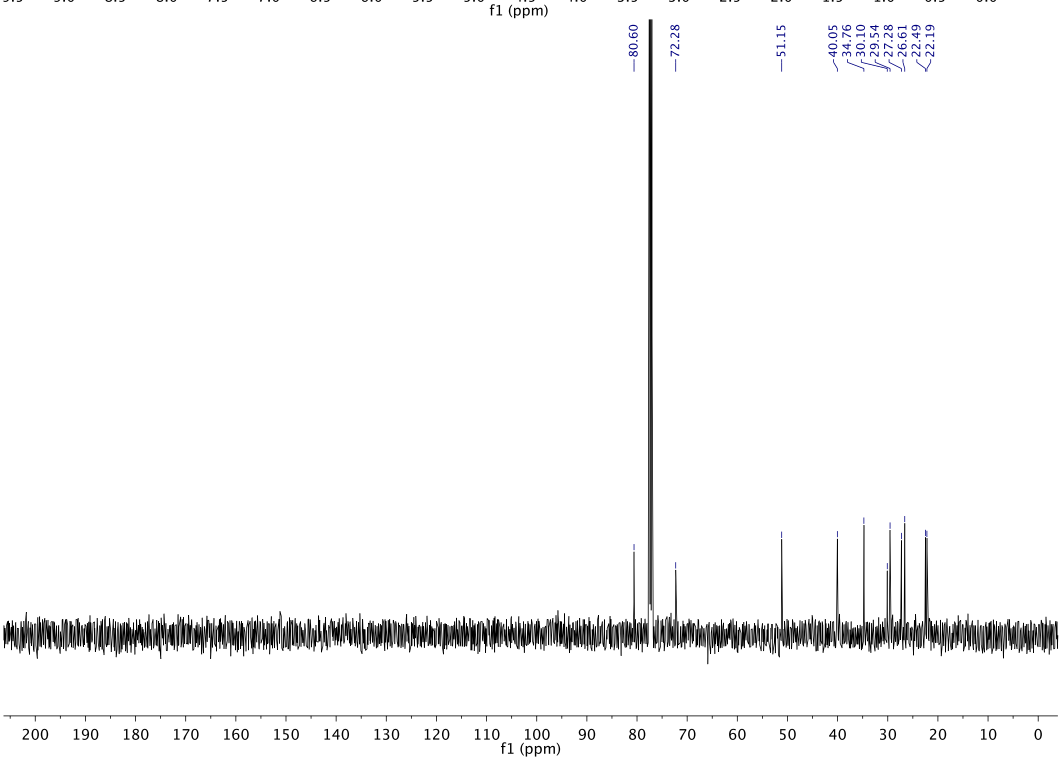
80.60
72.28
51.15
40.05
34.76
30.10
29.54
27.28
26.61
22.49
22.19
200 190 180 170 160 150 140 130 120 110 100 90 80 70 60 50 40 30 20 10 0
f1 (ppm)

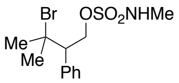
Br
OSO2NHMe
Me
Me
Ph

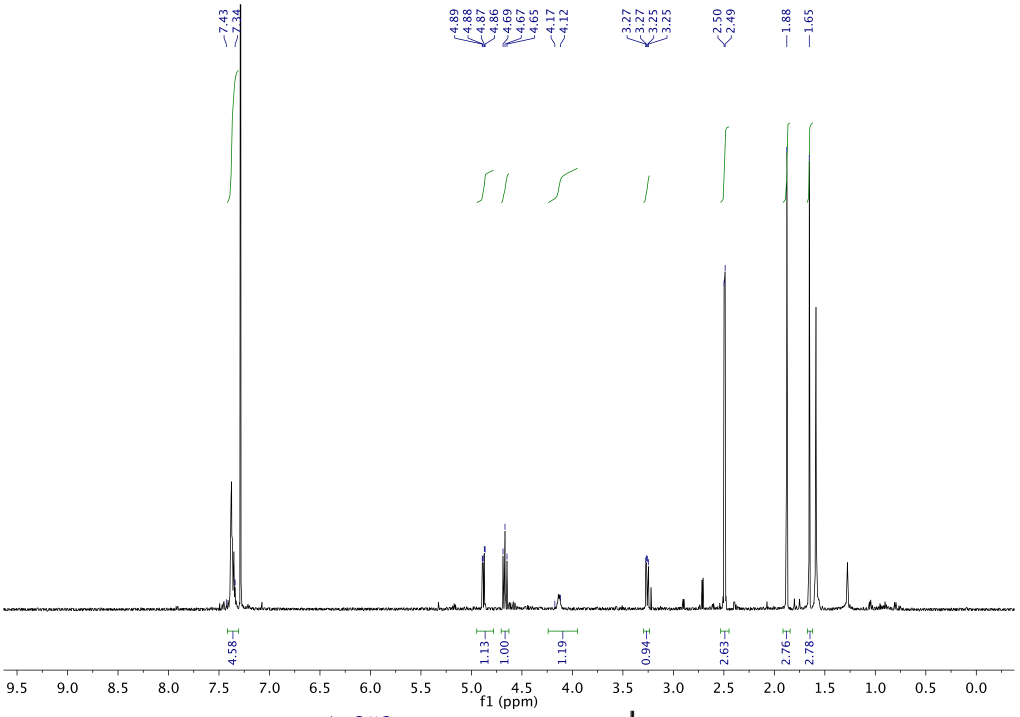
7.43
7.34
4.89
4.88
4.87
4.86
4.69
4.67
4.65
4.17
4.12
3.27
3.27
3.25
3.25
2.50
2.49
1.88
1.65
4.58
1.13
1.00
1.19
0.94
2.63
2.76
2.78
f1 (ppm)

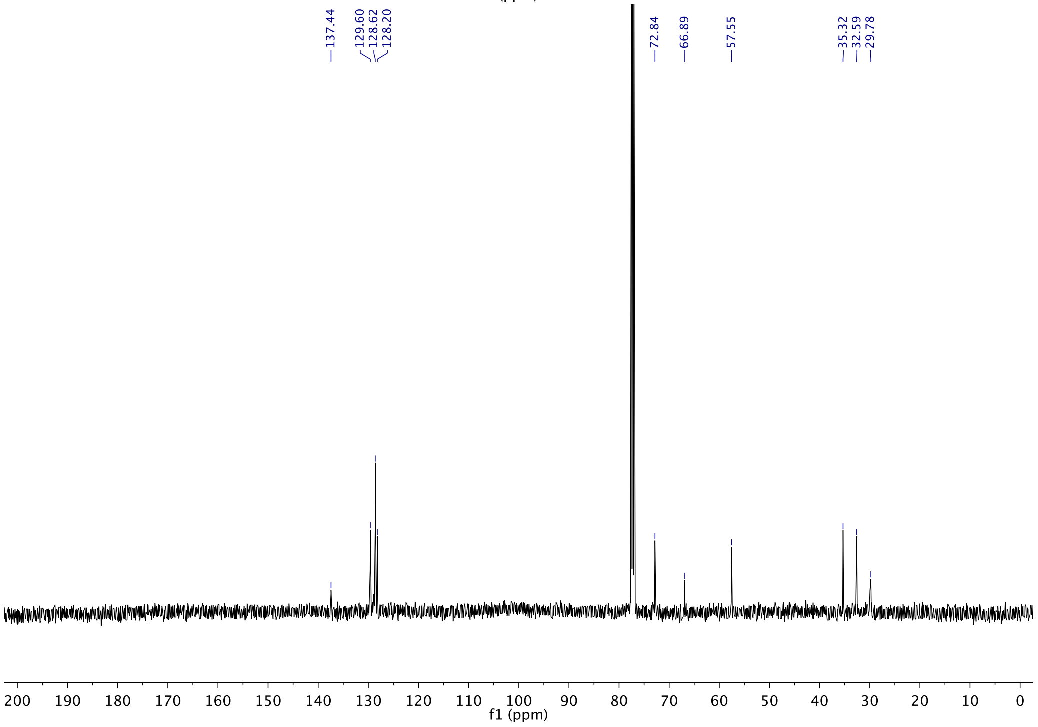
137.44
129.60
128.62
128.20
72.84
66.89
57.55
35.32
32.59
29.78
f1 (ppm)

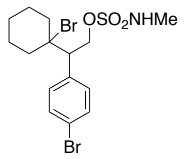
Br
OSO2NHMe
Br

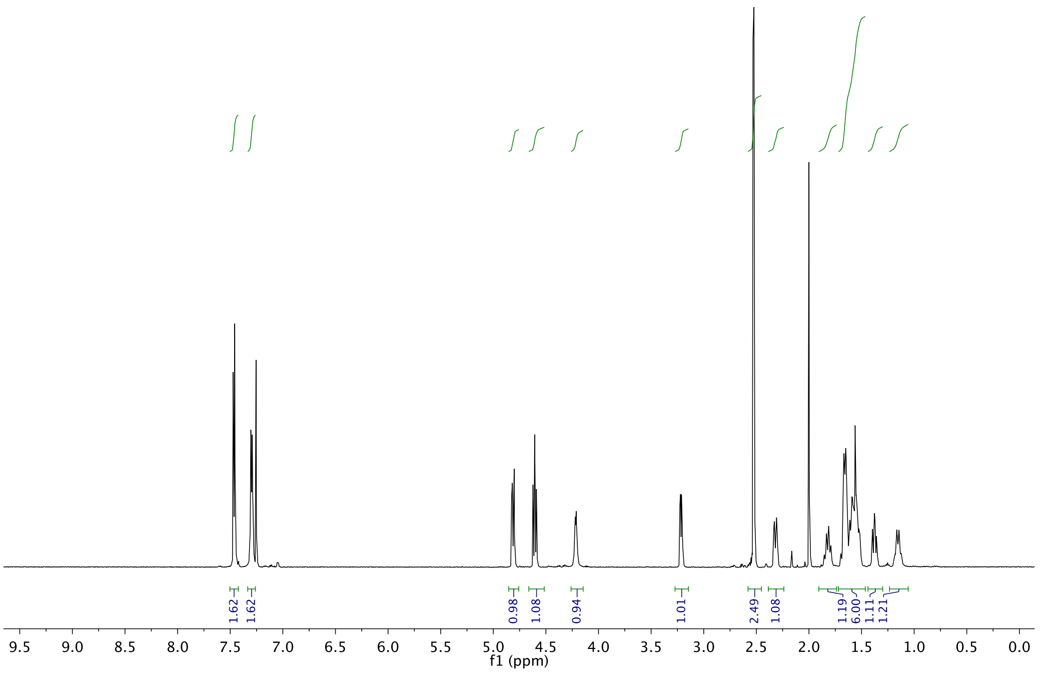
1.62
1.62
0.98
1.08
0.94
1.01
2.49
1.08
1.19
6.00
1.11
1.21
f1 (ppm)

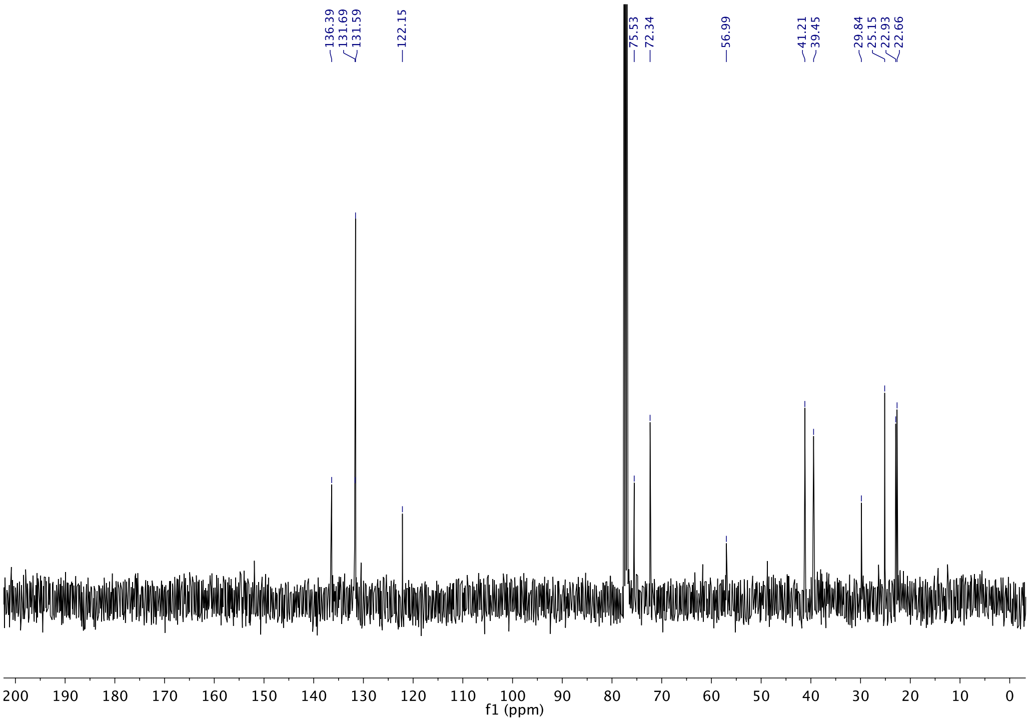
136.39
131.69
131.59
122.15
75.53
72.34
56.99
41.21
39.45
29.84
25.15
22.93
22.66
f1 (ppm)

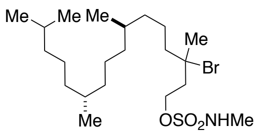
Me
Me Me
Me
Br
Me
OSO2NHMe

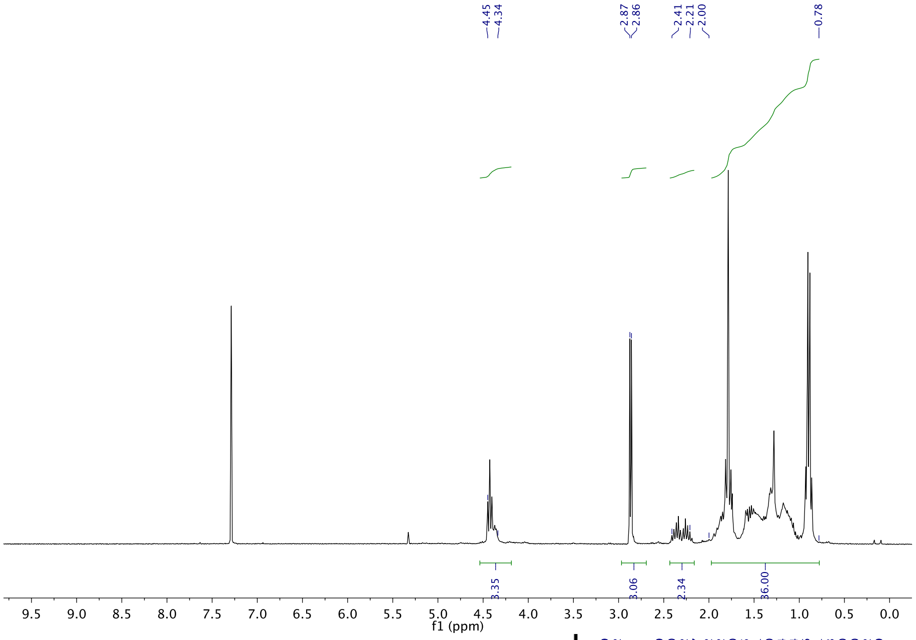
4.45
4.34
2.87
2.86
2.41
2.21
2.00
0.78
3.35
3.06
2.34
36.00
9.5 9.0 8.5 8.0 7.5 7.0 6.5 6.0 5.5 5.0 4.5 4.0 3.5 3.0 2.5 2.0 1.5 1.0 0.5 0.0
f1 (ppm)

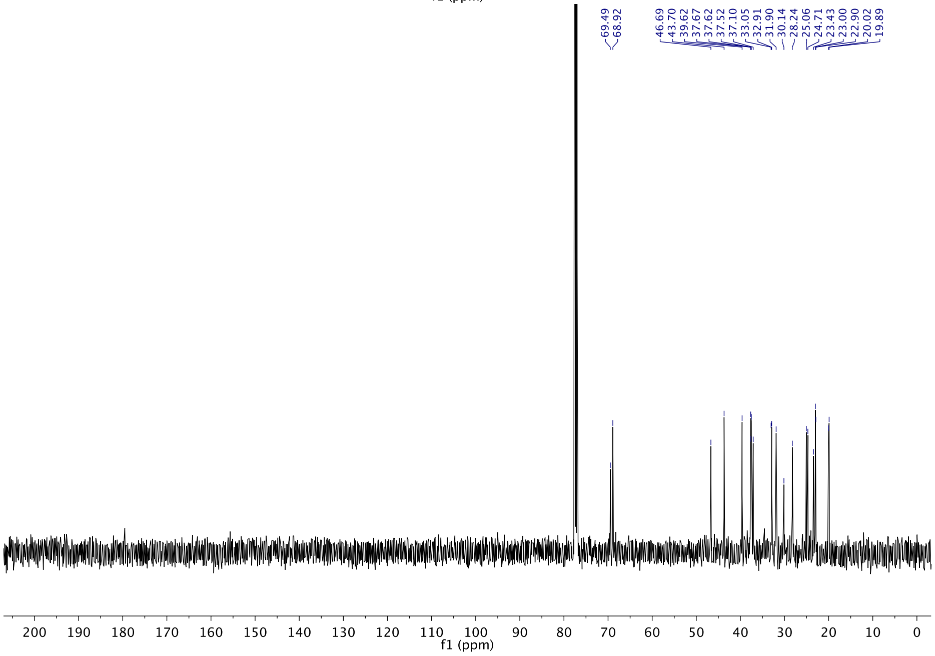
69.49
68.92
46.69
43.70
39.62
37.67
37.62
37.52
37.10
33.05
32.91
31.90
30.14
28.24
25.06
24.71
23.43
23.00
22.90
20.02
19.89
200 190 180 170 160 150 140 130 120 110 100 90 80 70 60 50 40 30 20 10 0
f1 (ppm)

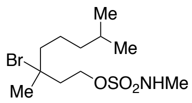
Me
Me
Br
Me
OSO2NHMe

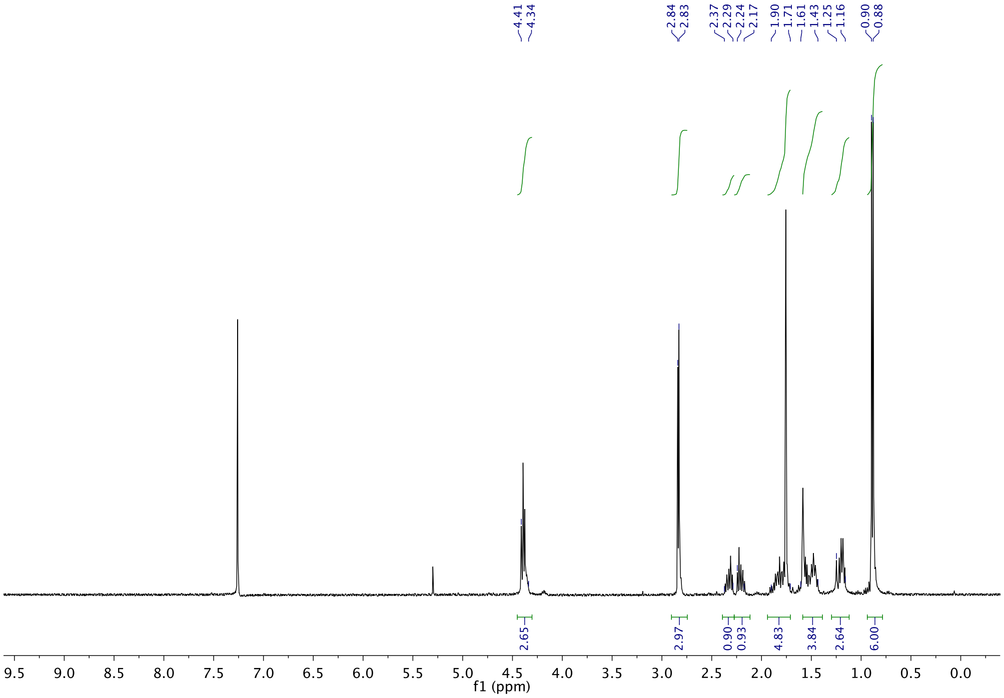
4.41
4.34
2.84
2.83
2.37
2.29
2.24
2.17
1.90
1.71
1.61
1.43
1.25
1.16
0.90
0.88
2.65
2.97
0.90
0.93
4.83
3.84
2.64
6.00
9.5 9.0 8.5 8.0 7.5 7.0 6.5 6.0 5.5 5.0 4.5 4.0 3.5 3.0 2.5 2.0 1.5 1.0 0.5 0.0
f1 (ppm)

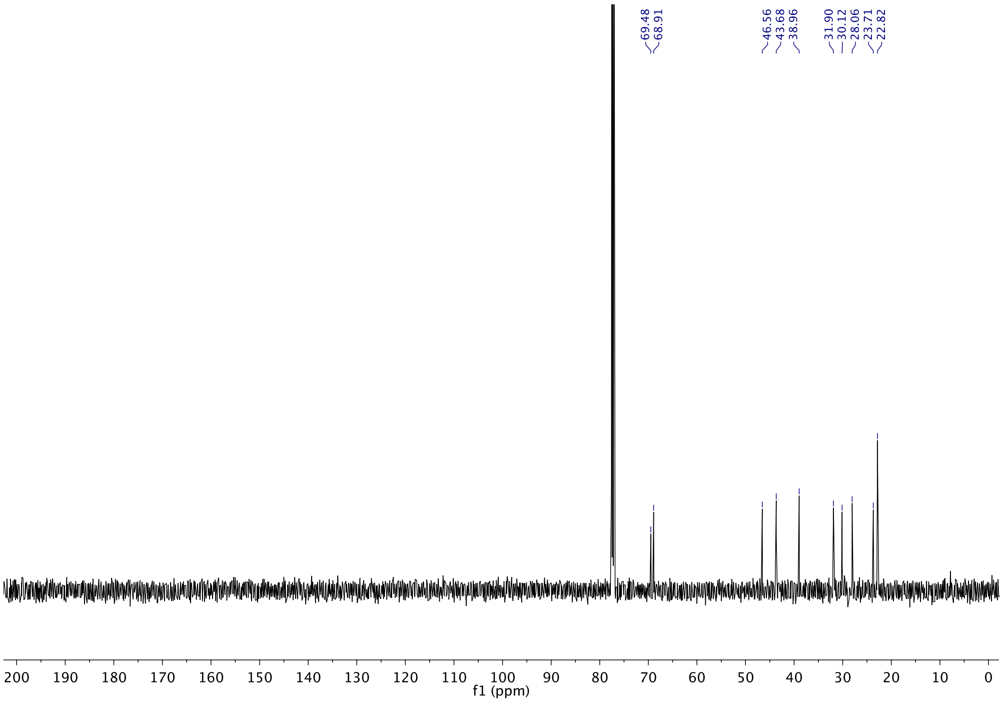
69.48
68.91
46.56
43.68
38.96
31.90
30.12
28.06
23.71
22.82
200 190 180 170 160 150 140 130 120 110 100 90 80 70 60 50 40 30 20 10 0
f1 (ppm)

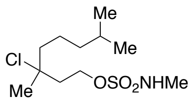
Me
Me
Cl
Me
OSO2NHMe

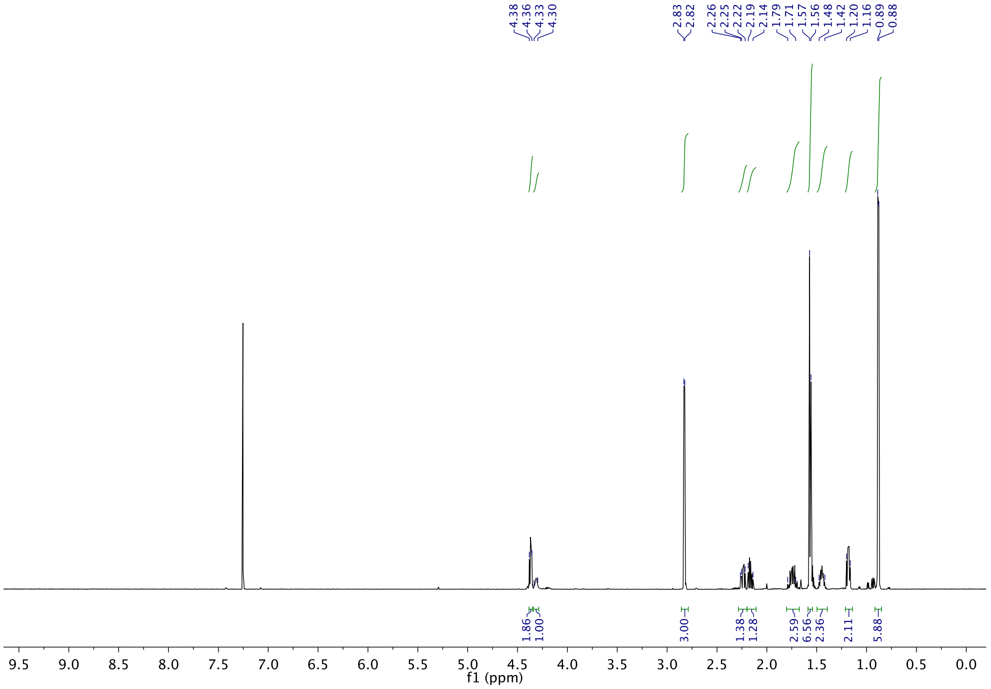

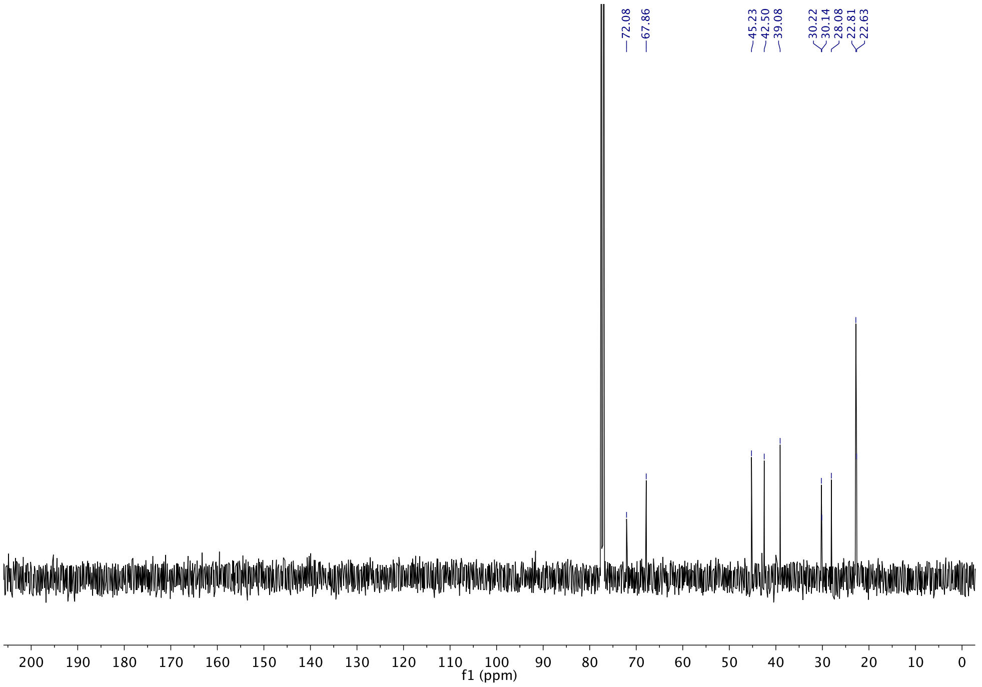

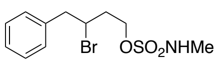
Br
OSO2NHMe

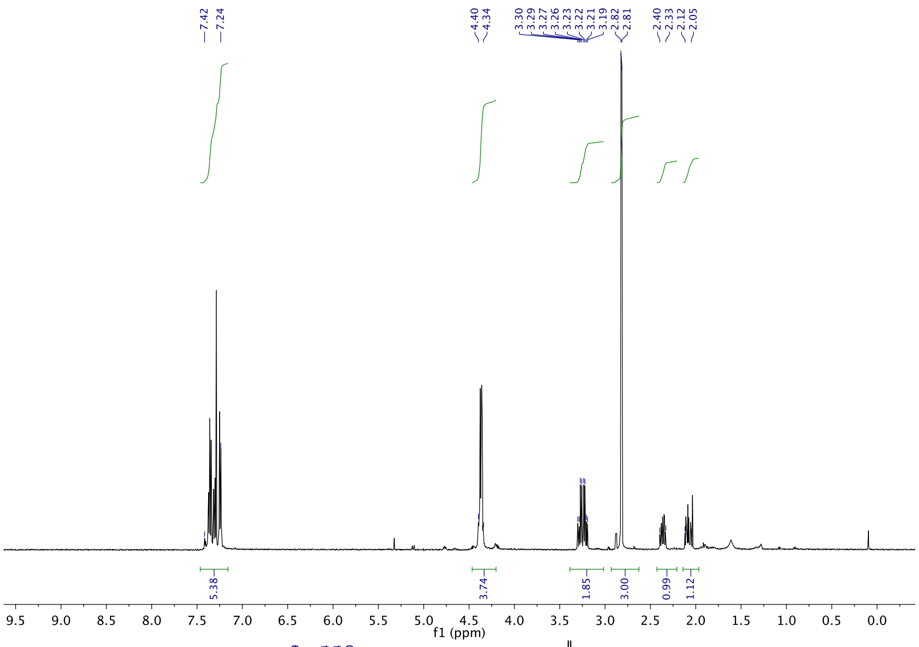
7.42
7.24
4.40
4.34
3.30
3.29
3.27
3.26
3.23
3.22
3.21
3.19
2.82
2.81
2.40
2.33
2.12
2.05
5.38
3.74
1.85
3.00
0.99
1.12
9.5 9.0 8.5 8.0 7.5 7.0 6.5 6.0 5.5 5.0 4.5 4.0 3.5 3.0 2.5 2.0 1.5 1.0 0.5 0.0
f1 (ppm)

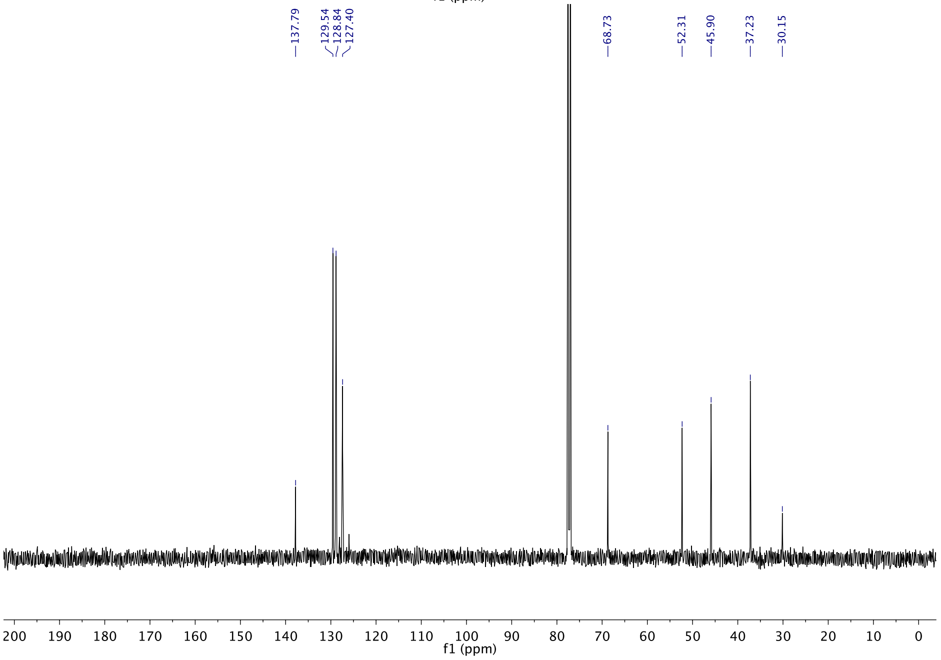
137.79
129.54
128.84
127.40
68.73
52.31
45.90
37.23
30.15
200 190 180 170 160 150 140 130 120 110 100 90 80 70 60 50 40 30 20 10 0
f1 (ppm)

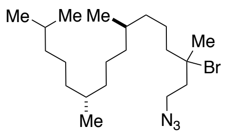
Me
Me Me
Me
Br
Me
N3

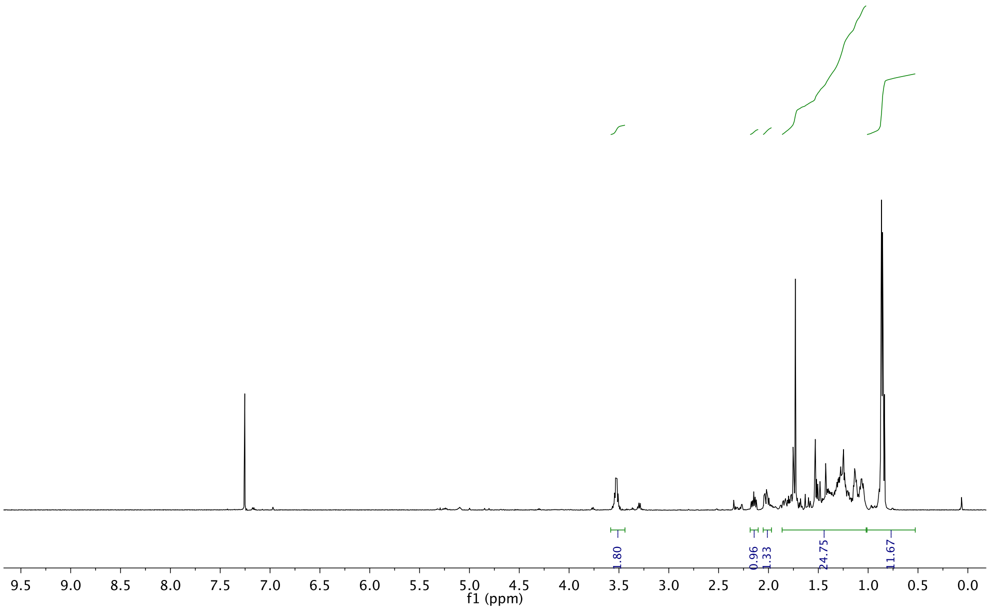
1.80
0.96
1.33
24.75
11.67
9.5 9.0 8.5 8.0 7.5 7.0 6.5 6.0 5.5 5.0 4.5 4.0 3.5 3.0 2.5 2.0 1.5 1.0 0.5 0.0
f1 (ppm)

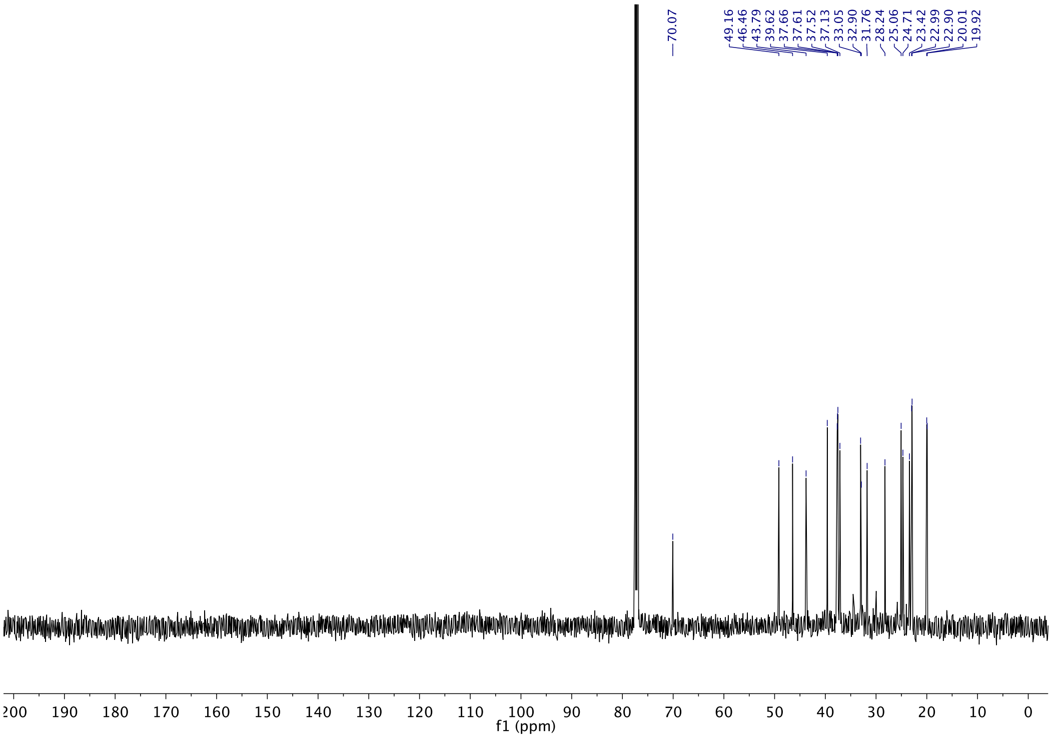
70.07
49.16
46.46
43.79
39.62
37.66
37.61
37.52
37.13
33.05
32.90
31.76
28.24
25.06
24.71
23.42
22.99
22.90
20.01
19.92
200 190 180 170 160 150 140 130 120 110 100 90 80 70 60 50 40 30 20 10 0
f1 (ppm)

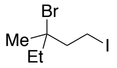
Br
Me
Et
I

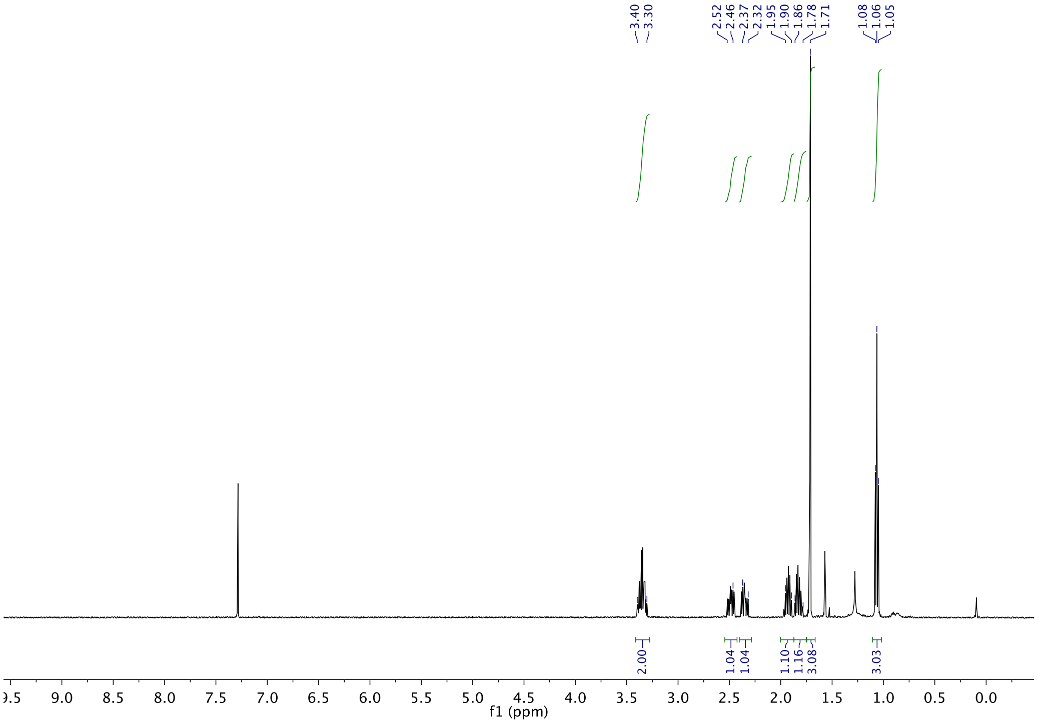
3.40
3.30
2.52
2.46
2.37
2.32
1.95
1.90
1.86
1.78
1.71
1.08
1.06
1.05
2.00
1.04
1.04
1.10
1.16
3.08
3.03
9.5 9.0 8.5 8.0 7.5 7.0 6.5 6.0 5.5 5.0 4.5 4.0 3.5 3.0 2.5 2.0 1.5 1.0 0.5 0.0
f1 (ppm)

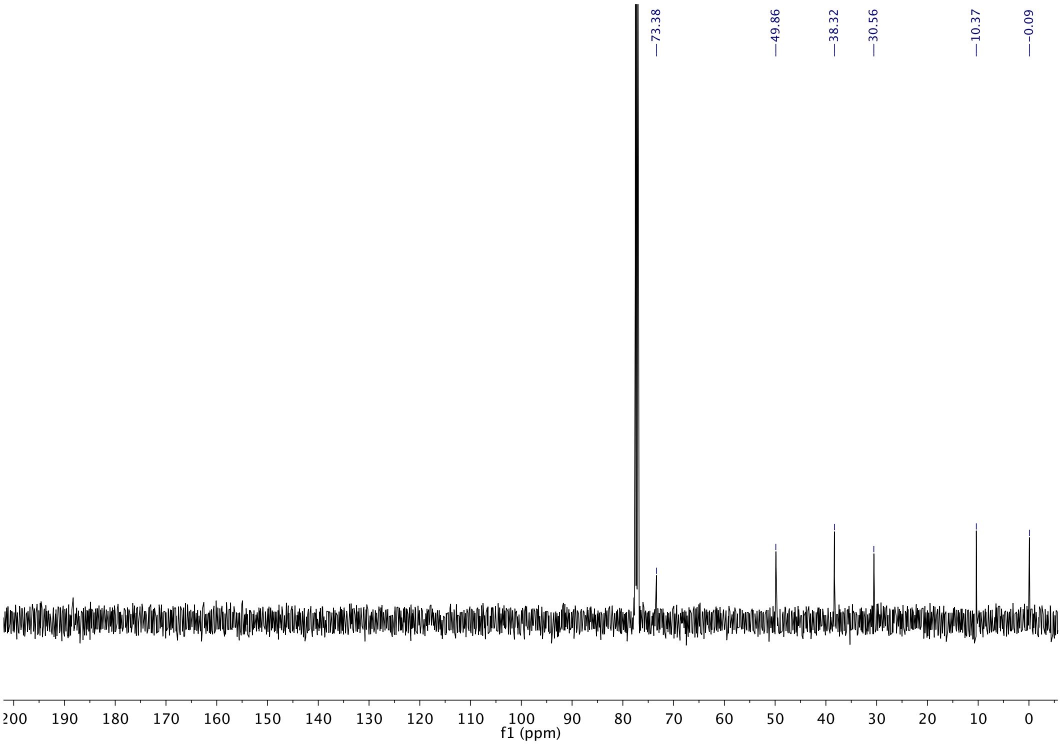
73.38
49.86
38.32
30.56
10.37
0.09
200 190 180 170 160 150 140 130 120 110 100 90 80 70 60 50 40 30 20 10 0
f1 (ppm)
